# Thermal annealing promoted room temperature phosphorescence: motion models and internal mechanism

**DOI:** 10.1093/nsr/nwad239

**Published:** 2023-09-15

**Authors:** Yan Gao, Jie Lu, Qiuyan Liao, Shuhui Li, Qianqian Li, Zhen Li

**Affiliations:** Hubei Key Lab on Organic and Polymeric Opto-Electronic Materials, TaiKang Center for Life and Medical Sciences, Department of Chemistry, Wuhan University, Wuhan 430072, China; Hubei Key Lab on Organic and Polymeric Opto-Electronic Materials, TaiKang Center for Life and Medical Sciences, Department of Chemistry, Wuhan University, Wuhan 430072, China; Hubei Key Lab on Organic and Polymeric Opto-Electronic Materials, TaiKang Center for Life and Medical Sciences, Department of Chemistry, Wuhan University, Wuhan 430072, China; Hubei Key Lab on Organic and Polymeric Opto-Electronic Materials, TaiKang Center for Life and Medical Sciences, Department of Chemistry, Wuhan University, Wuhan 430072, China; Hubei Key Lab on Organic and Polymeric Opto-Electronic Materials, TaiKang Center for Life and Medical Sciences, Department of Chemistry, Wuhan University, Wuhan 430072, China; Hubei Key Lab on Organic and Polymeric Opto-Electronic Materials, TaiKang Center for Life and Medical Sciences, Department of Chemistry, Wuhan University, Wuhan 430072, China

**Keywords:** room temperature phosphorescence, thermal annealing, structural reconfiguration, molecular motion, intermolecular interaction

## Abstract

Thermal annealing has been proven to be an efficient method to optimize the device performance of organic and polymeric opto-electronic materials. However, no detailed information of aggregate structures was obtained for a deeper understanding of what happens during thermal annealing. Herein, through modulation of molecular configurations by tunable linkage positions, and the amplified amplitudes of molecular motions by incorporation of additional methylene units, accurate changes of aggregated structures upon thermal annealing have been achieved, accompanying with the ‘turn-on’ room temperature phosphorescence (RTP) response by about 4800- and 177-fold increase of lifetimes. The stretching and swing motion models have been proposed, which afforded an efficient way to investigate the science of dynamic aggregation in depth.

## Teaser

Accurate changes of aggregated structures and motion models of organic luminogens during thermal annealing have been revealed by crystal engineering, accompanying with `turn-on' RTP response.

## INTRODUCTION

Due to their scientific and technological importance, organic and polymeric opto-electronic materials have attracted increasing interest and have been applied to lighting, sensing, displaying, information transferring, *etc* [1–11]. Thanks to the great efforts of scientists, many strategies have been explored and summarized as valuable guidance, and opto-electronic performance has been largely improved to meet practical requirements. Among them, thermal annealing is a common method to optimize film morphology [12,13], generally leading to dramatically improved performance of photovoltaic and field effect transistor devices [14–17]. However, what happens during the thermal annealing process? From the Grazing Incidence Wide Angle X-Ray Scattering (GIWAXS) results [18,19], some information about aggregated structures could be obtained, but this is not accurate in most cases, resulting in an unclear insight into the structure-packing-performance relationship of opto-electronic materials [20–22]. This, actually, badly hampers the further development of this research field, while the key thermal annealing process depends heavily on experience and/or attempts without explicit guidance. Thus, is it possible to get deep insight into the role of thermal annealing with accurate changes of aggregate structure?

Carefully considering the present analytical methods on aggregated structure, crystal analysis is the most powerful [23,24]. Based on single crystals with accurate aggregated structures, scientists can understand molecular aggregation step-by-step [24–28]. Perhaps, the influence of thermal annealing on aggregated structures might also be thoroughly investigated from crystals. So, how then to produce the molecular design and obtain the corresponding single crystals with optimized performance? Generally, accompanying the increase in temperature, the molecular motions will be more active, and the building blocks could have more chances to interact with each other. Also, it is needed in order to offer crystals with easily detected properties, which should be sensitive to the subtle changes of intermolecular interactions and aggregated structures. Accordingly, room temperature phosphorescence (RTP) of organic materials has been employed, which is heavily dependent on molecular aggregates for the sensitive triplet excitons. Thus, a thermal annealing treatment is applied to organic RTP materials. Many organic luminogens with crystalline states, including triphenylamine [29], phenothiazine [30,31] and acridine derivatives [32,33], were treated by thermal annealing under different temperatures (Supplementary Figure S1). However, even if the temperature increased above their melting points, the optimized RTP property can not be realized after the subsequent cooling process. This may be due to inadequate molecular motions, which cannot efficiently induce the reconfiguration process.

**Figure 1.**
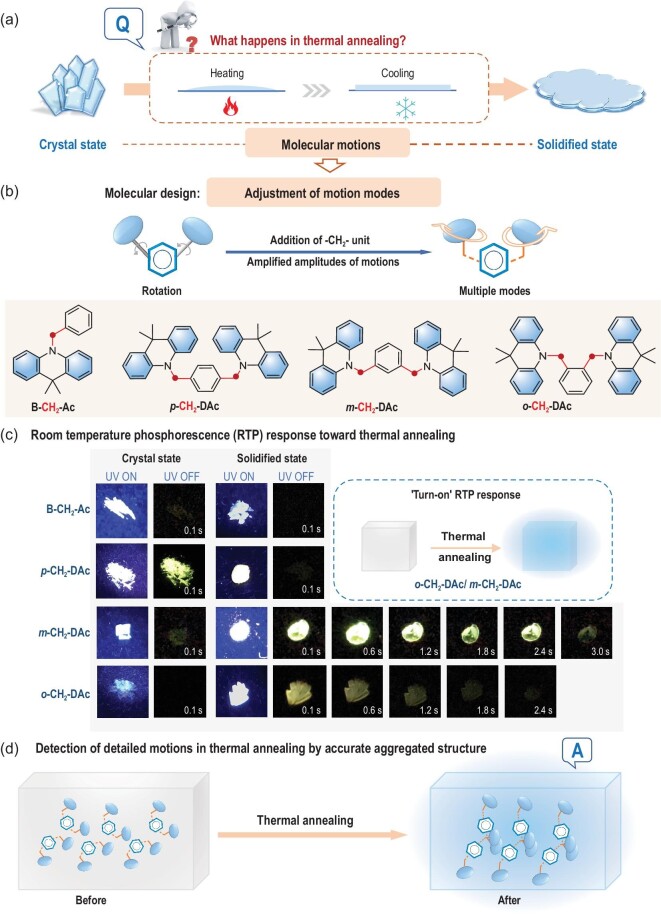
(a) Schematic diagram of the thermal annealing process from crystal to solidified state. (b) The design strategy of organic luminogens to achieve the large amplitudes of thermally molecular motions, and molecular structures of **B-CH_2_-Ac, *p*-CH_2_-DAc, *m*-CH_2_-DAc** and ***o*-CH_2_-DAc** with modulated arrangement of acridine moieties by different linkage positions. (c) Photographs of these luminogens at crystal and solidified states taken before and after UV irradiation (365 nm) under ambient conditions. (d) Detailed models of thermal motions by analysis of organic crystals before and after thermal annealing.

Thus, with the aim to achieve large differences of aggregated structures upon the thermal annealing process, it would be better to amplify the amplitudes of molecular motions, in order to promote the reconfiguration of molecular packing [34]. Accordingly, an additional methylene group was incorporated to accelerate the possible molecular motions and increase the amplitudes by multiple rotation modes (Fig. 1a). Moreover, the linkage modes of acridine as RTP moiety have been well-tuned by *o*-, *m*- and *p*-sites of phenyl core (Fig. 1b). Dynamic modulation by thermal annealing can motivate the reconfiguration of aggregated structures, resulting in a much improved RTP property at solidified states [19]. As shown in Fig. 1, the extremely weak RTP of ***m*-CH_2_-DAc** and ***o*-CH_2_-DAc** at crystal state changed to persistent and bright RTP emission at the solidified state due to thermal annealing. Accordingly, the RTP lifetime of***m*-CH_2_-DAc** increased from 1.54 ms (crystal state) to 272.36 ms (solidified state), and that of ***o*-CH_2_-DAc** increased from 0.056 ms to 267.07 ms.

More importantly, the accurate aggregated structures of ***o*-CH_2_-DAc** and ***m*-CH_2_-DAc** before and after thermal annealing have been successfully obtained, providing a unique platform to investigate in detail what happens during the annealing process. Accordingly, the internal mechanism and the corresponding motion models of organic molecules during thermal annealing have been rigorously investigated. Molecular ***o*-CH_2_-DAc** stretched up by steric repulsion, resulted in more compact molecular packing upon themal annealing, while acridine moieties in ***m*-CH_2_-DAc** swung subtly for the restriction of acridine-acridine interactions, promoting stronger intermolecular interactions. The corresponding dynamic structure-packing-performance relationship can promote the development of organic stimulus-responsive materials.

## RESULTS AND DISCUSSION

The target luminogens of ***p*-CH_2_-DAc, *m*-CH_2_-DAc** and ***o*-CH_2_-DAc** were synthesized by a one-step nucleophilic substitution reaction. Meanwhile, **B-CH_2_-Ac** bearing one acridine moiety was synthesized as reference (Supporting Information, Figure S2). These compounds were fully characterized by ^1^H NMR and ^13^C NMR spectroscopy (Supplementary Figures S3–S10), mass spectrometry, elemental analysis, HPLC (Supplementary Figure S11) and single-crystal X-ray diffraction (Supplementary Table S1).

All of them exhibited similar UV-vis absorption spectra in the diluted DCM solution (concentration: 10 *μ*M), with the absorption peaks located at about 290 nm (Supplementary Figure S12). They were mainly from acridine moieties with π-π* transitions, since the conjugation effect between acridine and phenyl moieties has been interrupted by methylene units. Accordingly, similar photoluminescence (PL) peaks at about 350 nm (Supplementary Figure S12), and phosphorescence emission wavelengths of 417 nm at 77 K (Supplementary Figure S13) were obtained in dilute DCM solution as isolated states (concentration: 10 *μ*M). Once these organic luminogens aggregated into single crystals, there is an obvious broadening of their absorption spectra (Supplementary Figure S14), mainly due to the multiple intermolecular interactions at aggregated states. These crystals exhibited a bright blue emission under UV irradiation, which was mainly from acridine moieties. The maximum emission wavelength of **B-CH_2_-Ac** with a single acridine moiety was located at 356 nm. Once two acridine moieties were linked to a phenyl core with different positions, the corresponding crystals demonstrated blue- or red-shifted emission with the emission wavelengths located at 371 nm, 354 nm and 345 nm for ***p*-CH_2_-DAc, *m*-CH_2_-DAc** and ***o*-CH_2_-DAc**, respectively. This can be explained by the different conformations of acridine moieties in these crystals (Supplementary Figure S15). For acridine moiety in **B-CH_2_-Ac** crystal, the dihedral angle of two phenyl moieties was 26.29°, while it decreased to 20.09° in ***p*-CH_2_-DAc** crystal with a more planar conformation, resulting in red-shift emission for a better conjugation effect. However, the larger dihedral angles of 27.86° and 37.66° with twisted conformations can be observed in ***m*-CH_2_-DAc** and ***o*-CH_2_-DAc** crystals. This was mainly due to the possible steric effect of two acridine moieties, resulting in blue-shifted emission for less conjugation effects. Regardless of the different conformations of acridine moieties, all of them demonstrated the separated HOMOs and LUMOs as charge transfer state (Supplementary Figure S16), benefiting RTP emission by the facilitated intersystem crossing (ISC) process. Organic luminogens bearing two acridine moieties exhibited higher photoluminescence quantum yields (*Ф*_PL_) of 11.02% (***p*-CH_2_-DAc**), 16.99% (***m*-CH_2_-DAc**) and 9.07% (***p*-CH_2_-DAc**), than that of **B-CH_2_-Ac** bearing a single one (6.91%).

**Figure 2.**
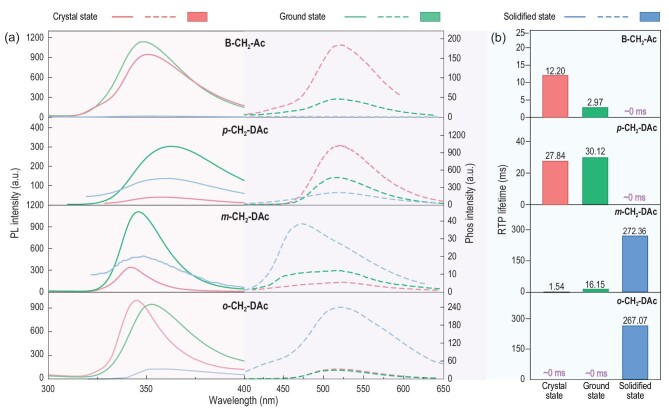
(a) Photoluminescence (solid line) and phosphorescence spectra (dotted line) of organic luminogens at crystal state (red), ground state (green) and solidified state (blue) at room temperature. (b) The corresponding phosphorescence lifetimes of organic luminogens in crystal state (red), ground state (green), and solidified state (blue) at room temperature.

After removing UV irradiation, only **B-CH_2_-Ac** and ***p*-CH_2_-DAc** crystals exhibited an observed yellow afterglow visible to the naked eye, while phosphorescence emission of ***m*-CH_2_-DAc** and ***o*-CH_2_-DAc** crystals was extremely weak and nearly invisible. As shown in Fig. 2, their phosphorescence spectra demonstrated similar emission peaks at about 515 nm, and RTP lifetimes of **B-CH_2_-Ac** and ***p*-CH_2_-DAc** can be detected with 12.20 ms and 27.84 ms, respectively (Supplementary Figure S17). The longer lifetime of ***p*-CH_2_-DAc** was mainly due to the parallel molecular arrangement with stronger intermolecular interactions among adjacent molecules. There were 8 kinds of C-H…π interactions (3.040 Å–3.914 Å), 4 kinds of C-H…N interactions (3.487 Å–3.754 Å), and 2 kinds of π-π interactions with the same π-π distances of 3.671 Å, and 18.35% and 18.27% overlap of the involved phenyl moieties, respectively (Supplementary Figures S18, S19 and S21). Accordingly, the extremely weak RTP property of ***m*-CH_2_-DAc** and ***o*-CH_2_-DAc** crystals was mainly due to the loose molecular packing for their twisted molecular conformations (Supplementary Figure S19, Figures S22–S24). However, on the other hand, the adequate space in aggregated structures may facilitate molecular motions with large amplitudes under external stimuli, including mechanical force and heating process [29,35].

Subsequently, when these crystals were broken by grinding at room temperature, the RTP lifetime of ***m*-CH_2_-DAc** increased from 1.54 ms to 16.15 ms, accompanied by prolonged lasting time of the afterglow and increased intensity of phosphorescence emission. ***p*-CH_2_-DAc** at ground state exhibited a similar lifetime (30.12 ms) to that at the crystal state (Fig. 2 and Table 1), and **B-CH_2_-Ac** demonstrated a decreased RTP lifetime from 12.20 ms to 2.97 ms due to the grinding process. These different variation trends indicated the varied change modes of aggregated structures under mechanical force, as partially proven by XRD patterns under different conditions (Supplementary Figure S25). Generally, diffraction peaks indicated a highly ordered molecular arrangement at crystalline states. After being ground, the diffraction peaks were weakened or disappeared due to the destruction of crystal structures, and there was nearly no significant change in the positions of diffraction peaks. This indicated that single crystals were mainly changed into micro-crystalline states or powders as amorphous states under mechanical force.

**Table 1.** Photophysical property of organic materials at room temperature.

Compound	λ_Abs_^a^ (nm)	λ_Fluo_^a^ (nm)	λ_Fluo_^b^ (nm)	λ_Phos_^b^ (nm)	τ_Phos_^b^ (ms)	Ф_PL_^b^ (%)	λ_Fluo_^c^ (nm)	λ_Phos_^c^ (nm)	τ_Phos_^c^ (ms)	Ф_PL_^c^ (%)	λ_phos_^d^ (nm)	τ_Phos_^d^ (ms)	Ф_PL_^d^ (%)
B-CH_2_-Ac	290	351	356	518	12.20	6.91	354	521	2.97	13.09	546	0.005	0.23
*p*-CH_2_-DAc	290	350	371	515	27.84	11.02	369	515	30.12	18.24	515	0.006	4.14
*m*-CH_2-_DAc	289	348	354	515	1.54	16.99	360	516	16.15	16.55	478	272.36	11.59
*o*-CH_2_-DAc	288	347	345	519	0.06	9.07	353	514	0.01	16.54	515	267.07	19.78

^a^In DCM solution with concentration of 10 μM. ^b^Crystal state. ^c^Ground state. ^d^Solidified state.

Also, heat was applied in order to accelerate molecular motion and modulate the molecular aggregates, and the resultant solidified state was obtained by the cooling process from the melting state via thermal annealing. Importantly, RTP properties of ***m*-CH_2_-DAc** and ***o*-CH_2_-DAc** at solidified state were largely improved, opposite to the quenching effect of thermal annealing on **B-CH_2_-Ac** and ***p*-CH_2_-DAc**, with largely decreased RTP lifetimes and intensities (Fig. 2 and Table 1). For ***m*-CH_2_-DAc**, the afterglow at crystal and ground state was nearly invisible to the naked eye, but it can last about 3.0 s at solidified state (Fig. 1). The RTP lifetime changed from 1.54 ms to 272.36 ms with 177-fold enhancement, accompanying the largely increased phosphorescence intensity (Fig. 2). ***o*-CH_2_-DAc** also exhibited a ‘turn-on’ RTP response toward thermal annealing, changing from undetectable phosphorescence at the initial crystal state to the persistent and bright afterglow with a lifetime up to 267.07 ms at solidified state. The possible degeneration during the thermal annealing process can be excluded by structure characterization, including ^1^H NMR spectroscopy (Supplementary Figures S26–S29) and HPLC (Supplementary Figure S30). Also, the possible effect of environmental factors can be excluded for the similar changes under nitrogen and air atmosphere (Supplementary Figure S31). Thus, the improved RTP property upon thermal annealing of ***m*-CH_2_-DAc** and ***o*-CH_2_-DAc** was mainly related to the re-configuration process of molecular aggregates. Furthermore, the dynamic modulation of aggregated structures have been optimized by the adjustment of thermal annealing processes with different heating and cooling rates, as well as the standing time at melting points (Supplementary Figures S32–S34). Accordingly, the rapid heating process, moderate standing time (15 s), and the rapid cooling process with the aid of cold packs are proved as the optimized thermal annealing treatment with efficient re-configuration process (Supplementary Video S1).

**Figure 3.**
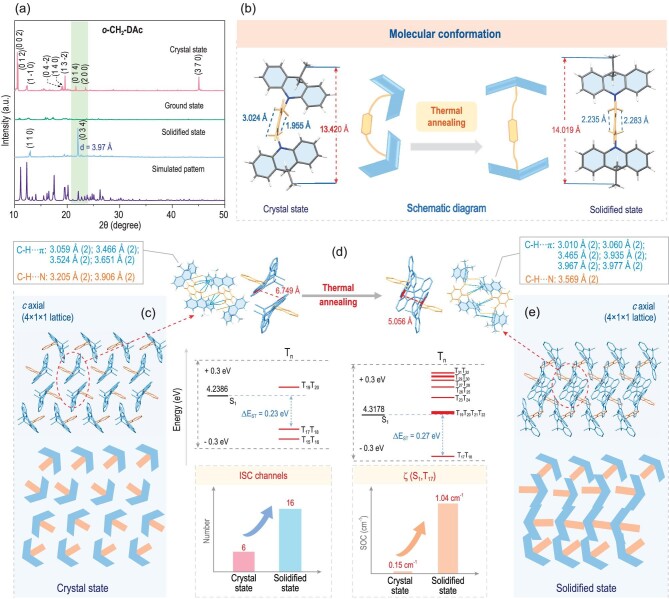
(a) Powder X-ray diffraction (PXRD) patterns of ***o*-CH_2_-DAc** (the simulated XRD pattern calculated from single-crystal X-ray data with Mercury 2022.2.0) at different aggregated states, including crystal state, ground state and solidified state. (b) Molecular conformations of ***o*-CH_2_-DAc** in the crystal state and solidified state, together with the schematic diagram. (c) Molecular packing of ***o*-CH_2_-DAc** crystal. (d) Analysis of intermolecular interactions and calculation of the corresponding energy levels and spin-orbit coupling constants for ***o*-CH_2_-DAc** dimer at crystal state and solidified state. (e) Molecular packing of ***o*-CH_2_-DAc** crystal at solidified state.

The changes of aggregated structures upon thermal annealing can be analyzed by XRD patterns under different conditions. The new diffraction peaks can only be detected in the solidified states of ***m*-CH_2_-DAc** and ***o*-CH_2_-DAc**, indicating the reconfiguration process with varied aggregated structures. For ***o*-CH_2_-DAc**, an emerged peak at 22.1° (2θ) in the XRD pattern demonstrated the formation of a new crystal face (034) (Fig. 3a) [6,36]. Fortunately, the detailed aggregated structures at solidified states can be further detected by X-ray single crystal diffraction. The cell volume decreased from 2926.7 Å^3^ (crystal state) to 2908 Å^3^ (solidified state), accompanied by increased density from 1.182 g/cm^3^ to 1.189 g/cm^3^. This is an indication of the more compact molecular packing upon thermal annealing (Supplementary Tables S1 and S2). Accordingly, the detailed variations from crystal to solidified states were analyzed by comparison of their molecular conformations and intermolecular interactions under different conditions. For ***o*-CH_2_-DAc** in single crystal as the initial state, the two acridine moieties were tilted with an asymmetric alignment to the phenyl core, as proved by the large difference in H…H distances of each methylene (-CH_2_-) with 1.955 Å and 3.024 Å, respectively (Fig. 3b). Since the shorter distance (1.955 Å) was smaller than twice of *van der Waals* Radii of hydrogen (∼1.1 Å) [37,38], a repulsive force can occur between them, as further confirmed by Reduced Density Gradient (RDG) calculations labeled by red cloud (Supplementary Figure S35) [39]. The repulsive force may induce the motions of methylene moieties, resulting in almost symmetric alignment with the similar H…H distance of 2.283 Å and 2.235 Å upon the thermal annealing process. The two acridine moieties at solidified states were almost parallel, and the distance of the two ending carbon atoms of the methyl group was 14.019 Å, much larger than that at the crystal state (13.420 Å), indicating that the whole molecular conformation of ***o*-CH_2_-DAc** had been largely stretched out (Fig. 3b, Video S2). Also, the two acridine moieties themselves demonstrated the planarization process, as proved by the decreased dihedral angles of two phenyl moieties from 30.30° and 32.31°, to 29.97° and 31.12° (Supplementary Figure S36), respectively.

Accordingly, molecular packing and intermolecular interactions varied subtly with the change in molecular conformations (Supplementary Figure S37, Table S3 and Video S2). For instance, taking the adjacent molecule with stronger intermolecular interactions (Fig. 3d), the distance between the involved phenyl rings of acridine moieties decreased from 6.749 Å (crystal state) to 5.056 Å (solidified state), indicating more compact aggregated structures. Accordingly, C-H…π interactions between methylene (C-H) and acridine moieties (π) varied with the corresponding distances changing from 3.059 Å–3.651 Å to 3.060 Å–3.977 Å. Moreover, 3 kinds of C-H…π interactions between methyl units (C-H) and another acridine moiety (π) were newly formed with the distances of 3.010 Å–3.967 Å (Supplementary Table S4). These variations caused by thermal annealing can suppress molecular motions via stronger intermolecular interactions, contributing to the improved RTP property of ***o*-CH_2_-DAc** at aggregated states. Moreover, the promoted RTP property with the facilitated ISC process can be confirmed by theoretical calculation. Generally, the possible ISC channels are mainly determined by the small energy gaps (<0.3 eV), and the large spin-orbit coupling constants (SOC) between T*_m_* and S_1_. The number of ISC processes increased from 6 (crystal state) to 16 (solidified state), and the spin-orbit coupling (SOC) constant of the major ISC channel ζ (S_1_, T_17_) enhanced from 0.15 cm^−1^ (crystal state) to 1.04 cm^−1^ (solidified state) (Fig. 3d). Although the energy gap (ΔE_ST_) between S_1_ and T_18_ as the lowest one slightly increased from 0.23 eV (crystal state) to 0.27 eV (solidified state), the positive effect from the increased channels and improved SOC values played the dominant role in facilitating the ISC process (Supplementary Figure S38 and Table S5).

**Figure 4.**
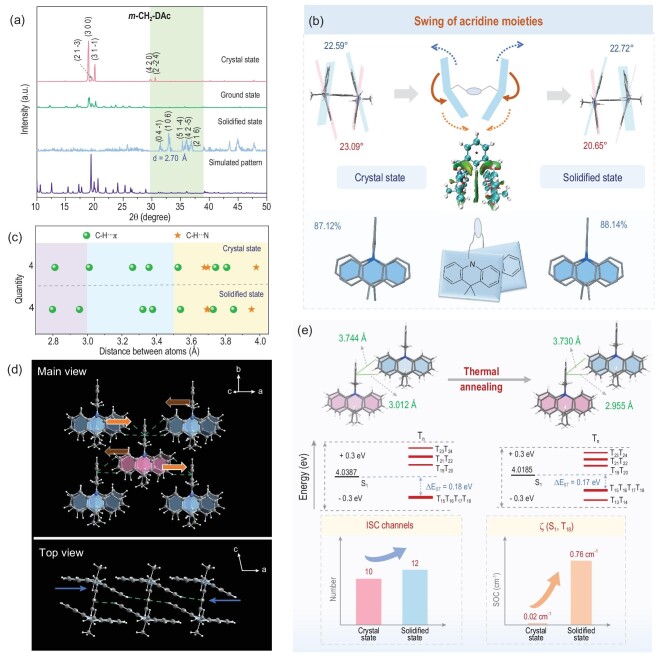
(a) Powder X-ray diffraction (PXRD) patterns for ***m*-CH_2_-DAc** (the simulated XRD pattern calculated from single-crystal X-ray data with Mercury 2022.2.0) at different aggregated states, including crystal state, ground state and solidified state. (b) Molecular conformations of ***m*-CH_2_-DAc** in the crystal state and solidified state, including the dihedral angles of two phenyl moieties in each acridine moiety and the overlap of acridine moieties under different conditions, and the gradient isosurfaces of ***m*-CH_2_-DAc** at crystal state. (c) The kinds and distances of intermolecular interactions between adjacent ***m*-CH_2_-DAc** molecules at the crystal and solidified state. (d) Molecular packing and intermolecular interactions in ***m*-CH_2_-DAc** crystals under different views. (e) Analysis of intermolecular interactions, and calculation of the corresponding energy levels and spin-orbit coupling constants for ***m*-CH_2_-DAc** dimer at both crystal and solidified states.

The improved RTP property of ***m*-CH_2_-DAc** crystals by thermal annealing can also be explained by XRD patterns and crystal structures under different conditions. As shown in Fig. 4a, multiple diffraction peaks had emerged in the high-angle (30°∼40°) region at solidified state, an indication of different molecular packing after thermal annealing, compared to those at crystal state. The corresponding changes of aggregated structures were investigated in detail by X-ray single crystal diffraction, including the trajectory of molecular motions simulated by Gaussian View 6 (Video S3), and varied intermolecular interactions. For ***m*-CH_2_-DAc** at the crystal state, the two acridine moieties were nearly perpendicular to the phenyl core, and parallel to each other with a large overlap (87.12%). Combined with their close distance (4.010 Å), the *van der Waals* forces between two acridine moieties can be formed (Supplementary Figure S39), as proved by the RDG calculations labeled by green cloud (Fig. 4b).

After thermal annealing, conformation of acridine moieties themselves remains almost the same, but the whole molecular conformation changes for the swing of acridine moieties around the phenyl core. This mode of molecular motion may be related to the acridine-acridine interactions through space, which can be detected by the varied dihedral angles of phenyl units in each acridine moiety, changing from 22.59° and 23.09° (crystal state) to 20.65° and 22.72° (solidified state), respectively. Also, the overlap of acridine moieties increased from 87.12% to 88.14%, accompanying with the decreased distance between the centroid of two acridine moieties from 4.130 Å to 4.104 Å (Supplementary Figure S40). Accordingly, intermolecular interactions varied subtly with the swing of acridine moieties (Supplementary Tables S6–S13). As shown in Fig. 4c, most of the involved distances in C-H…π and C-H…N interactions decreased, while the whole quantity of intermolecular interactions remained the same (Supplementary Figure S41 and Table S14). The detailed changes can be reflected by the C-H…π interactions between the phenyl core as π system and C-H bonding of acridine moiety from the adjacent molecules (Fig. 4d and e). The corresponding distances decreased from 3.012 Å to 2.955 Å, and from 3.744 Å to 3.730 Å, respectively, resulting in the closer arrangement of ***m*-CH_2_-DAc** along the *a* axis (Fig. 4d). It was mainly related to the swing of two acridine moieties from different directions during the re-configuration process.

Accordingly, the resultant RTP property with a longer lifetime can be explained by theoretical calculations based on the representative dimer. The number of ISC channels increased from 10 to 12, and most ISC processes demonstrated enhanced SOC values (Fig. 4e, Supplementary Figure S42 and Table S15). For ISC channel from S_1_ to T_18_ with the lowest energy gaps, the corresponding SOC constant ζ (S_1_, T_18_) increased from 0.02 cm^−1^ to 0.76 cm^−1^ with slightly decreased energy gaps (Supplementary Figure S43 and Table S16). Thus, the modulation of ***m*-CH_2_-DAc** crystal by thermal annealing mainly focuses on the slight swing of acridine moieties, which are restricted by the *van der Waals* forces between them, resulting in stronger intermolecular interactions with closer distances. It can facilitate the ISC processes with more channels and larger SOC, promoting the bright and persistent RTP property at the solidified state.

Since the improved RTP property upon thermal annealing can only be realized by***o*-CH_2_-DAc** and ***m*-CH_2_-DAc** crystals, the possible universality of *o*- and *m*-substitution modes was further investigated by the replacement of acridine (Ac) moieties with phenothiazine 5,5-dioxide (OCs) units. Accordingly, the molecules **B-CH_2_-OCs, *p*-CH_2_-DOCs, *m*-CH_2_-DOCs** and ***o*-CH_2_-DOCs** have been synthesized (detailed procedures and characterization are shown in the supporting information, Figures S44–S52), which correspond to B-CH_2_-Ac, *p*-CH_2_-DAc, *o*-CH_2_-DAc and *m*-CH_2_-DAc, respectively. After the thermal annealing process, similar phenomena can be detected (Supplementary Figure S53). In detail, *m*-CH_2_-DOCs and *o*-CH_2_-DOCs demonstrated the improved RTP property from crystal state to solidified state upon thermal annealing, as confirmed by the increased RTP lifetimes from 15.89 ms to 46.89 ms for *m*-CH_2_-DOCs and from 17.86 ms to 63.12 ms for *o*-CH_2_-DOCs. The quenching effect of thermal annealing on B-CH_2_-OCs and *p*-CH_2_-DOCs crystals is obvious, which demonstrated decreased RTP intensities and lifetimes (Supplementary Figures S54 and S55), for instance, the RTP lifetime of *p*-CH_2_-DOCs decreased from 22.28 ms to 4.27 ms upon thermal annealing.

**Figure 5.**
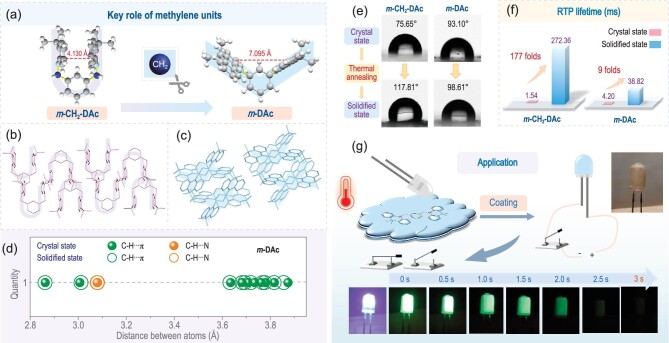
(a) Molecular structures of ***m*-CH_2_-DAc** and ***m*-DAc** with comparison. (b) Molecular packing of ***m*-CH_2_-DAc** at the crystal state. (c) Molecular packing of ***m*-DAc** at the crystal state. (d) Analysis of intermolecular interactions for ***m*-DAc** dimer at both crystal and solidified states. (e) Water contact angles of ***m*-CH_2_-DAc** and***m*-DAc** under different conditions. (f) RTP lifetimes of ***m*-CH_2_-DAc** and ***m*-DAc** under different conditions. (g) The application of ***m*-CH_2_-DAc** with solidified states as a surface coating for light emitting diodes.

Furthermore, with the aim of investigating the key role of linkage modes in the changeable RTP property under different conditions, the molecule ***m*-DAc** with direct linkage of two acridine moieties to the *m*-positions of the phenyl core by *Carbon-Nitrogen* bonding was synthesized (Scheme S1) and well characterized by ^1^H NMR spectra and HPLC (Supplementary Figures S56–S58). It was employed as the reference to***m*-CH_2_-DAc** for their similar molecular structures (Fig. 5a), therefore the role of additional methylene units can be investigated. From molecular configuration of ***m*-DAc** in single crystals, the dihedral angles of acridine and phenyl moieties were 119.54^o^ and 119.95^o^, respectively, indicating less π-π conjugation between them (Supplementary Figure S59). The similar swing model with ***m*-CH_2_-DAc** can be found by the rotations of the *Carbon-Nitrogen* bond, as proved by the decreased distances of two acridine moieties from 6.375 Å to 6.315 Å along one side, together with the increased distances from 4.386 Å to 4.577 Å along the other side, respectively (Supplementary Figure S60). Upon thermal annealing, the dihedral angles between acridine and phenyl moieties are 119.55^o^ and 119.48^o^ with subtle changes (Supplementary Figure S60). Accordingly, the RTP improved subtly, as illustrated by the increase in phosphorescence intensity and prolonged afterglow (Supplementary Figures S61 and S62). The RTP lifetimes exhibited a 9-fold enhancement from 4.20 ms (crystal state) to 38.82 ms (solidified state) (Supplementary Figure S63), much smaller than that of ***m*-CH_2_-DAc** with 177-fold enhancement (Fig. 5f).

Possible changes in aggregated structures can be detected by the variations in water contact angles before and after thermal annealing, which demonstrated the obvious variation from 75.65° (crystal state) to 117.81° (solidified state) for ***m*-CH_2_-DAc**, while those of ***m*-DAc** were similar with 93.10° (crystal state) and 98.61° (solidified state), respectively. This indicated much larger changes of ***m*-CH_2_-DAc** in aggregated structures during thermal annealing (Fig. 5e) [40,41], which can be partially explained by their molecular conformations and intermolecular interactions before and after thermal annealing.

For molecular conformation of ***m*-CH_2_-DAc** with ‘U’ shape, the molecular packing is loose for the twisted conformation, and the corresponding intermolecular interactions were relatively weak (Fig. 5b). There were only 28 kinds of C-H…π interaction (2.812 Å–3.808 Å) and 12 kinds of C-H…N interaction (3.679 Å–3.979 Å) between a given molecule and the adjacent ones (Supplementary Figure S41). Thus, the thermal molecular motions can be active, facilitating the reconfiguration process. As to ***m*-DAc** with ‘V’ shape, the compact molecular packing is formed with an interlaced mode (Fig. 5c, Supplementary Figures S64 and S65), resulting in stronger intermolecular interactions among adjacent molecules. There were 48 kinds of C-H…π interaction (2.739–3.954 A˚) and 14 kinds of C-H…N interaction (3.043–3.949 A˚) among adjacent molecules (Supplementary Figure S65 and Table S17). This can constrain molecular motions, which adversely affects the reconfiguration process, and can be further confirmed by the nearly unchanged intermolecular interactions upon thermal annealing (Fig. 5d and Supplementary Table S18). The low amplitude motions of organic luminogens cannot induce the efficient reconfiguration process to achieve a large RTP property enhancement.

Thus, the addition of methylene unit is beneficial to the motions of RTP moieties with large amplitudes, which can facilitate the regulation of molecular packing during thermal annealing processes. However, if more methylene units were incorporated by *tri*-substituted and *tetra*-substituted structures (*t*-CH_2_-Ac and *q*-CH_2_-Ac), or the increased lengths of alkyl chains (*m*-3CH_2_-DAc) (detailed procedures and characterization are shown in the supporting information, Figures S66–S71), the promoted RTP property upon thermal annealing cannot be realized (Supplementary Figures S72 and S73). This may be due to the unfavorable molecular packing and/or flexible structures with severe molecular motions. Therefore, the methylene unit is essential to the improved RTP property by thermal annealing, but the incorporation of excess methylene units is not the preferred strategy.

With the systematic investigation of the relation between motion models of organic luminogens and their RTP property, some efficient strategies can be concluded as follows. (1) Special intramolecular interactions should be constructed into the molecular design of organic luminogens, in order to make the directional molecular motions with ordered changes in aggregated structures. Apart from the construction of repulsive or attractive force by the adjustment of molecular configurations, some self-assemble process may be employed as the driving force. Thus, the crystalline states can be maintained in most cases, facilitating to the formation of optimized molecular packing for the RTP property. (2) The loose molecular packing can afford more space for the thermal motions of organic luminogens with large amplitudes to proceed, which can promote the variations of RTP property for the large changes in aggregated structures. Through the formation of preferred packing modes, the improved RTP property can be achieved by thermal-annealing processes.

Finally, the thermal annealing promoted RTP property of ***m*-CH_2_-DAc** demonstrated good repeatability. The RTP property can still be maintained after five cycles of thermal annealing processes (Video S4). Thus, combined with the excellent RTP property of ***m*-CH_2_**-**DAc** at solidified state and the fluidity of molten state, it could be effectively applied as a flexible afterglow coating (Fig. 5g). Accordingly, ***m*-CH_2_-DAc** was heated above its melting point (218–220°C), and coated on the surface of a light-emitting diode (LED) (365 nm, 0.06 W), which would form a uniform film after cooling. When voltage (3 V) was applied, the coated LED displayed blue-violet fluorescence. Once the voltage was removed, the bright green afterglow would appear, which can last for up to 3 s. It not only realized the flexible packaging of LEDs, but also the multi-color emission under different conditions.

## CONCLUSION

In summary, the detailed motion models of organic luminogens upon thermal annealing have been captured by crystal engineering, contributing to the understanding of the internal mechanism for thermal modulation. Moreover, it can be well-controlled by the initial molecular conformations, resulting in the stretching and swing modes in ***o*-CH_2_-DAc** and ***m*-CH_2_-DAc** crystals, respectively. The stretching mode is mainly due to the repulsive force of methylene moieties, and the swing is dominated by acridine-acridine interactions. These molecular motions can be beneficial in forming the compact molecular packing, and strengthen the intermolecular interactions, that result in the promoted ISC process and stabilization of excited triplet states. Thus, RTP lifetimes can be largely increased by about 4800- and 177-fold for ***o*-CH_2_-DAc** and ***m*-CH_2_-DAc**, respectively. The ‘turn-on’ response upon thermal annealing can be incorporated into the coating technology to achieve multicolor displaying. The visualization of molecular thermal motions with accurate aggregated structures afforded an efficient way to investigate, in depth, the science of dynamic molecular aggregation under external force.

## METHODS

### Theoretical calculation

The time-dependent density functional theory (TD-DFT) calculations were performed on Gaussian 09 program (Revision D.01) [42]. The excitation energies in the n-th singlet (S_n_) and n-th triplet (T_n_) monomer states were obtained using the PBE1PBE/def2svp based on the structure extracted from a single crystal. The trajectory of molecules were simulated in Gaussian View 6.0 [43]. Spin-orbit coupling (SOC) was conducted with the PySOC package and based on the excited states from Gaussian 09. The reduced density gradient (RDG) function of the ground state molecule was analyzed using the Multifwn 3.8 (dev) [39]. Non-covalent interactions within the molecule were plotted using the Visual Molecular Dynamics (VMD) program [44] to facilitate a more visual analysis of these interactions. The scatter diagram for RDG versus sign(*λ_2_*)*ρ* was conducted with gnuplot software.

## Supplementary Material

nwad239_Supplemental_File

additional_CIF-DATA_and_CIF-data

nwad239_Video_S1-optimized_thermal_annealing_process

nwad239_Video_S2-Trajectory_of_molecule_o-CH2-DAc

nwad239_Video_S3-Trajectory_of_molecule_m-CH2-DAc

nwad239_Video_S4-Repeatability_experiments
