# Supplementary material for: Thermal annealing promoted room temperature phosphorescence: motion models and internal mechanism
Source: Natl Sci Rev. 2023 Sep 15;10(11):nwad239. doi: 10.1093/nsr/nwad239 (PMC10581540; doi:10.1093/nsr/nwad239)
Supplement: nwad239_Supplemental_File [file nwad239_supplemental_file.pdf]

# Thermal Annealing Promoted Room Temperature Phosphorescence: Motion Models and Internal Mechanism

Yan Gao, Jie Lu, Qiuyan Liao, Shuhui Li, Qianqian Li* and Zhen Li*

Hubei Key Lab on Organic and Polymeric Opto-Electronic Materials, TaiKang Center for Life and Medical Sciences, Department of Chemistry, Wuhan University, Wuhan 430072 (China).

*Corresponding authors. E-mail: liqianqian@whu.edu.cn; lizhen@whu.edu.cn

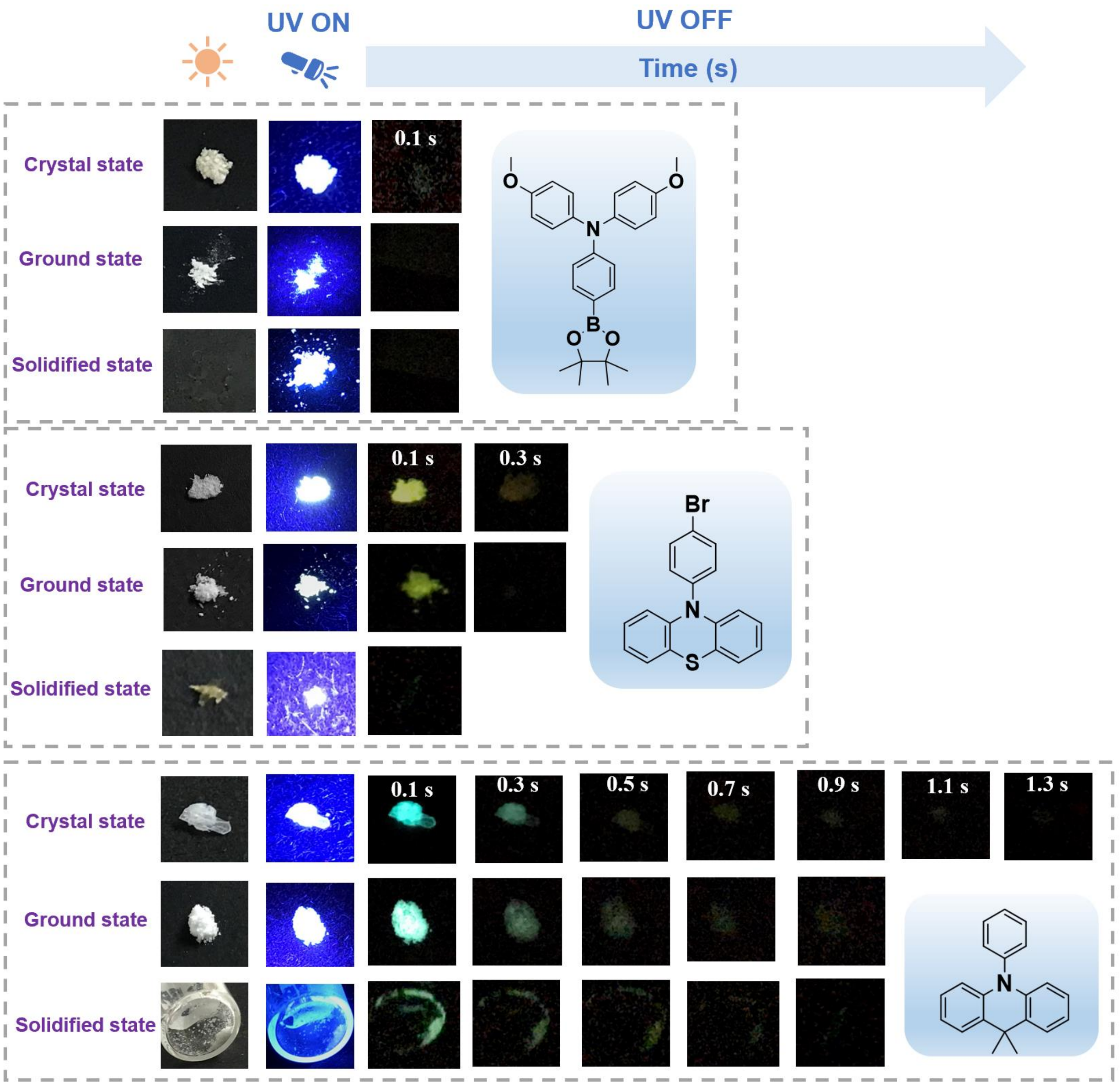


**Figure S1.** Photographs of triphenylamine, phenothiazine, and acridine derivatives at crystal state, ground state, and solidified state, taken before and after UV irradiation (365 nm) under ambient conditions.

## Experimental section

### 1.1 Materials:

Toluene was dried by sodium-potassium alloy and stored under an atmosphere of nitrogen. All other chemical reagents were purchased from commercial sources, and used for reaction or testing without further purification.

## 1.2 Characterization

$^{1}H$ and $^{13}C$ NMR spectra were recorded on a Bruker Avance III HD 400 MHz using tetramethylsilane (TMS; δ = 0 ppm) as internal standard. Elemental analyses were performed by a Perkin-Elmer microanalyzer. Mass spectra were measured on a ZAB 3F-HF mass spectrophotometer. UV-vis absorption spectra were conducted on a Shimadzu UV-2550 spectrometer. Fluorescence and phosphorescence spectra were performed on a Hitachi F-4700 fluorescence spectrophotometer. Quantum yields and lifetimes were determined with FLS-980 spectrometer. The powder X-ray diffraction patterns were recorded by D8 Advanced (Bruker) using Cu-Kα radiation from 10° to 50°. The single-crystal X-ray diffraction data of organic crystals were collected in an XtaLAB Synergy Custom single crystal diffractometer. High-performance liquid chromatography (HPLC) of organic crystals were conducted on Waters 600. High performance liquid chromatography (HPLC) of solidified samples were performed on LaboACE LC-500. Melting points were performed on Ry-1G melting point apparatus.

## 1.3 Synthesis

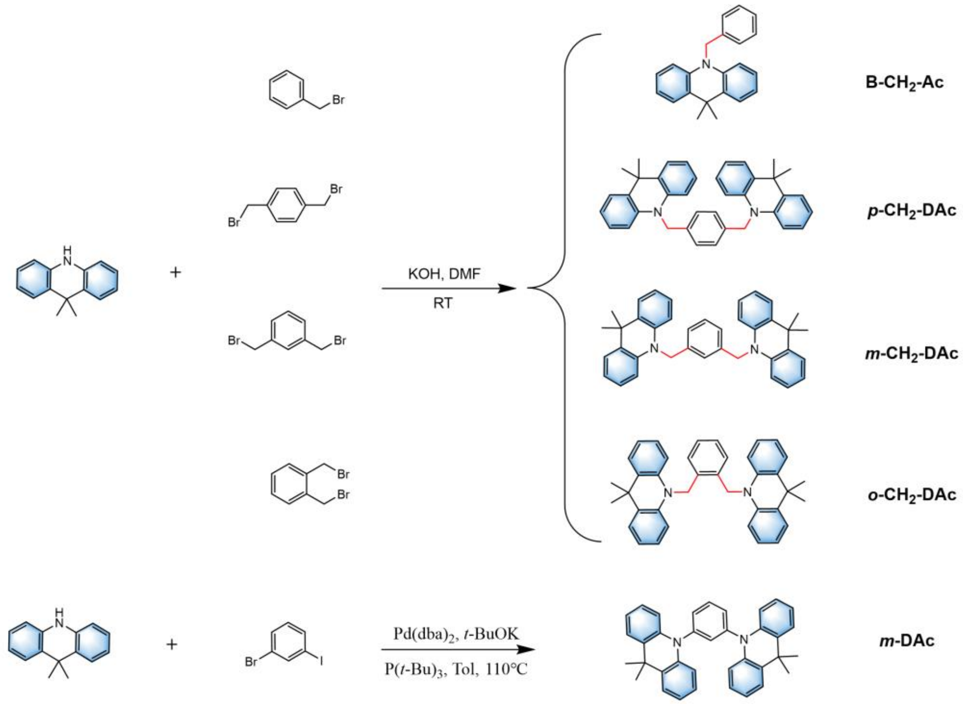


**Figure S2.** The synthetic routes of B-$CH_2$-Ac, *p*-$CH_2$-DAc, *m*-$CH_2$-DAc, *o*-$CH_2$-DAc and *m*-DAc.

### Synthesis of B-$CH_2$-Ac

9,10-Dihydro-9,9-dimethylacridine (100 mg, 0.48 mmol) and potassium hydroxide (268 mg, 4.70 mol) were placed in a round bottom flask, dissolved with dimethyl sulfoxide (10 mL), and stirred at room temperature for 1 h. After this, benzyl bromide (0.07 mL, 0.62 mmol) were added and stirred at room temperature for 12 h. Then, a large amount of $H_2O$ was added, and then the filter residue was collected after filtration. The crude product was purified by a silica gel column using petroleum ether and dichloromethane as eluent to give **B-$CH_2$-Ac** as a light white solid (125 mg, 88%). mp: 221 ℃. $^1$H NMR (400 MHz, Chloroform-*d*) δ 7.46 (dd, *J* = 7.7, 1.6 Hz, 2H, ArH), 7.33 (dd, *J* = 8.1, 6.4 Hz, 2H, ArH), 7.28 (d, *J* = 1.5 Hz, 1H, ArH), 7.24-7.20 (m, 2H, ArH), 7.11-7.05 (m, 2H, ArH), 6.97 (td, *J* = 7.5, 1.2 Hz, 2H, ArH), 6.75 (dd, *J* = 8.1, 1.2 Hz, 2H, ArH), 5.21 (s, 2H, -$CH_2$), 1.62 (s, 6H, -$CH_3$). $^{13}$C NMR (100 MHz, Chloroform-*d*) δ 140.67, 137.09, 132.06, 128.76, 126.86,

126.73, 126.34, 124.39, 120.70, 113.27, 77.24, 51.25, 36.25, 29.53. HRMS (EI) calcd. for $C_{22}H_{21}N$ $[M+H]^+$: 300.17; Found: 300.23. Anal. calcd. for $C_{22}H_{21}N$: C, 88.25; H, 7.07; N, 4.68. Found: C, 87.89; H, 7.21; N, 4.92.

**The general synthesis route of *p*-$CH_2$-DAc, *m*-$CH_2$-DAc and *o*-$CH_2$-DAc**

9,10-Dihydro-9,9-dimethylacridine (2.0 equiv.) and potassium hydroxide (10.0 equiv.) were placed in a round bottom flask, dissolved with dimethyl sulfoxide (10 mL), and stirred at room temperature for 1 h. Dibromomethyl benzene derivatives (1.0 equiv.) were added in small amounts in batches and stirred at room temperature for 12 h. Then, a large amount of $H_2O$ was added, and then the filter residue was collected after filtration. The crude product was purified by a silica gel column using petroleum ether and dichloromethane as eluent to obtain the desired materials.

***p*-$CH_2$-DAc:** A white solid (118 mg, 49%). mp: 278 ℃. $^1H$ NMR (400 MHz, Chloroform-*d*) δ 7.45 (dd, *J* = 7.7, 1.5 Hz, 4H, ArH), 7.16 (s, 4H, ArH), 7.10 (td, *J* = 9.2, 7.8, 1.5 Hz, 4H, ArH), 6.97 (t, *J* = 7.6 Hz, 4H, ArH), 6.77 (d, *J* = 8.2 Hz, 4H, ArH), 5.18 (s, 4H, -$CH_2$), 1.61 (s, 12H, -$CH_3$). $^{13}C$ NMR (100 MHz, Chloroform-*d*) δ 140.64, 135.81, 132.06, 126.78, 126.73, 124.44, 120.70, 113.26, 77.24, 51.01, 36.23, 29.59. HRMS (EI) calcd. for $C_{38}H_{36}N_2$ $[M+H]^+$: 521.29; Found: 521.35. Anal. calcd. for $C_{38}H_{36}N_2$: C, 87.65; H, 6.97; N, 5.38. Found: C, 88.07; H, 6.87; N, 5.40.

***m*-$CH_2$-DAc:** A white solid (136 mg, 56%). mp: 218 ℃. $^1H$ NMR (400 MHz, Chloroform-*d*) δ 7.42 (dd, *J* = 7.7, 1.6 Hz, 4H, ArH), 7.23 (d, *J* = 7.4 Hz, 1H, ArH), 7.11 (d, *J* = 3.7 Hz, 2H, ArH), 7.09 (s, 1H, ArH), 7.05 (td, *J* = 7.7, 1.5 Hz, 4H, ArH), 6.95 (td, *J* = 7.5, 1.2 Hz, 4H, ArH), 6.71 (dd, *J* = 8.1, 1.1 Hz, 4H, ArH), 5.13 (s, 4H, -$CH_2$), 1.57 (s, 12H, -$CH_3$). $^{13}C$ NMR (100 MHz, Chloroform-*d*) δ 140.64, 137.96, 132.07, 129.20, 126.65, 125.04, 124.41, 124.34, 120.67, 113.23, 51.17, 36.20, 29.41.

HRMS (EI) calcd. for $C_{38}H_{36}N_2$ $[M+H]^+$: 521.29; Found: 521.35. Anal. calcd. for $C_{38}H_{36}N_2$: C, 87.65; H, 6.97; N, 5.38. Found: C, 88.03; H, 6.97; N, 5.37.

***o*-CH$_2$-DAc:** A white solid (142 mg, 59%). mp: 248 ℃. $^1$H NMR (400 MHz, Chloroform-*d*) δ 7.51 (dd, *J* = 7.8, 1.5 Hz, 4H, ArH), 7.17 (m, 4H, ArH), 7.12 (*t*, *J* = 3.4 Hz, 4H, ArH), 7.02 (td, *J* = 7.5, 1.1 Hz, 4H, ArH), 6.78 (dd, *J* = 8.2, 1.2 Hz, 4H, ArH), 5.28 (s, 4H, $-CH_2$), 1.66 (s, 12H, $-CH_3$). $^{13}$C NMR (100 MHz, Chloroform-*d*) δ 140.47, 132.82, 132.26, 127.24, 127.20, 126.88, 124.63, 120.97, 113.17, 49.23, 36.30, 29.79. HRMS (EI) calcd. for $C_{38}H_{36}N_2$ $[M+H]^+$: 521.29; Found: 521.35. Anal. calcd. for $C_{38}H_{36}N_2$: C, 87.65; H, 6.97; N, 5.38. Found: C, 88.04; H, 7.00; N, 5.38.

**Synthesis of *m*-DAc**

Under an atmosphere of nitrogen, a mixture of 1-bromo-3-iodobenzene (430 mg, 1.50 mmol), 9,9-dimethyl-9,10-dihydroacridine (700 mg, 3.30 mmol), palladium acetate (37.5 mg, 0.17 mmol), tri-tert-butylphosphine (10 mg, 0.05 mmol) and sodium tert-butoxide (584 mg, 6.00 mmol) was placed in dry and degassed toluene (20 mL) and refluxed overnight at 110 $^o$C. After being cooled to room temperature, the reaction mixture was treated with brine and then the solution was extracted with DCM and $H_2O$ for three times. The organic layer was dried over anhydrous $Na_2SO_4$ and concentrated. The crude product was purified by column chromatography on silica gel using petroleum ether as eluent to obtain ***m*-DAc** as a light white solid (420 mg, 56%). mp: 221℃. $^1$H NMR (400 MHz, Chloroform-*d*) δ 7.90 (t, *J* = 7.9 Hz, 1H, ArH), 7.53 (dd, *J* = 7.9, 2.0 Hz, 2H, ArH), 7.45 (dd, *J* = 7.7, 1.6 Hz, 4H, ArH), 7.35 (t, *J* = 2.0 Hz, 1H, ArH), 7.03 (m, 4H, ArH), 6.94 (td, *J* = 7.5, 1.3 Hz, 4H, ArH), 6.42 (dd, *J* = 8.2, 1.3 Hz, 4H, ArH), 1.67 (s, 12H, $-CH_3$). $^{13}$C NMR (100 MHz, Chloroform-*d*) δ 143.99, 140.72, 134.58, 132.95, 131.64, 130.25, 126.51, 125.37, 120.87, 114.00,

36.04, 31.20. HRMS (EI) calcd. for $C_{36}H_{32}N_2$ $[M+H]^+$: 493.26; Found: 493.30. Anal. calcd. for $C_{36}H_{32}N_2$: C, 87.77; H, 6.55; N, 5.69. Found: C, 87.42; H, 6.41; N, 5.91.

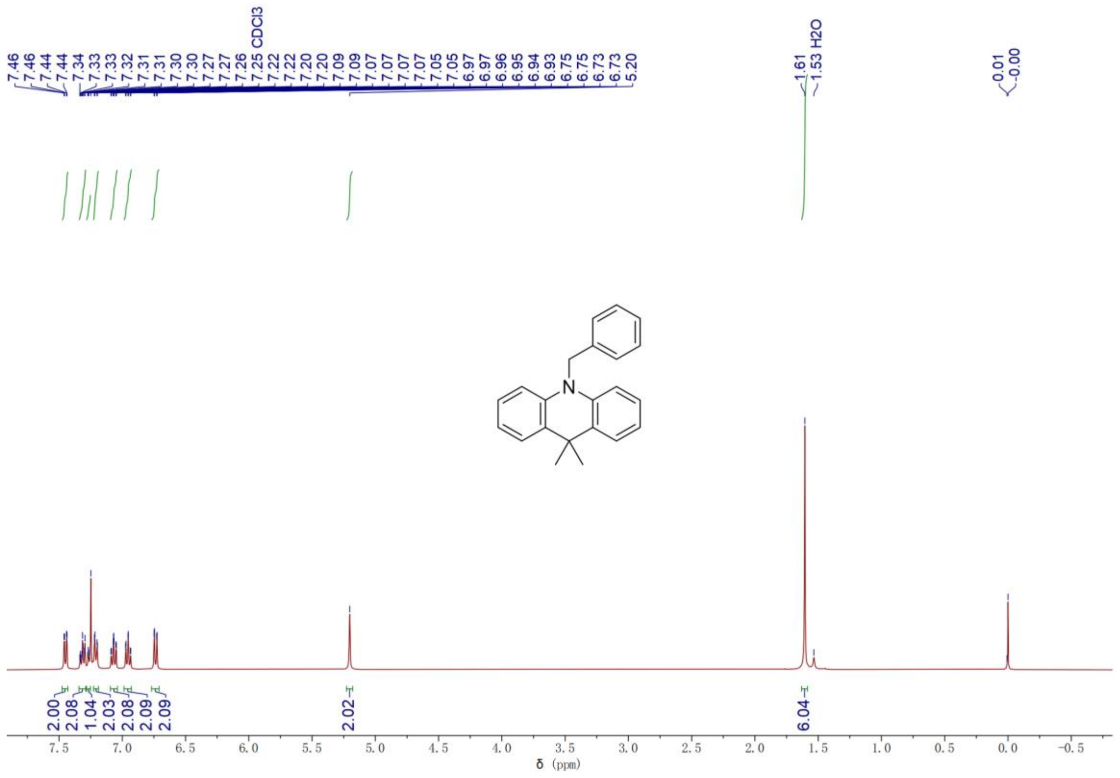

**Figure S3.** $^{1}H$ NMR spectrum of B-$CH_2$-Ac.

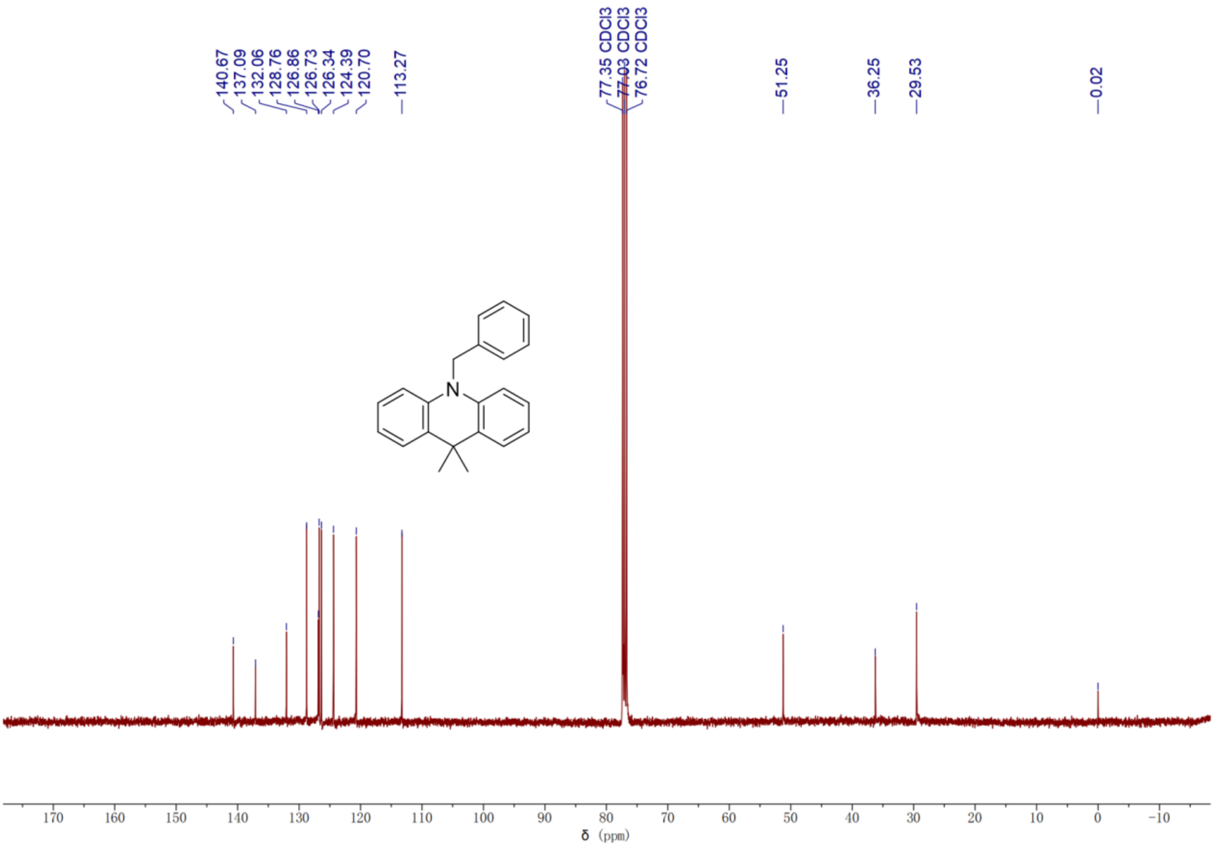

**Figure S4.** $^{13}C$ NMR spectrum of B-$CH_2$-Ac.

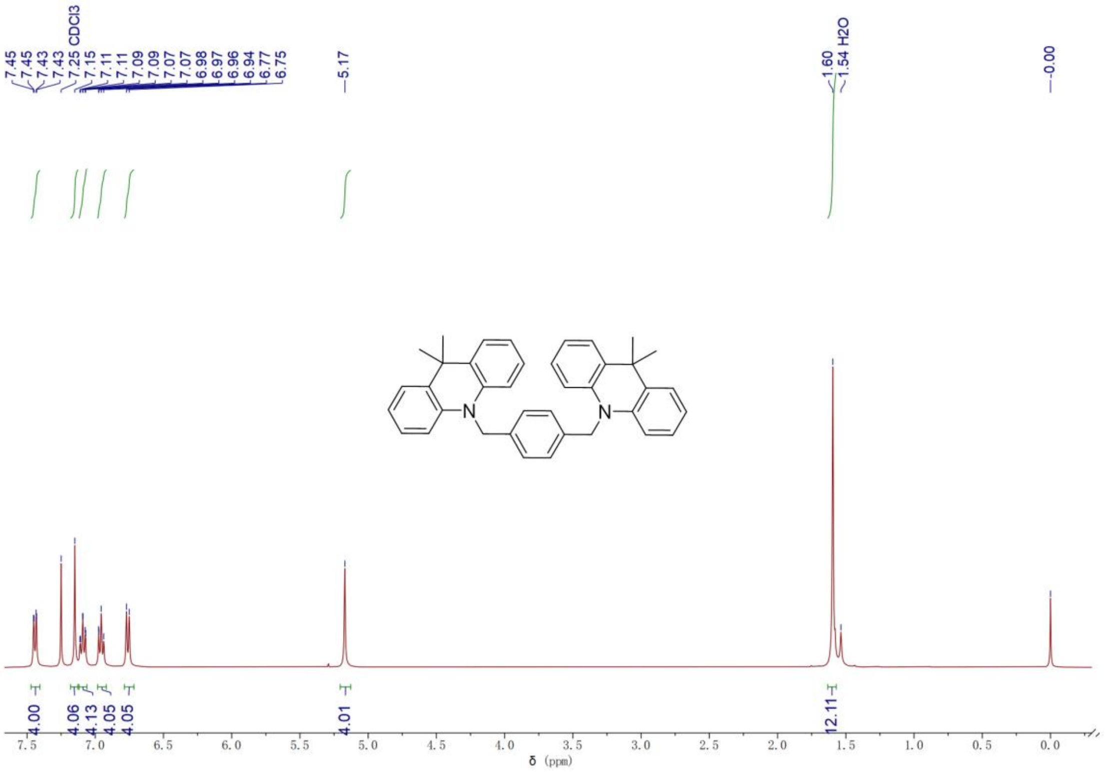


**Figure S5.** $^{1}H$ NMR spectrum of *p*-$CH_2$-DAc.

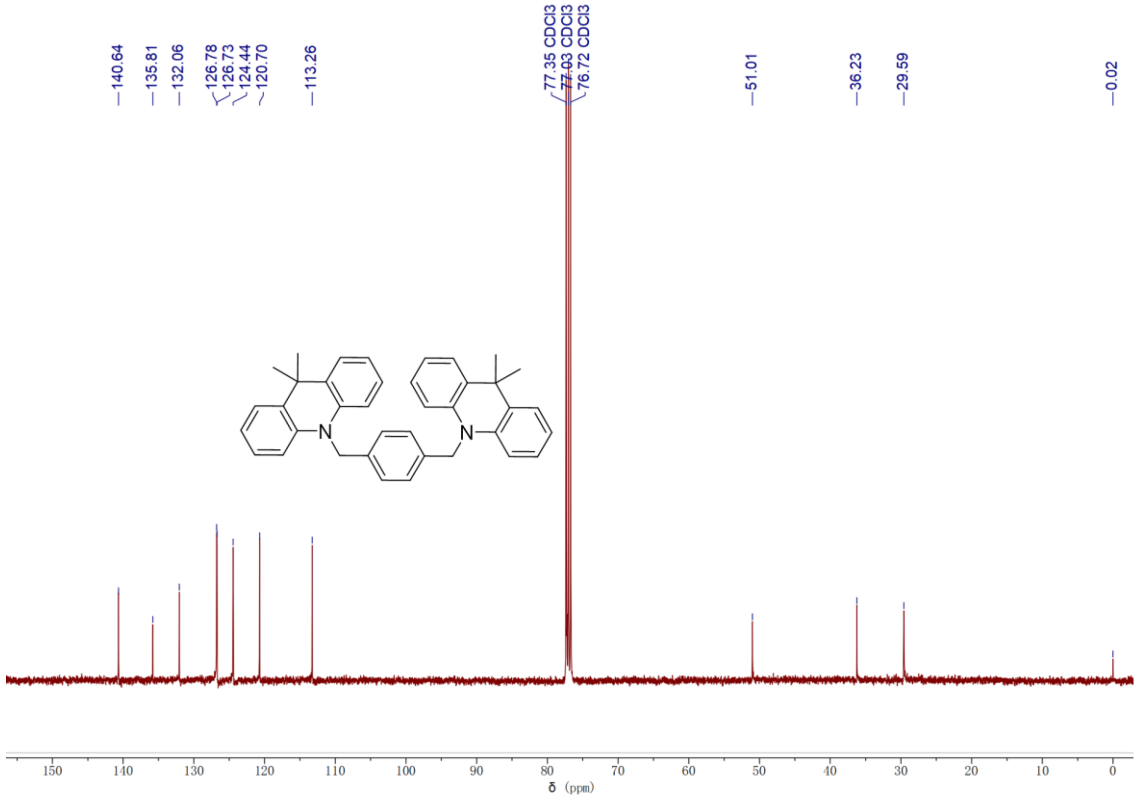


**Figure S6.** $^{13}C$ NMR spectrum of *p*-$CH_2$-DAc.

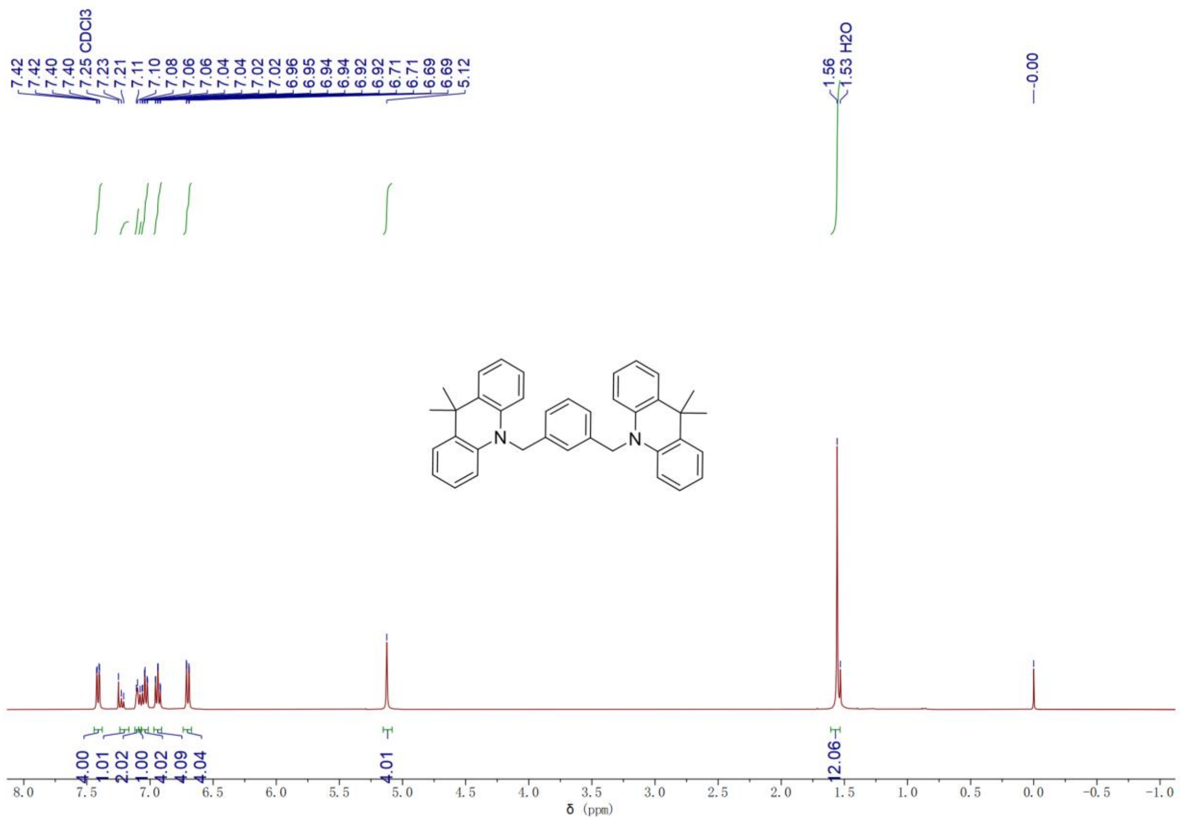

**Figure S7.** $^{1}$H NMR spectrum of *m*-$CH_2$-DAc.

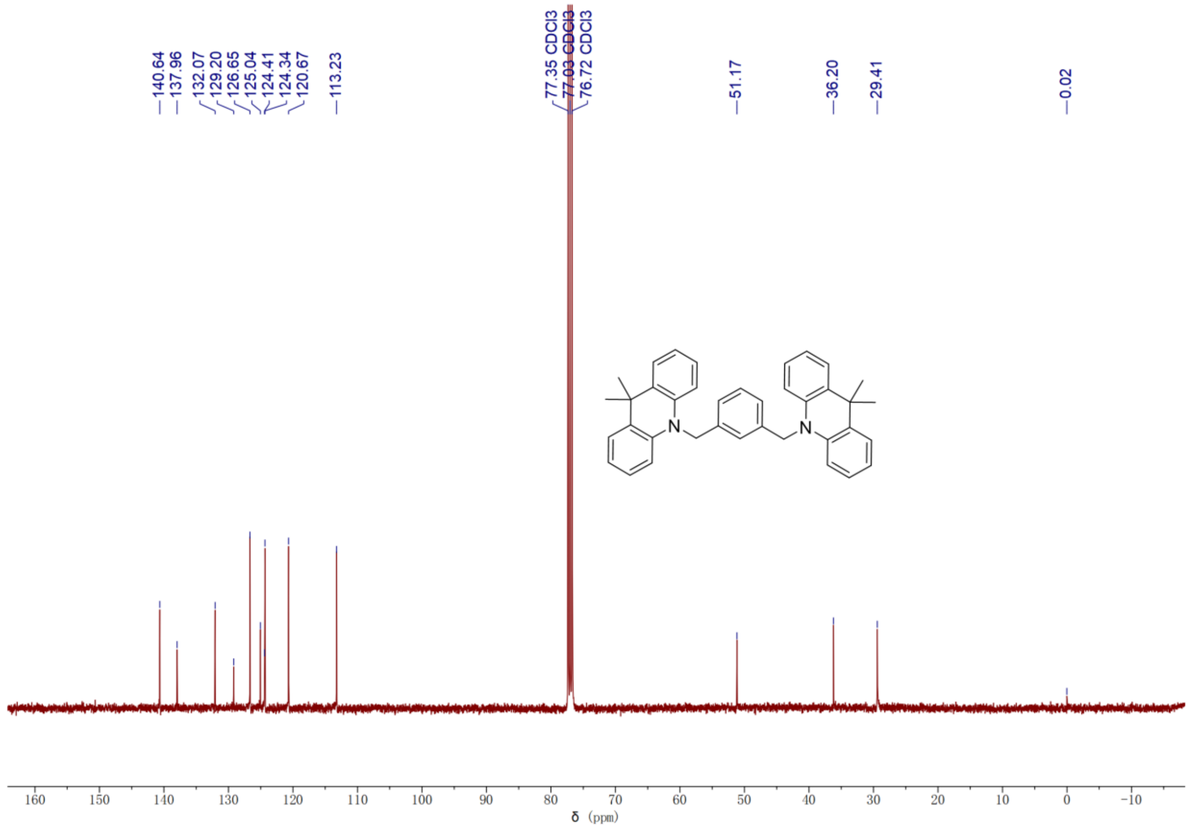

**Figure S8.** $^{13}$C NMR spectrum of *m*-$CH_2$-DAc.

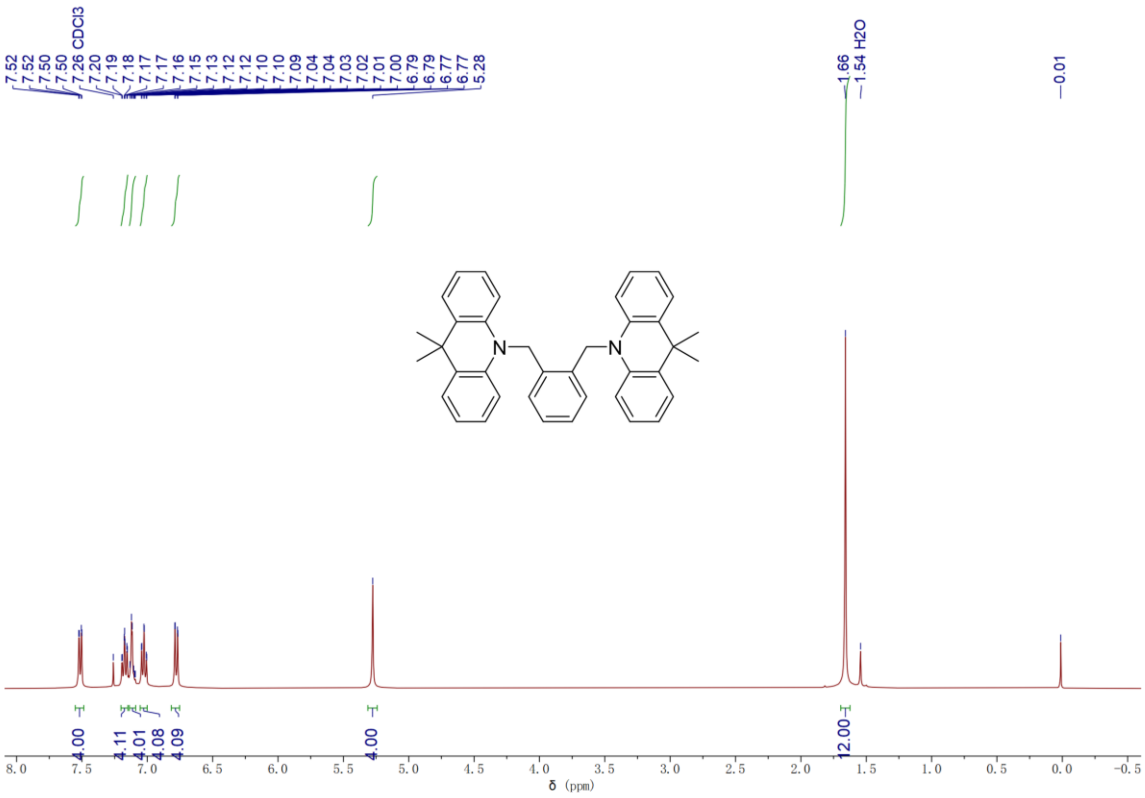


**Figure S9.** $^{1}$H NMR spectrum of *o*-$CH_2$-DAc.

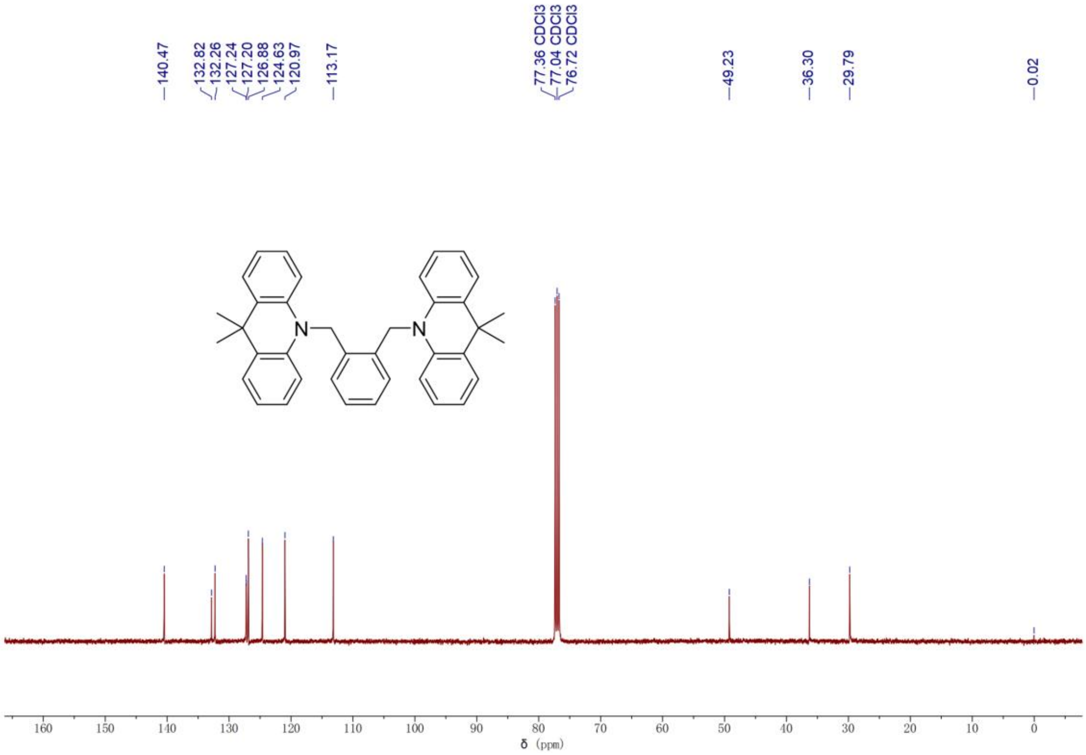


**Figure S10.** $^{13}$C NMR spectrum of *o*-$CH_2$-DAc.

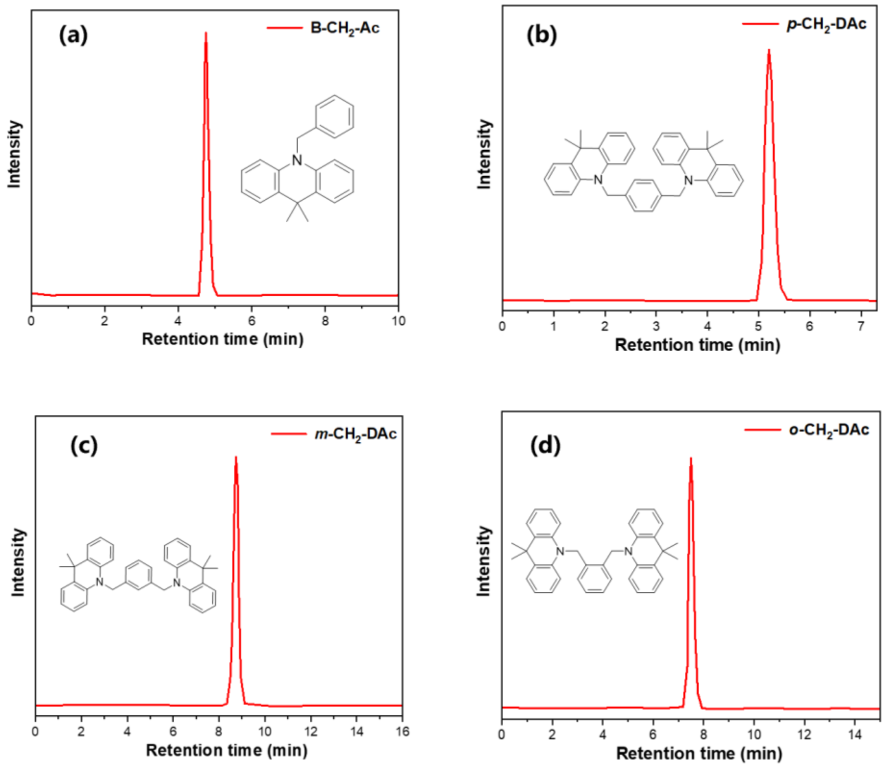


**Figure S11.** High performance liquid chromatogram (HPLC) spectra of (a) B-$CH_2$-Ac at crystal state, (b) *p*-$CH_2$-DAc at crystal state, (c) *m*-$CH_2$-DAc at crystal state and (d) *o*-$CH_2$-DAc at crystal state. (Conducted on Waters 600)

**Table S1.** Data of B-$CH_2$-Ac, *p*-$CH_2$-DAc, *m*-$CH_2$-DAc, and *o*-$CH_2$-DAc crystals.

| **Name** | **B-$CH_2$-Ac** | ***p*-$CH_2$-DAc** | ***m*-$CH_2$-DAc** | ***o*-$CH_2$-DAc** |
|---|---|---|---|---|
| **Formula** | $C_{22}H_{21}N$ | $C_{38}H_{36}N_2$ | $C_{38}H_{36}N_2$ | $C_{38}H_{36}N_2$ |
| **Crystal system** | monoclinic | monoclinic | monoclinic | monoclinic |
| **Space Group** | $I\ 2/a$ | $P\ 2_1/n$ | $C\ 2/c$ | $P\ 2_1/n$ |
| **Cell Lengths (Å)** | 18.9280(6)<br>7.9777(2)<br>22.6848(6) | 15.1151(15)<br>5.9262(5)<br>17.4841(17) | 14.3699(4)<br>11.4860(2)<br>17.5617(4) | 7.5745(2)<br>22.8173(8)<br>16.9342(7) |
| **Cell Angles (°)** | 90<br>96.854(3)<br>90 | 90<br>113.344(12)<br>90 | 90.0<br>101.480(2)<br>90.0 | 90.0<br>90.144(3)<br>90.0 |
| **Cell Volume ($Å^3$)** | 3400.97(17) | 1437.9(3) | 2840.62(12) | 2926.7(2) |
| **Z** | 8 | 2 | 8 | 4 |
| **Density ($g/cm^3$)** | 1.169 | 1.203 | 1.218 | 1.182 |
| **CCDC** | 2240597 | 2240065 | 2240069 | 2240084 |

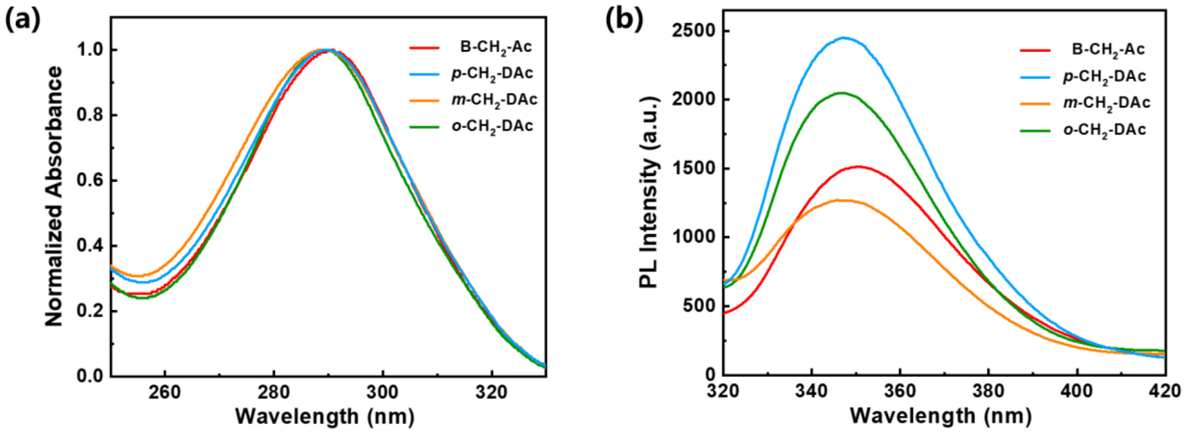


**Figure S12.** (a) UV-Vis absorption spectra of B-$CH_2$-Ac, *p*-$CH_2$-DAc, *m*-$CH_2$-DAc and *o*-$CH_2$-DAc in DCM solution with the concentration of 10 $\mu$M. (b) PL spectra of B-$CH_2$-Ac, *p*-$CH_2$-DAc, *m*-$CH_2$-DAc and *o*-$CH_2$-DAc in DCM solution with the concentration of 10 $\mu$M.

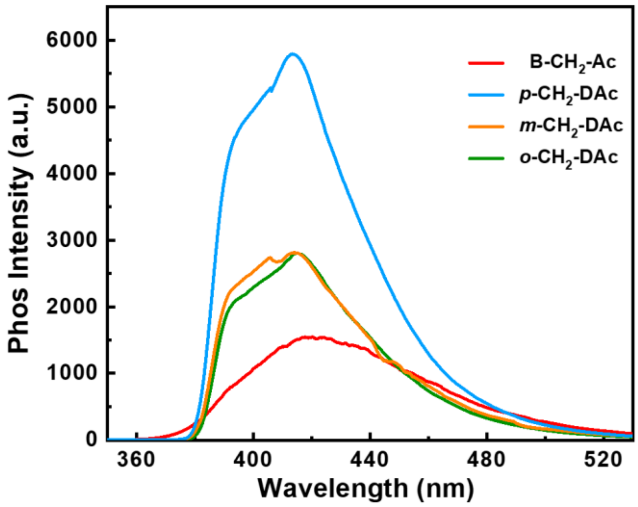


**Figure S13.** Phosphorescence spectra of organic luminogens in dilute 2-Methylfuran solutions (10 $\mu$M) at 77 K.

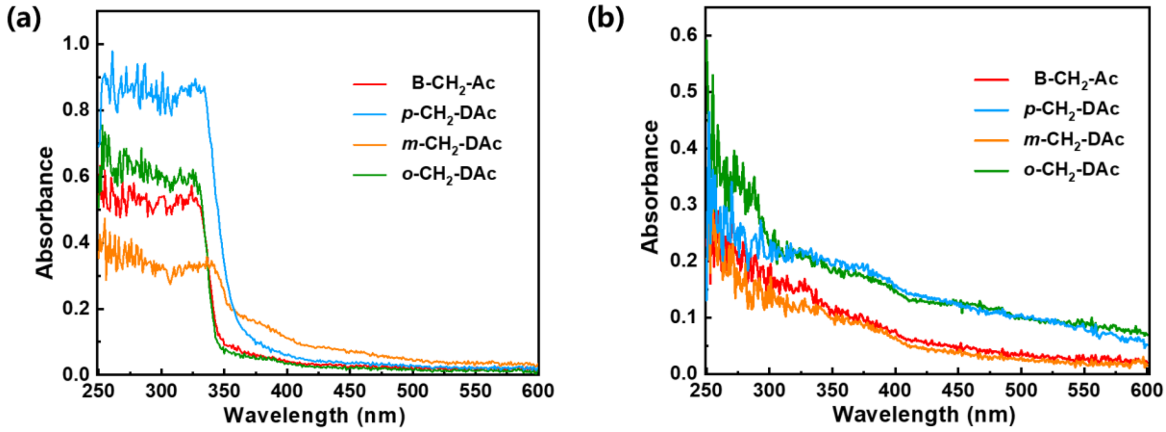


**Figure S14.** (a) Absorption spectra of B-$CH_2$-Ac, *p*-$CH_2$-DAc, *m*-$CH_2$-DAc and *o*-$CH_2$-DAc at crystal state. (b) Absorption spectra of B-$CH_2$-Ac, *p*-$CH_2$-DAc, *m*-$CH_2$-DAc and *o*-$CH_2$-DAc at solidified state.

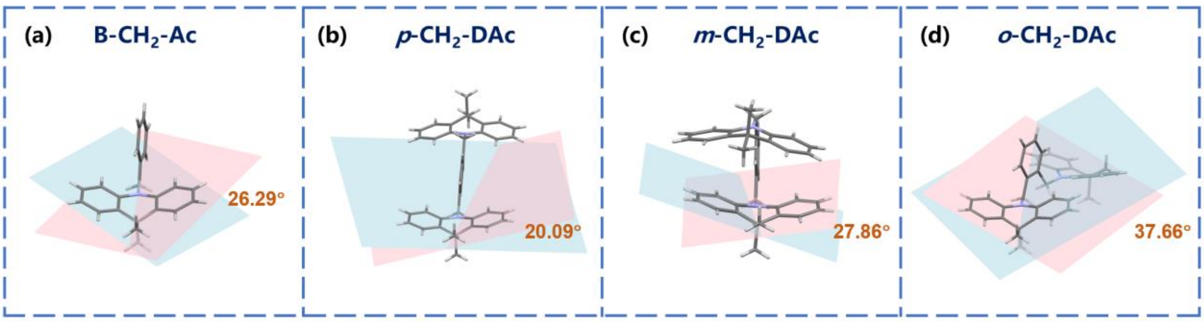


**Figure S15.** The molecular conformations of (a) B-$CH_2$-Ac, (b) *p*-$CH_2$-DAc, (c) *m*-$CH_2$-DAc and (d) *o*-$CH_2$-DAc in single crystals.

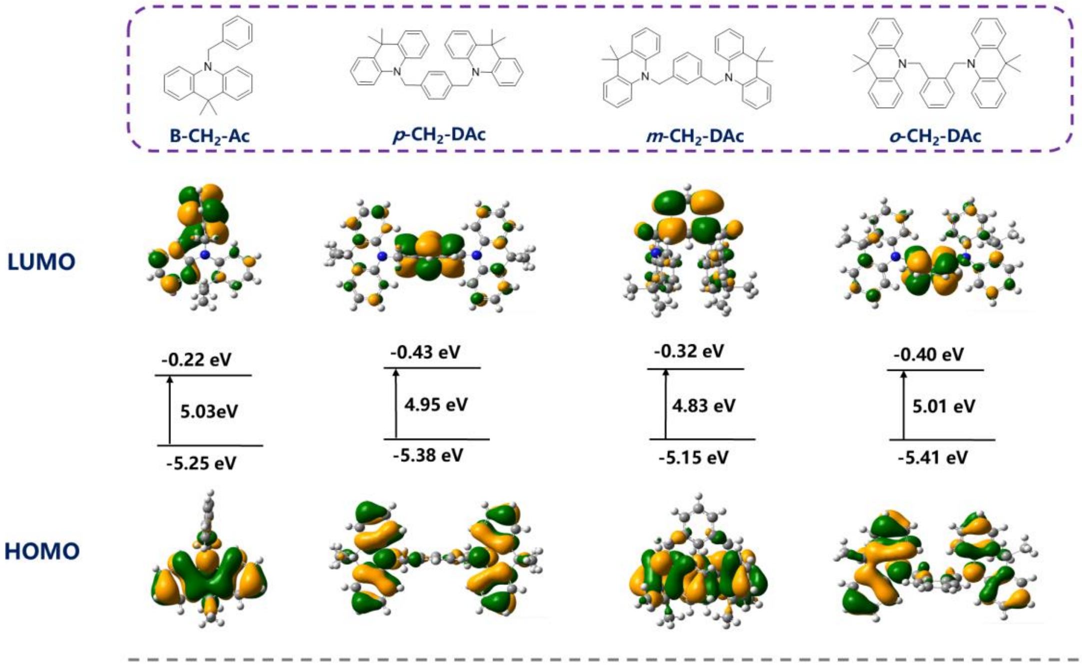


**Figure S16.** HOMOs, LUMOs and energy gaps of B-$CH_2$-Ac, *p*-$CH_2$-DAc, *m*-$CH_2$-DAc, and *o*-$CH_2$-DAc.

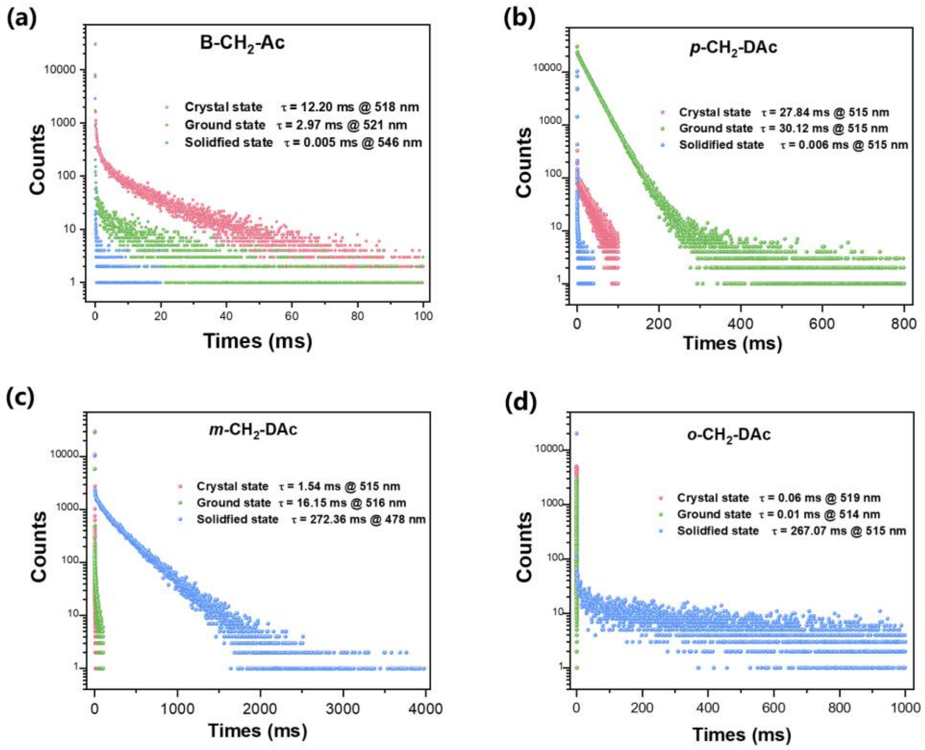


**Figure S17.** Phosphorescence decays of (a) B-$CH_2$-Ac, (b) *p*-$CH_2$-DAc, (c) *m*-$CH_2$-DAc, and (d) *o*-$CH_2$-DAc at different aggregated states.

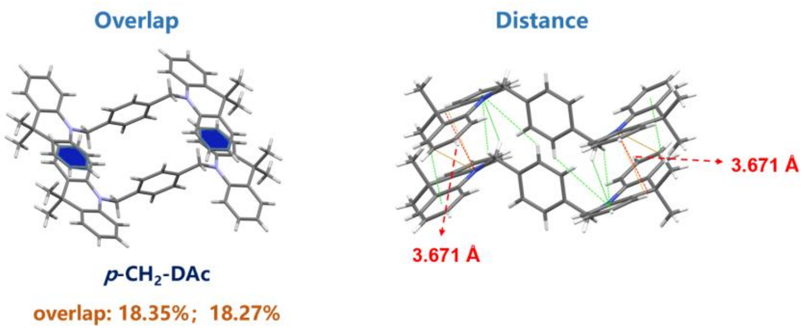


**Figure S18.** The overlaps of the involved phenyl in acridine moieties in ***p*-CH$_2$-DAc** crystal.

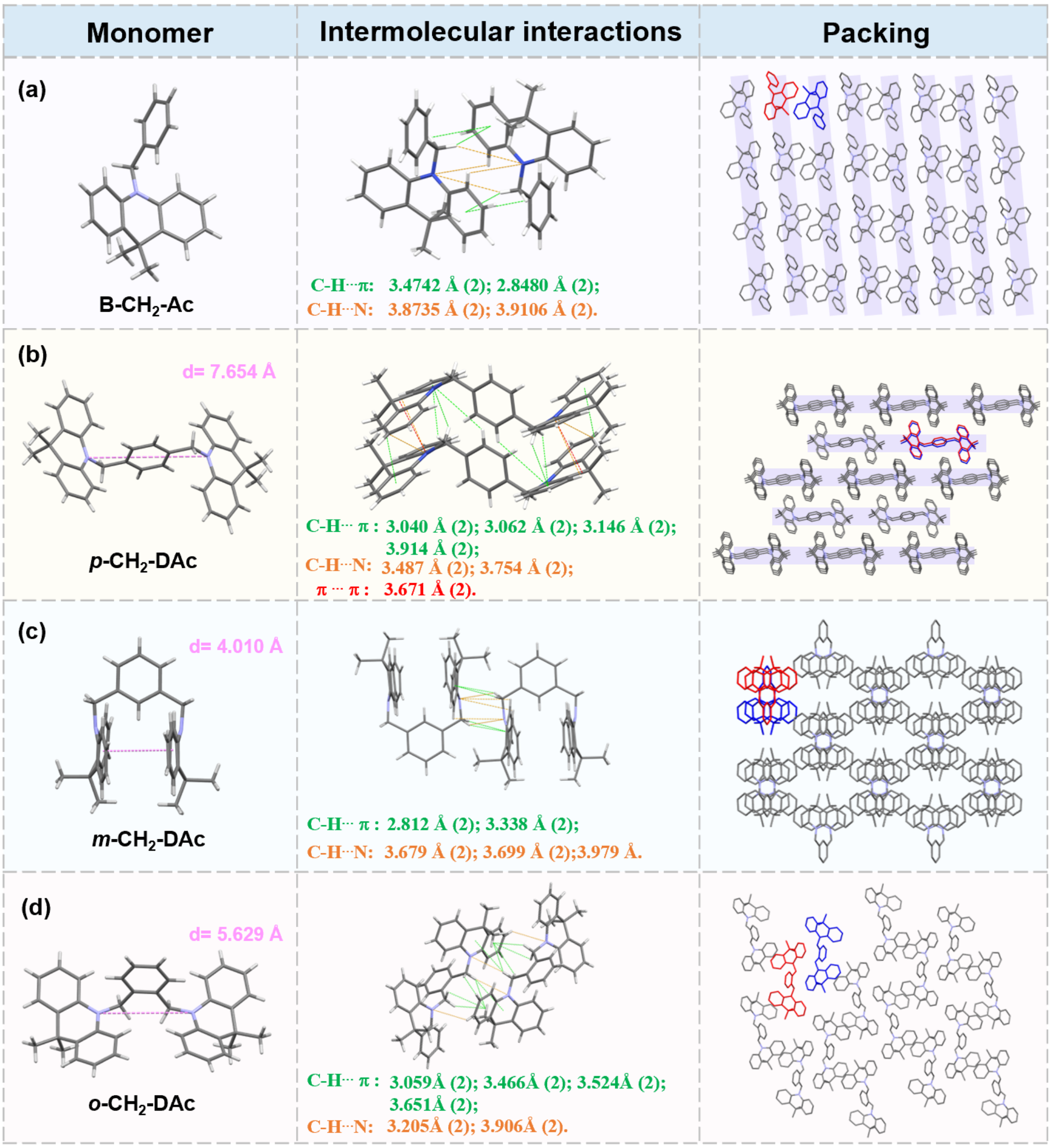


**Figure S19.** Molecular conformations, intermolecular interactions, and molecular packing in (a) B-CH$_2$-Ac, (b) *p*-CH$_2$-DAc, (c) *m*-CH$_2$-DAc and (d) *o*-CH$_2$-DAc crystals.

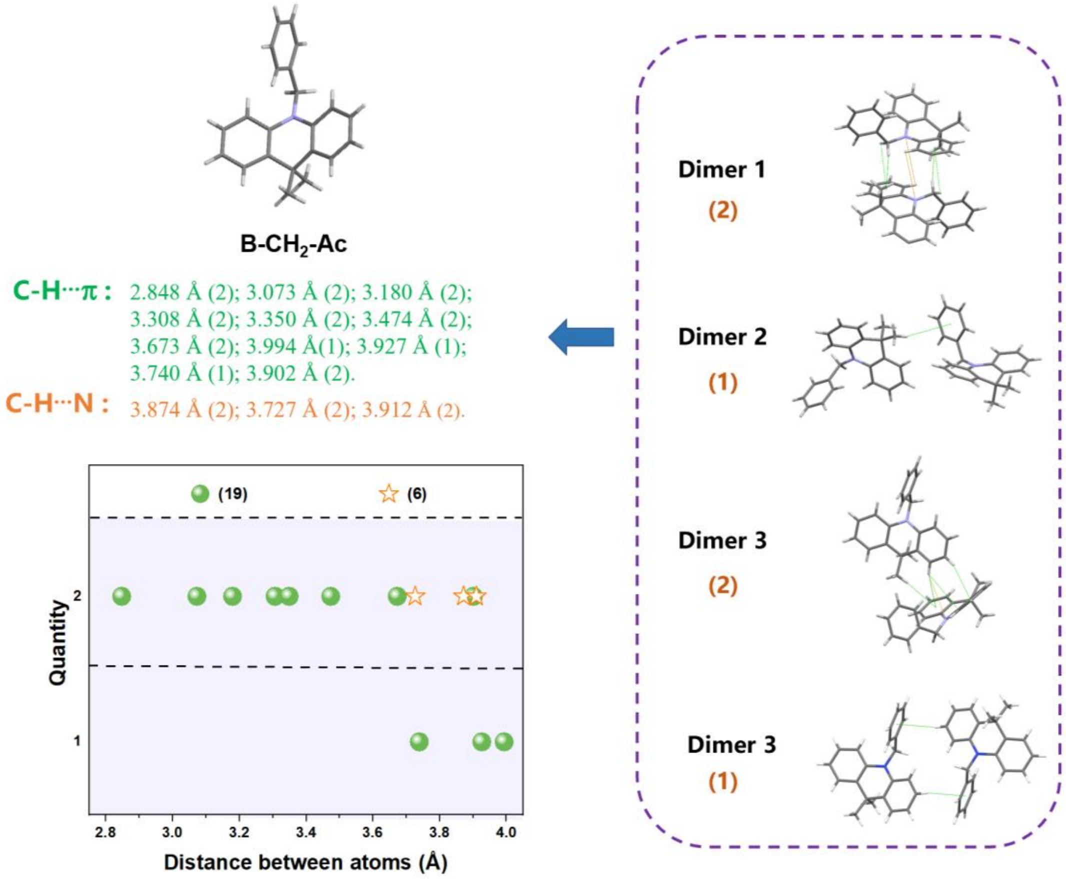


**Figure S20**. Intermolecular interactions in B-$CH_2$-Ac crystal.

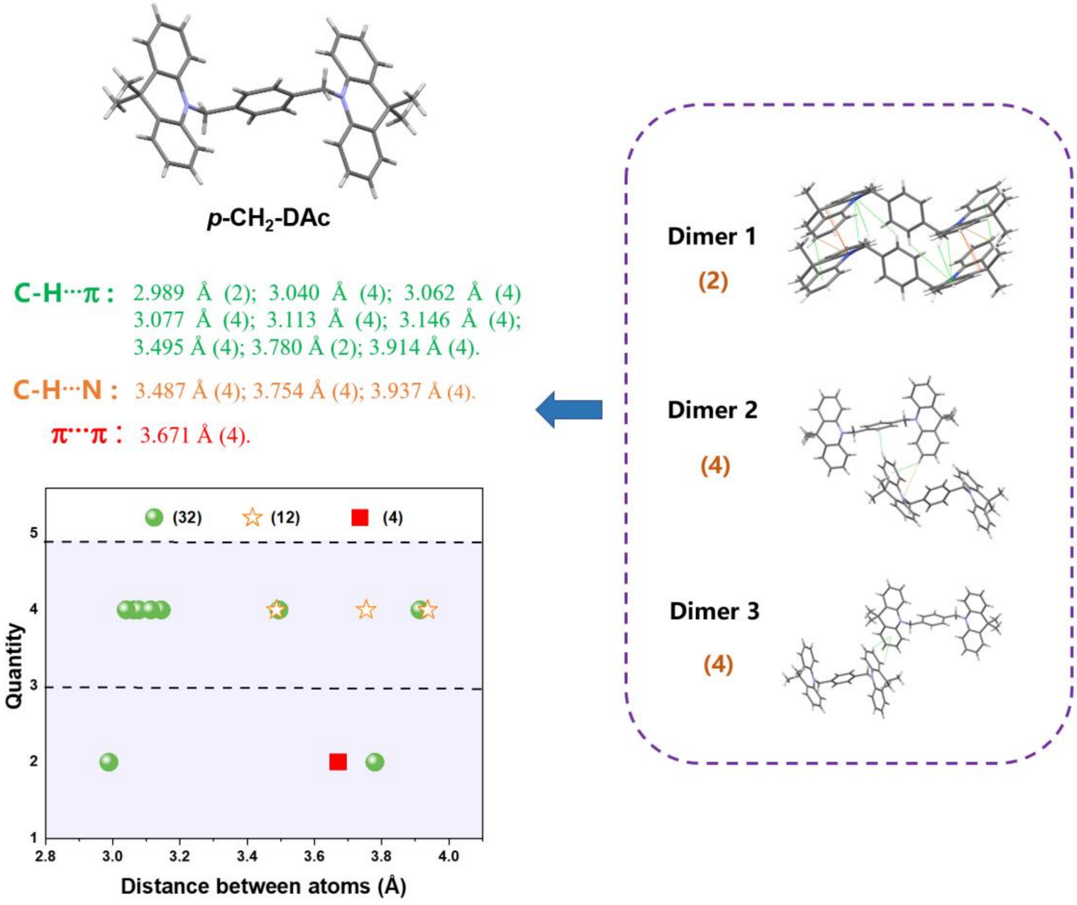


**Figure S21**. Intermolecular interactions in *p*-$CH_2$-DAc crystal.

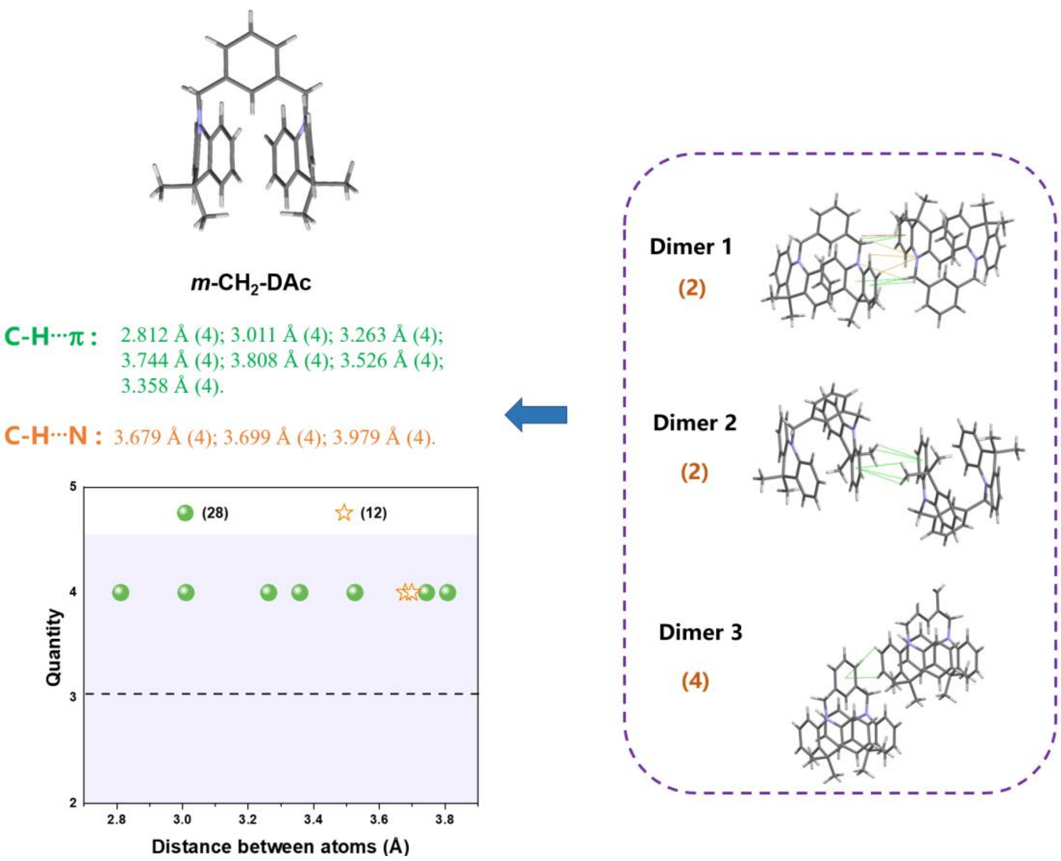


**Figure S22**. Intermolecular interactions in *m*-$CH_2$-DAc crystal.

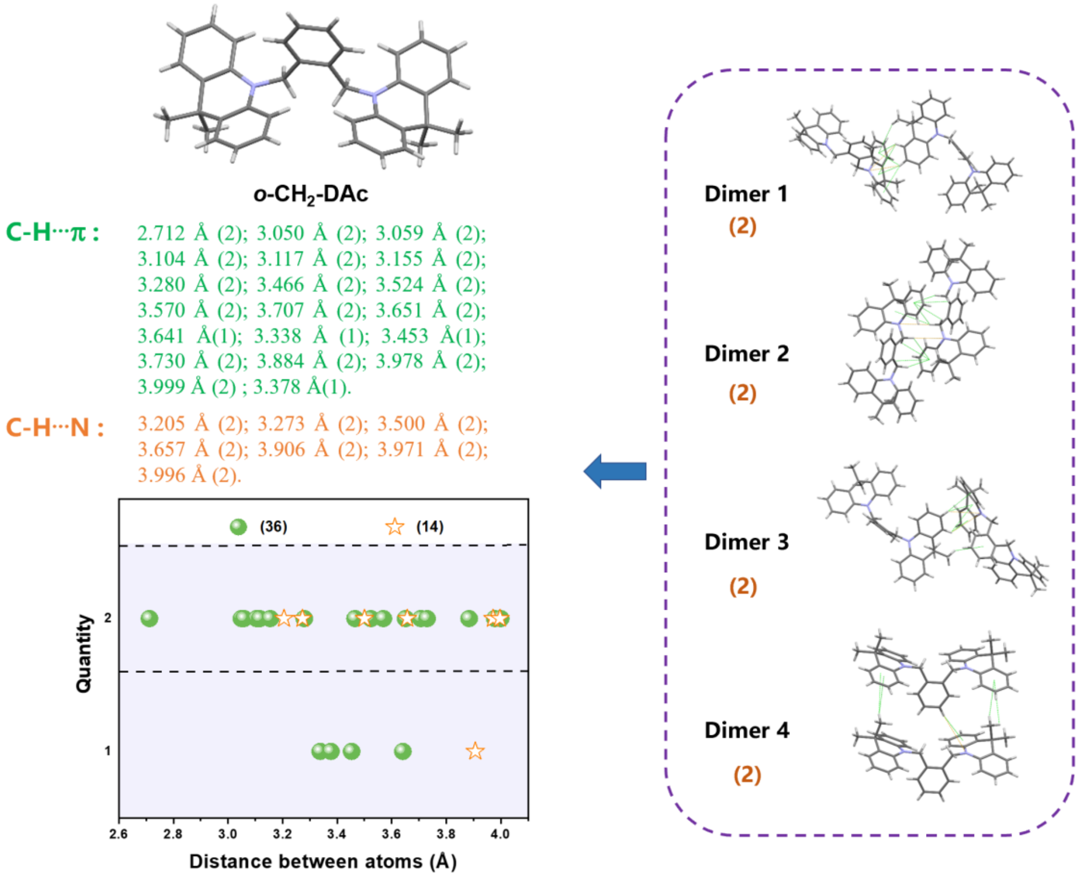


**Figure S23**. Intermolecular interactions in *o*-$CH_2$-DAc crystal.

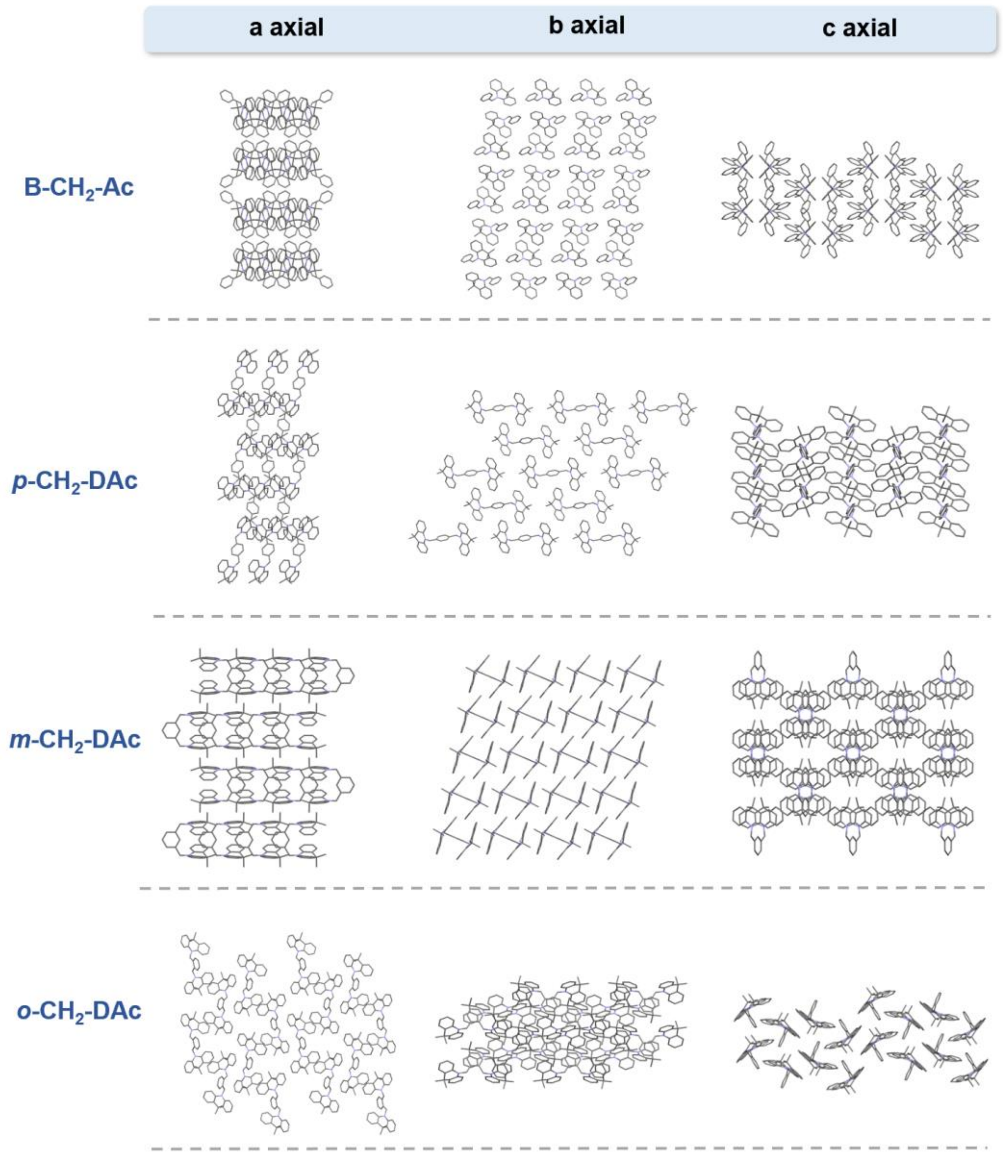


**Figure S24.** The molecular packing of B-$CH_2$-Ac, *p*-$CH_2$-DAc, *m*-$CH_2$-DAc, and *o*-$CH_2$-DAc crystals, observed from the *a*, *b*, and *c* axial directions.

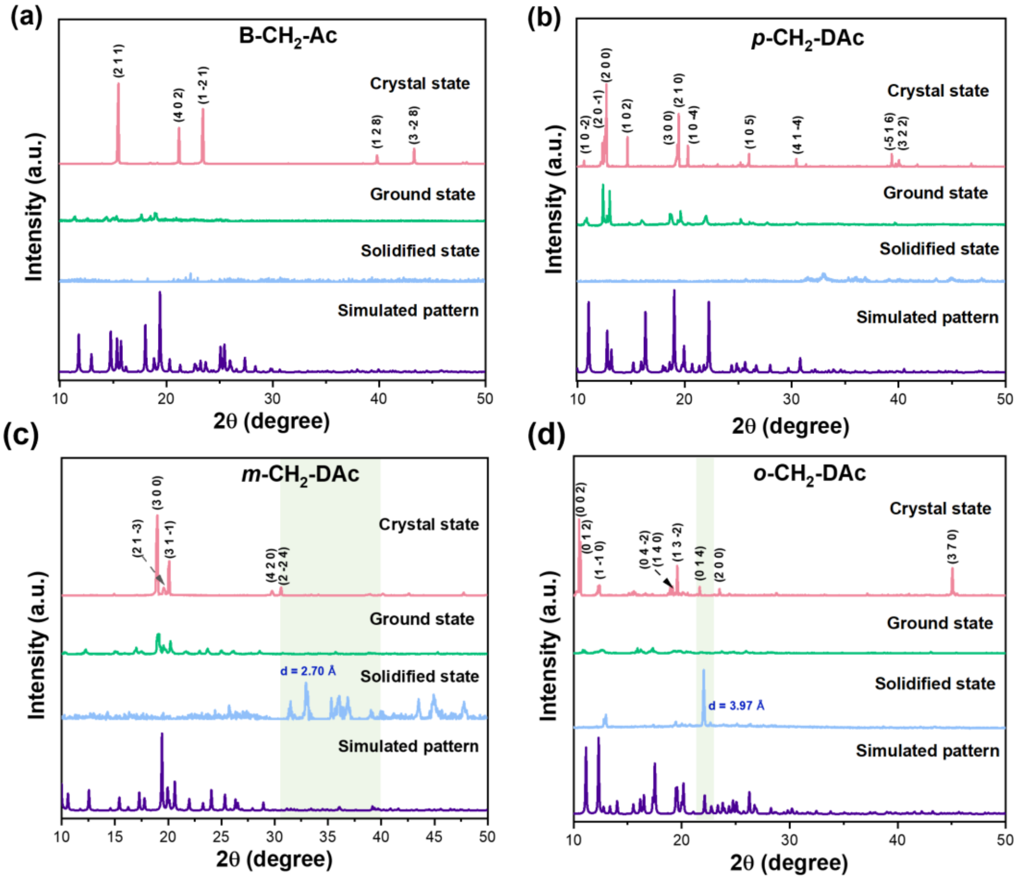


**Figure S25.** Powder X-ray diffraction (PXRD) patterns for (a) B-$CH_2$-Ac, (b) *p*-$CH_2$-DAc, (c) *m*-$CH_2$-DAc and (d) *o*-$CH_2$-DAc (The simulated XRD pattern calculated from single-crystal X-ray data with Mercury 2022.2.0) at different states, including crystal states, ground states and solidified states.

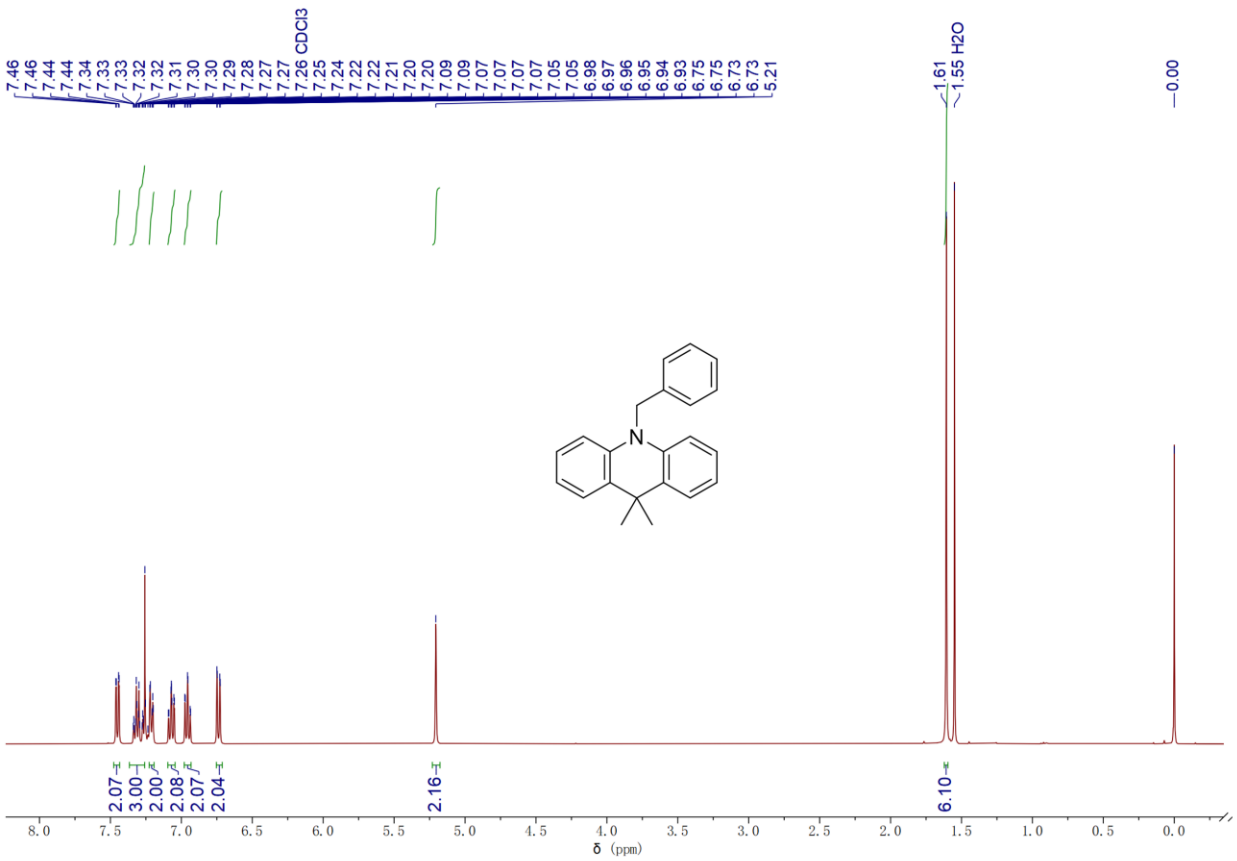

**Figure S26.** $^{1}H$ NMR spectrum of B-$CH_2$-Ac after thermal annealing.

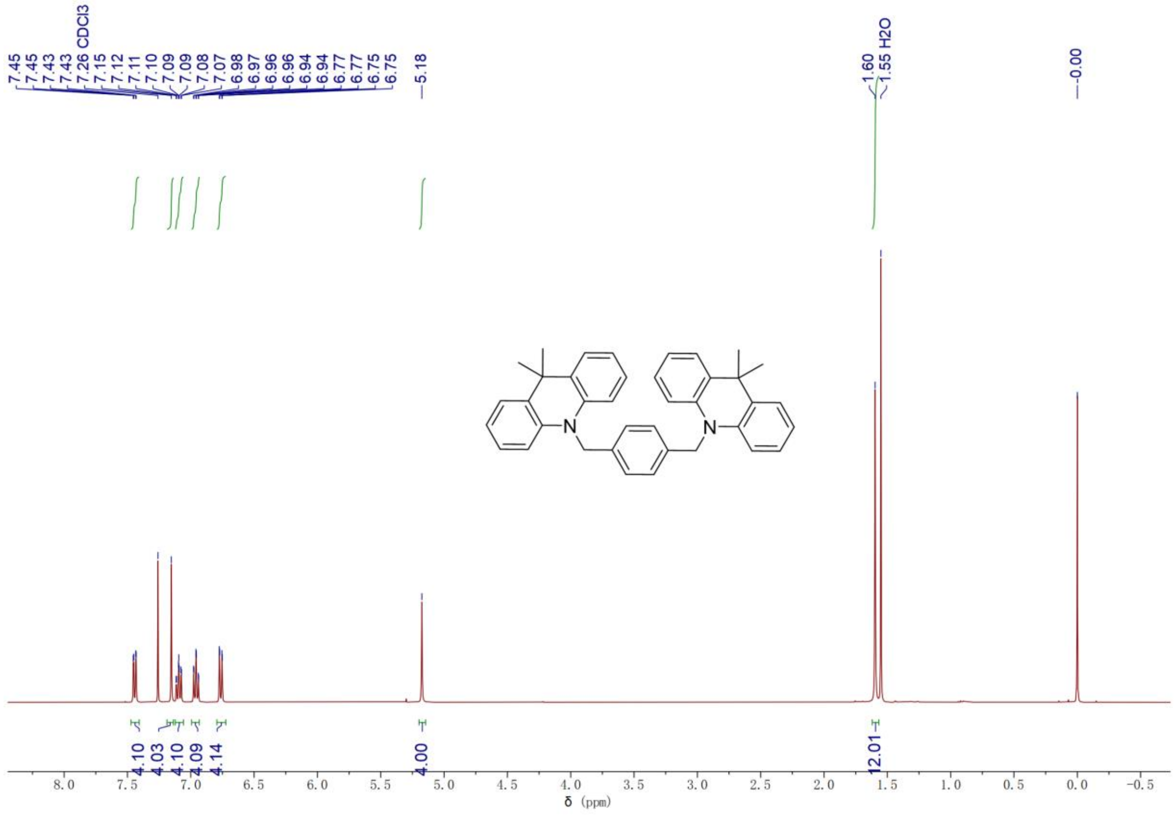

**Figure S27.** $^{1}H$ NMR spectrum of *p*-$CH_2$-DAc after thermal annealing.

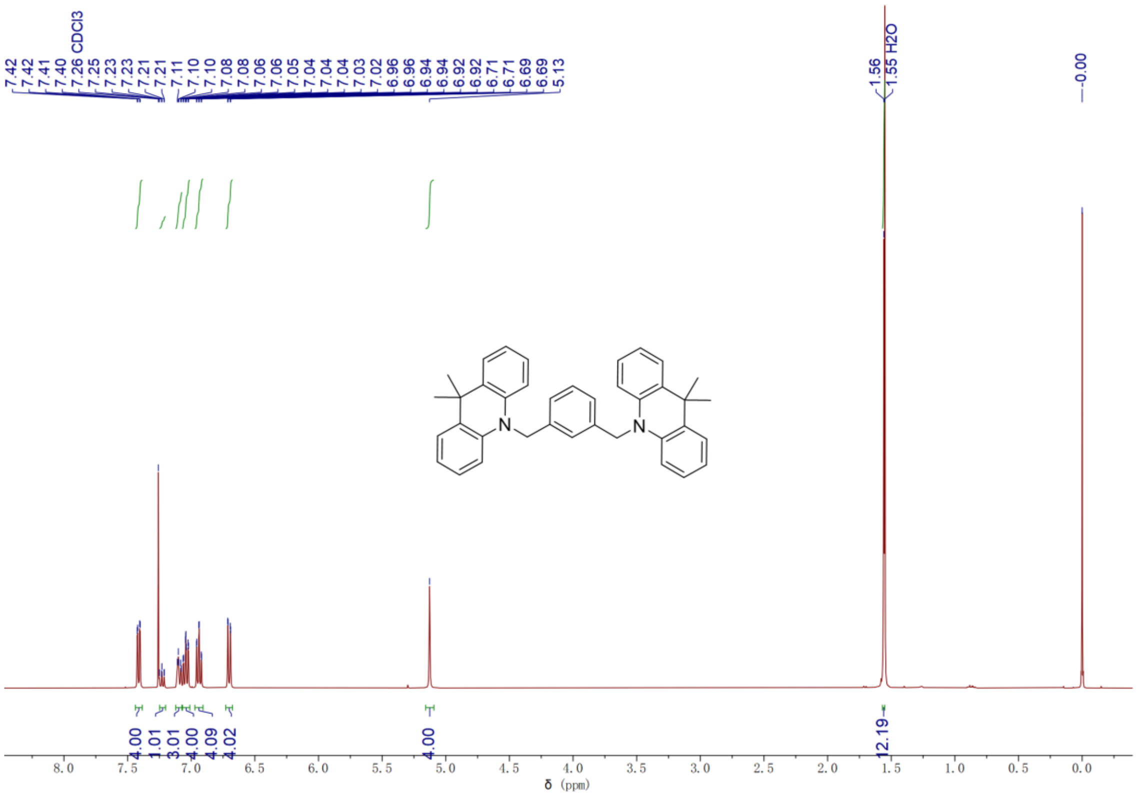

**Figure S28.** $^1$H NMR spectrum of *m*-$CH_2$-DAc after thermal annealing.

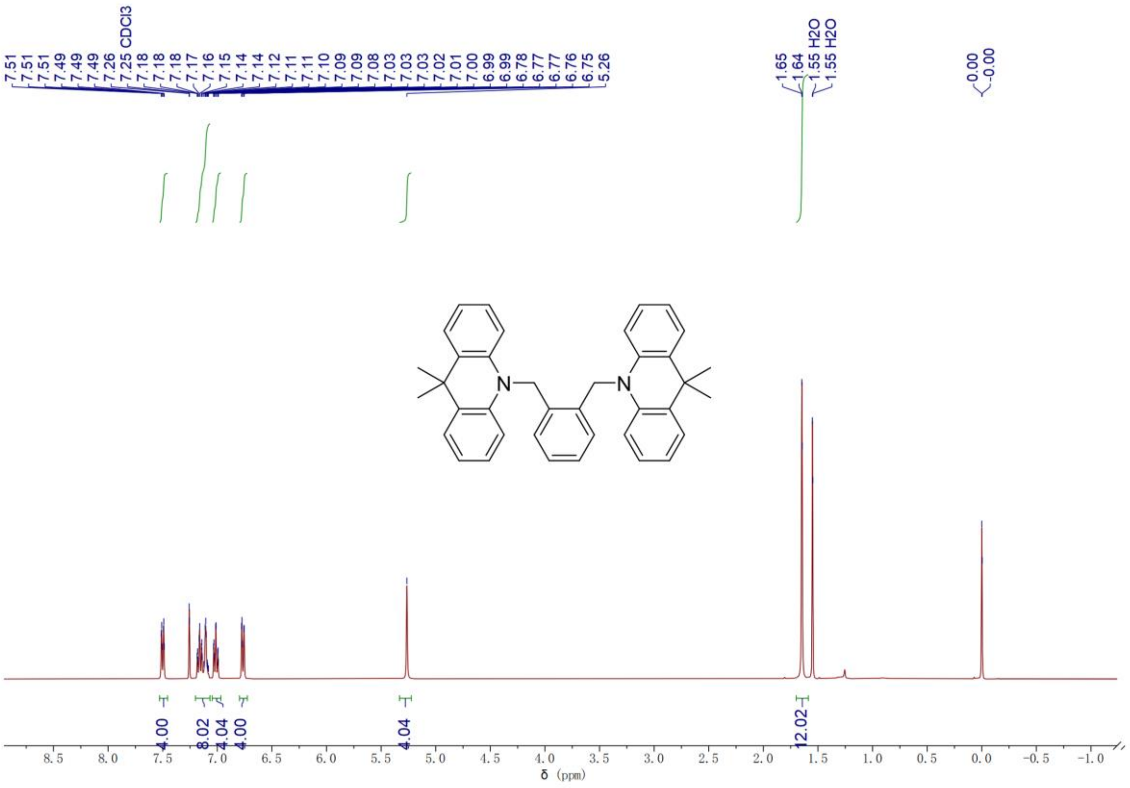

**Figure S29.** $^1$H NMR spectrum of *o*-$CH_2$-DAc after thermal annealing.

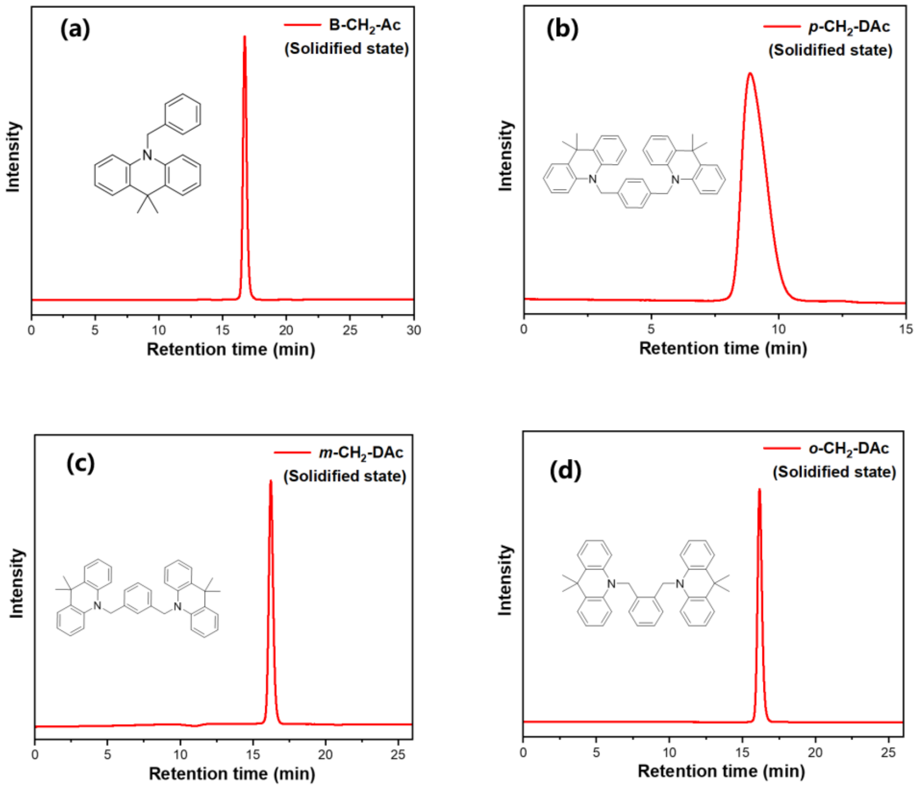


**Figure S30.** High performance liquid chromatogram (HPLC) spectra of (a) B-CH$_2$-Ac, (b) *p*-CH$_2$-DAc, (c) *m*-CH$_2$-DAc and (d) *o*-CH$_2$-DAc at solidified states (Conducted on LaboACE LC-500).

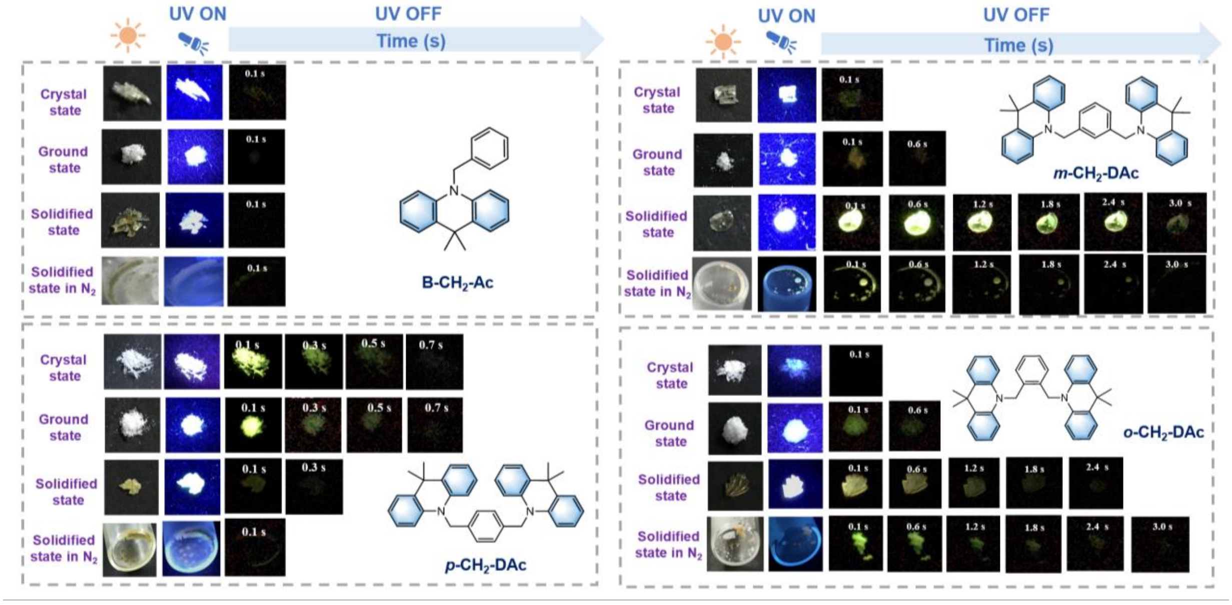

**Figure S31.** Photographs of B-$CH_2$-Ac, *p*-$CH_2$-DAc, *m*-$CH_2$-DAc and *o*-$CH_2$-DAc at crystal state, ground state, and solidified state under nitrogen atmosphere taken before and after UV irradiation (365 nm).

**The detailed procedures for thermal annealing**

The different thermal annealing processes have been designed by the adjustable heating and cooling rates, together with the different standing time at melting points, which can be divided into three parts:

1. **Modulation of heating rates**

The heating rates were modulated by the different initial temperature of hot plates, which varied from 25 ℃, to 100 ℃, to 150℃, then to 200 ℃. Once the temperature increased to the melting points (220 ℃), the standing time at this temperature is 15 s for all the samples, then they were cooled to room temperature naturally by turning off the heater. It could be seen that the most persistent RTP property is achieved from the highest initial temperature (200 ℃) of hot plate, indicating that the rapid heating process is beneficial to the RTP property upon thermal annealing process.

2. **Modulation of standing time at melting point**

Once the samples were melted by the increased temperature of hot plates from 25 $^{o}$C to 220 $^{o}$C, the standing time at 220 $^{o}$C varied from 0 s, to 15 s, then to 30 s in different thermal annealing processes, then they were cooled to room temperature naturally by turning off the heater. It could be seen that the brighter afterglow at solidified state can be achieved with the standing time of 15 s.

3. **Modulation of cooling rates**

Based on the above experiments, the heating rates and standing time at melting points have been optimized. Accordingly, the cooling rates have been modulated by placing samples on cold packs, in air and turning off the hot plates, respectively. It could be seen that a rapid cooling process with the aid of cold packs can result in the much improved RTP property after thermal annealing.

**Optimized procedures for thermal annealing process**

Once the samples were melted by the step-heating method, heating from 25 ℃ to 200 ℃ instantaneously by placing the samples on the hot plates with the initial temperature of 200 ℃, then heating from 200 ℃ to 220 ℃ within 80 s. The standing time at melting point is 15 s, then they were cooled to room temperature with the aid of a cold pack. (Video S1)

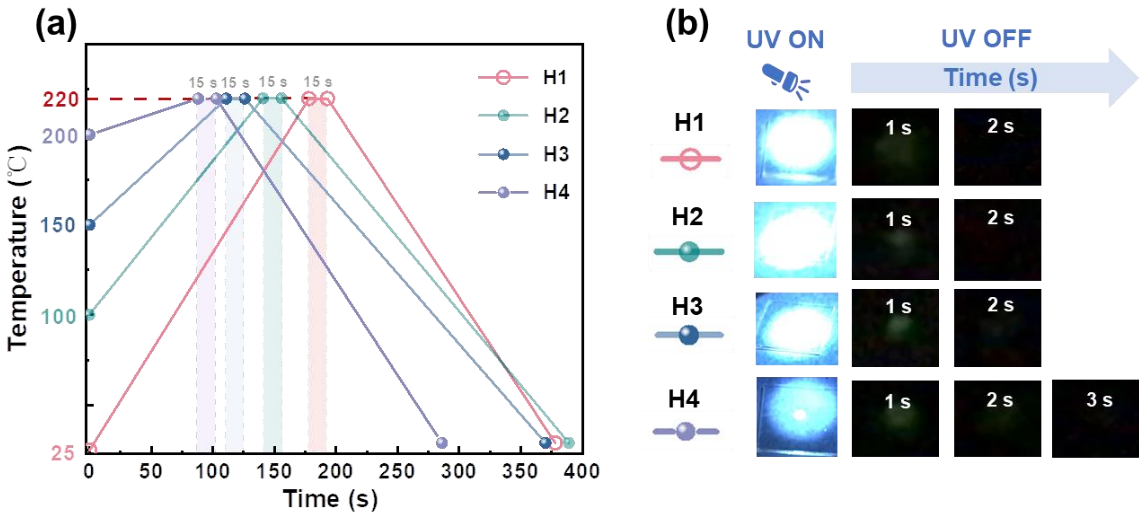


**Figure S32.** (a) Different thermal annealing processes by the variations of initial temperature of hot plates at 25 ℃, 100 ℃, 150 ℃, and 200 ℃, respectively. **H1**: Heating from 25 ℃ to 220 ℃ within 175 s; **H2:** Step-heating method, heating from 25 ℃ to 100 ℃ instantaneously by placing the samples on the hot plates with the initial temperature of 100 ℃, then heating from 100 ℃ to 220 ℃ within 150 s; **H3**: Step-heating method, heating from 25 ℃ to 150 ℃ instantaneously by placing the samples on the hot plates with the initial temperature of 150 ℃, then heating from 150 ℃ to 220 ℃ within 110 s; **H4**: Step-heating method, heating from 25 ℃ to 200 ℃ instantaneously by placing the samples on the hot plates with the initial temperature of 200 ℃, then heating from 200 ℃ to 220 ℃ within 80 s. (b) The corresponding photographs of *m*-$CH_2$-DAc at solidified states taken after UV irradiation (365 nm) under ambient conditions.

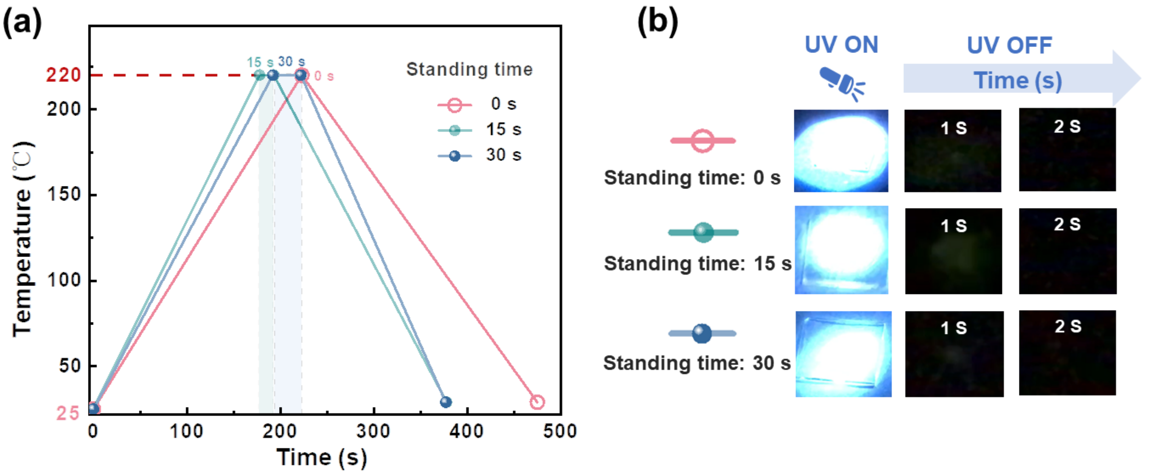


**Figure S33.** (a) Different thermal annealing processes with the varied standing time at melting points. (b) The corresponding photographs of *m*-$CH_2$-DAc at solidified states taken after UV irradiation (365 nm) under ambient conditions.

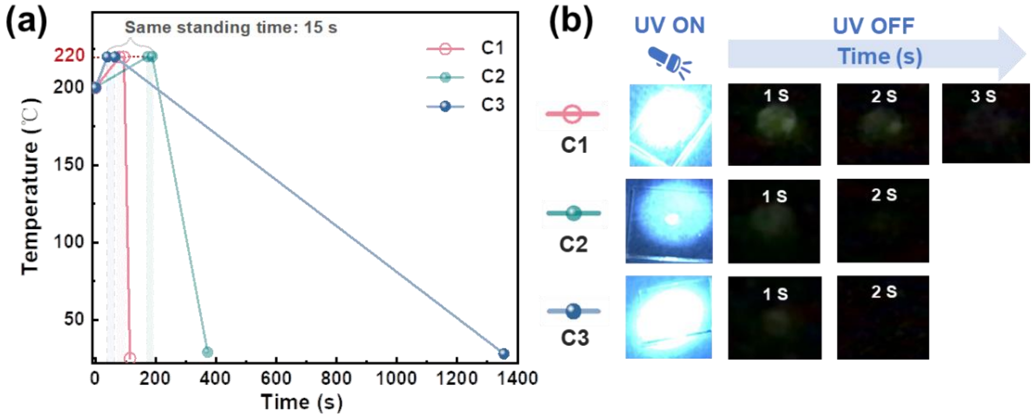


**Figure S34.** (a) Different thermal annealing process with changing the cooling rates by placing samples on cold packs (C1), in air (C2) and turning off the hot plates (C3), respectively. (b) The corresponding photographs of *m*-$CH_2$-DAc at solidified states taken after UV irradiation (365 nm) under ambient conditions.

**Table S2.** Data of *m*-$CH_2$-DAc and *o*-$CH_2$-DAc at the solidified state.

| **Name** | ***m*-$CH_2$-DAc** | ***o*-$CH_2$-DAc** |
|---|---|---|
| **Formula** | $C_{38}H_{36}N_2$ | $C_{38}H_{36}N_2$ |
| **Crystal system** | monoclinic | monoclinic |
| **Space Group** | *C* 2/*c* | *P* $2_1$/*n* |
| **Cell Lengths (Å)** | 14.3811(5)<br>11.5091(4)<br>17.6004(6) | 7.460(3)<br>17.361(11)<br>22.455(7) |
| **Cell Angles (°)** | 90.0<br>101.502(3)<br>90.0 | 90.0<br>90.0<br>90.0 |
| **Cell Volume ($Å^3$)** | 2854.60 (18) | 2908 (2) |
| **Z** | 8 | 16 |
| **Density ($g/cm^3$)** | 1.210 | 1.189 |
| **CCDC** | 2251686 | 2251688 |

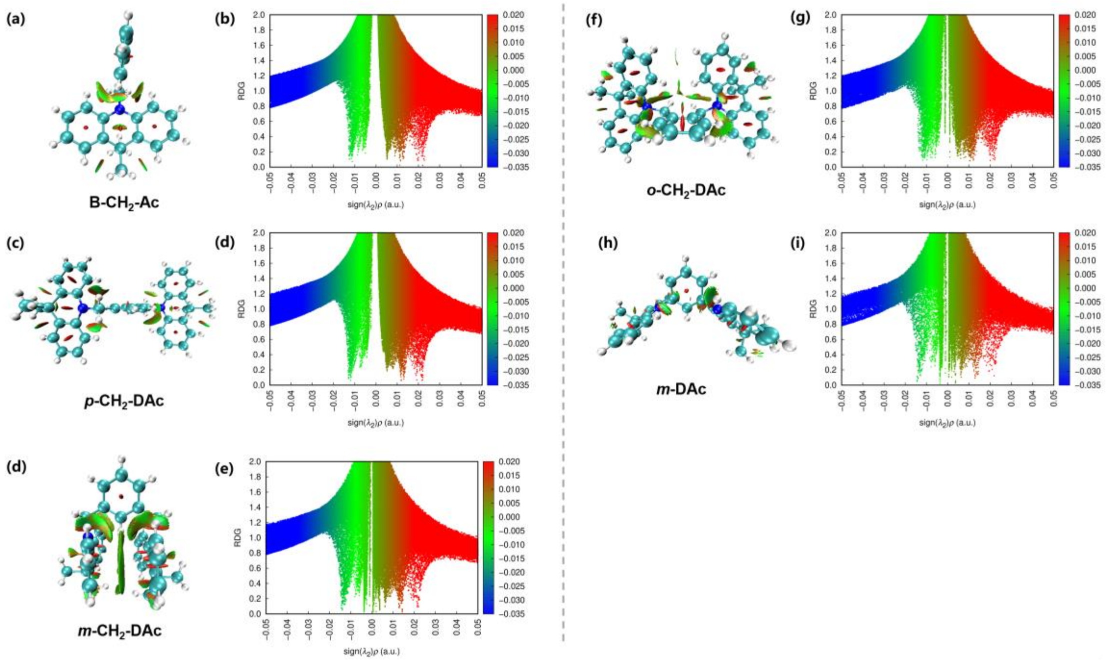


**Figure S35.** Gradient isosurfaces for organic luminogens of (a) B-$CH_2$-Ac, (c) *p*-$CH_2$-DAc, (d) *m*-$CH_2$-DAc, (f) *o*-$CH_2$-DAc, (h) *m*-DAc crystals; The scatter diagram for RDG versus sign($\lambda_2$)$\rho$ of organic luminogens of (b) B-$CH_2$-Ac, (d) *p*-$CH_2$-DAc, (e) *m*-$CH_2$-DAc, (g) *o*-$CH_2$-DAc, (i) *m*-DAc crystals.

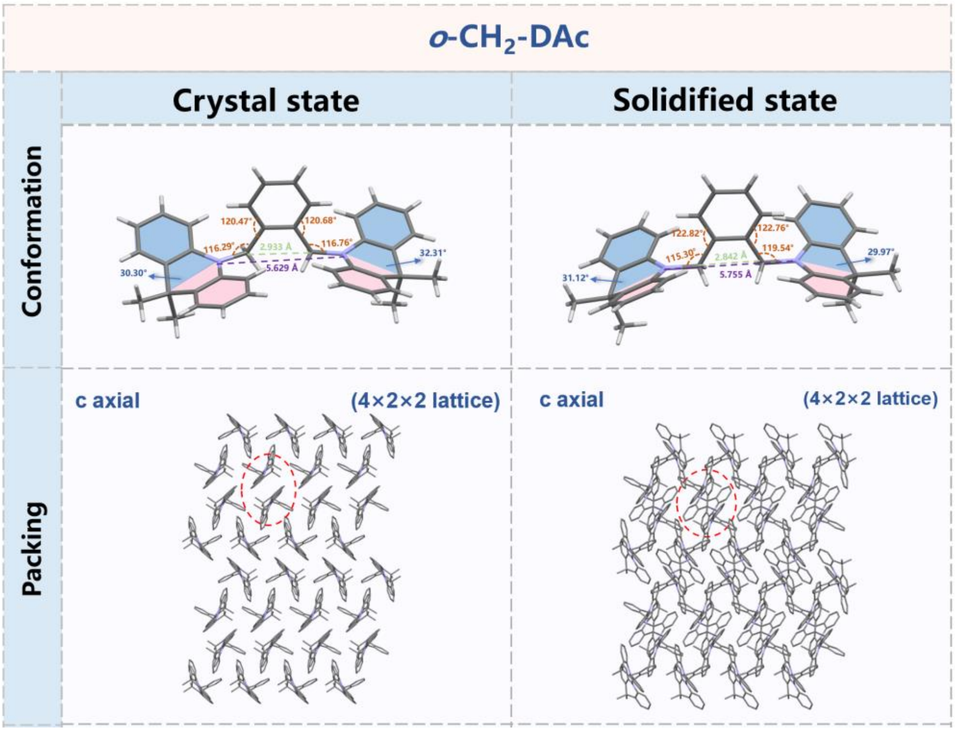


**Figure S36.** Comparison of molecular conformation and packing of *o*-$CH_2$-DAc at the crystal state and solidified state.

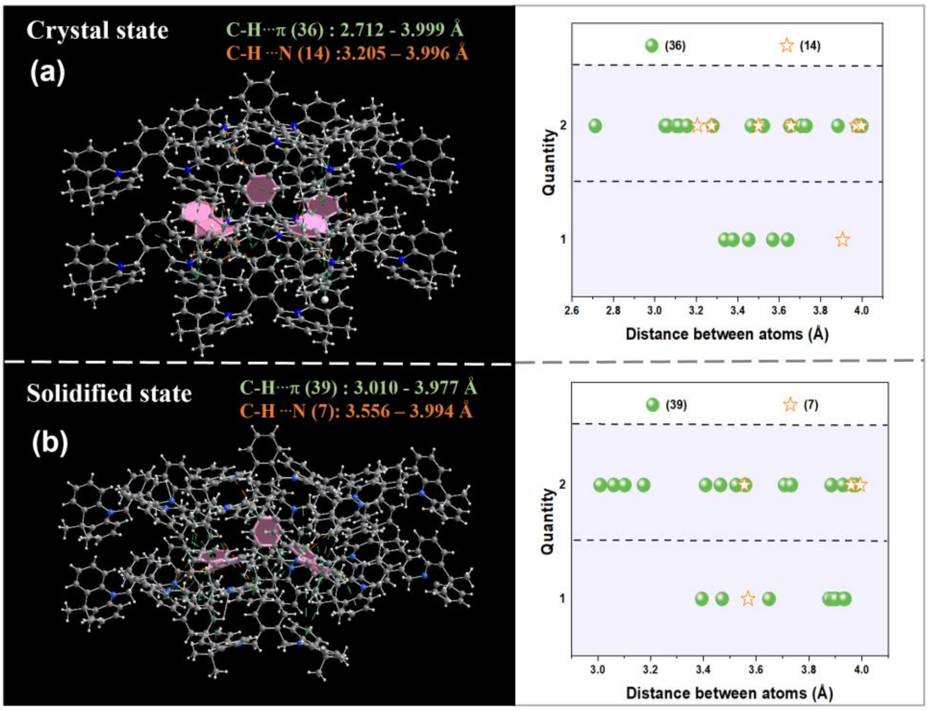


**Figure S37.** (a) The interactions between a given molecule and adjacent molecules in *o*-$CH_2$-DAc crystal. (b) The interactions between a given molecule and adjacent molecules for *o*-$CH_2$-DAc at solidified state.

**Table S3.** The interactions between a given molecule and adjacent molecules in *o*-$CH_2$-DAc crystal

| *o*-$CH_2$-DAc | C-H···π | Number | C-H···N | Number |
|---|---|---|---|---|
| Crystal state | 2.712 Å (2); 3.050 Å (2); 3.059 Å (2); 3.104 Å (2); 3.117 Å (2); 3.155 Å (2); 3.280 Å (2); 3.338 Å; 3.378 Å; 3.453 Å; 3.466 Å (2); 3.524 Å (2); 3.570 Å (2); 3.641 Å; 3.651 Å (2); 3.707 Å (2); 3.730 Å (2); 3.884 Å (2); 3.978 Å (2); 3.999 Å (2) | 36 | 3.205 Å (2); 3.273 Å (2); 3.500 Å (2); 3.657 Å (2); 3.906 Å; (2) 3.971 Å (2); 3.996 Å (2); | 14 |
| Solidified state | 3.010 Å (2); 3.060 Å (2); 3.102 Å (2); 3.174 Å (2); 3.394 Å; 3.408 Å (2); 3.465 Å (2); 3.471 Å; 3.524 Å (2); 3.559 Å (2); 3.648 Å; 3.708Å (2); 3.733 Å (2); 3.876 Å (2); 3.884 Å (2); 3.893 Å (2); 3.902 Å; 3.929 Å (2); 3.931 Å; 3.935 Å; 3.936 Å; 3.967 Å (2); 3.977 Å (2) | 39 | 3.556 Å (2); 3.569 Å (1); 3.959 Å (2); 3.994 Å (2) | 7 |

**Table S4.** Comparison of intermolecular interactions in *o*-$CH_2$-DAc dimer at crystal state and solidified state.

| **Dimer** | |
|---|---|
| 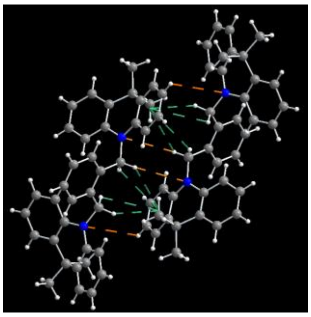 | 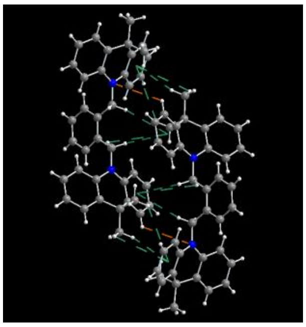 |
| **Crystal state** | **Solidified state** |
| **Distances of C-H···π (Å)** | |
| 3.059 (2) | 3.465 (2) |
| 3.524 (2) | - |
| 3.466 (2) | 3.977 (2) |
| 3.651 (2) | 3.060 (2) |
| - | 3.010 (2) |
| - | 3.935 (2) |
| - | 3.967 (2) |
| **Distances of C-H···N (Å)** | |
| 3.906 (2) | 3.569 (2) |
| 3.205 (2) | - |

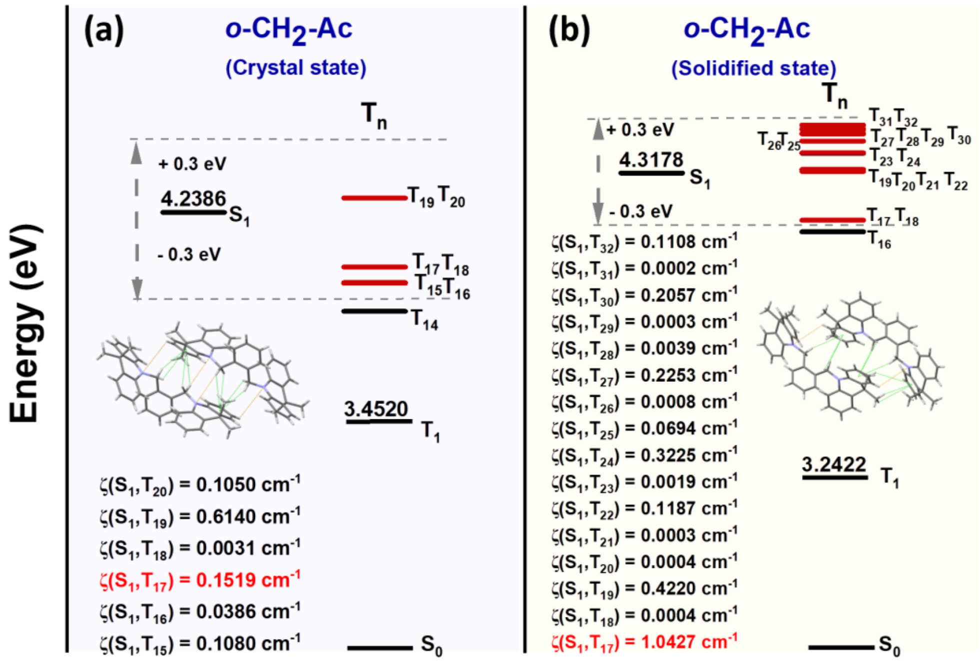


**Figure S38.** Calculated energy levels and corresponding spin-orbit coupling (SOC) constants of *o*-$CH_2$-DAc dimer at (a) the crystal state and (b) the solidified state.

**Table S5.** The calculated energy levels of excited singlet ($S_1$) and triplet ($T_n$) states for *o*-$CH_2$-DAc dimer at crystal state and solidified state by TD-DFT.

| *o*-$CH_2$-DAc (Dimer) | | | | | |
|---|---|---|---|---|---|
| Crystal state | | | Solidified state | | |
| **Isolated** | **n-th** | **Energy (eV)** | **Isolated** | **n-th** | **Energy (eV)** |
| **Sn** | 1 | **4.2386** | **Sn** | 1 | **4.3178** |
| **Tn** | 1 | 3.4520 | **Tn** | 1 | 3.2422 |
| | 2 | 3.4528 | | 2 | 3.2422 |
| | 3 | 3.4697 | | 3 | 3.3677 |
| | 4 | 3.4698 | | 4 | 3.3681 |
| | 5 | 3.7106 | | 5 | 3.5329 |
| | 6 | 3.7110 | | 6 | 3.5330 |
| | 7 | 3.7183 | | 7 | 3.5892 |
| | 8 | 3.7186 | | 8 | 3.5893 |
| | 9 | 3.8093 | | 9 | 3.6623 |
| | 10 | 3.8097 | | 10 | 3.6625 |
| | 11 | 3.8169 | | 11 | 3.7270 |
| | 12 | 3.8171 | | 12 | 3.7270 |
| | 13 | 3.8289 | | 13 | 3.7563 |
| | 14 | 3.8243 | | 14 | 3.7572 |

| | | | | | |
|---|---|---|---|---|---|
| | **15** | **3.9416** | | 15 | 3.9754 |
| | **16** | **3.9448** | | 16 | 3.9754 |
| | **17** | **4.0097** | | **17** | **4.0435** |
| | **18** | **4.0106** | | **18** | **4.0441** |
| | **19** | **4.2983** | | **19** | **4.3314** |
| | **20** | **4.2988** | | **20** | **4.3317** |
| | | | | **21** | **4.3438** |
| | | | | **22** | **4.3441** |
| | | | | **23** | **4.4393** |
| | | | | **24** | **4.4410** |
| | | | | **25** | **4.5104** |
| | | | | **26** | **4.5108** |
| | | | | **27** | **4.5537** |
| | | | | **28** | **4.5555** |
| | | | | **29** | **4.5787** |
| | | | | **30** | **4.5835** |
| | | | | **31** | **4.6051** |
| | | | | **32** | **4.6057** |

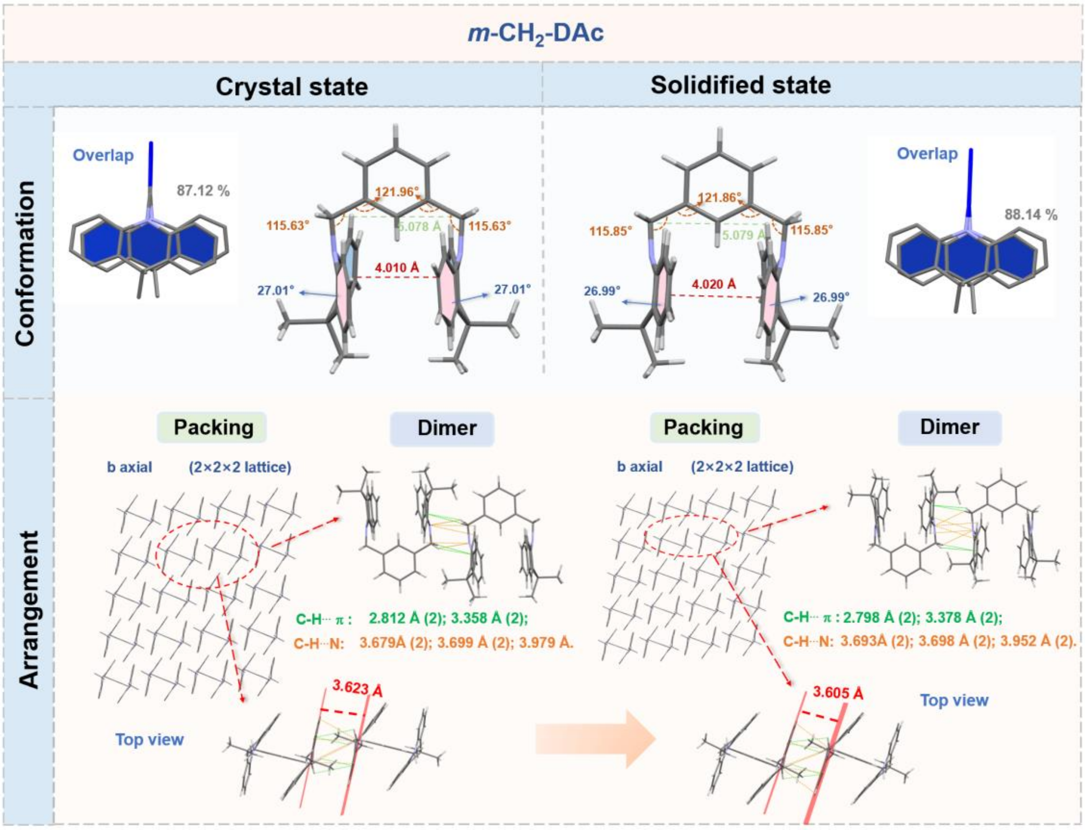


**Figure S39.** Comparison of molecular conformations and packing of $m$-$CH_2$-DAc at the crystal state and solidified state.

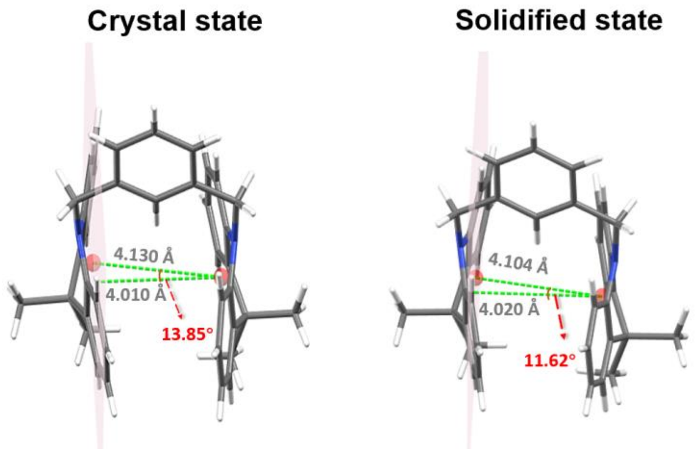


**Figure S40.** Comparison of molecular conformations of *m*-$CH_2$-DAc at different states by the different distances between two acridine moieties.

**Table S6.** Comparison of *m*-$CH_2$-DAc dimer 1 at crystal and solidified state.

| Dimer 1 | |
|---|---|
| 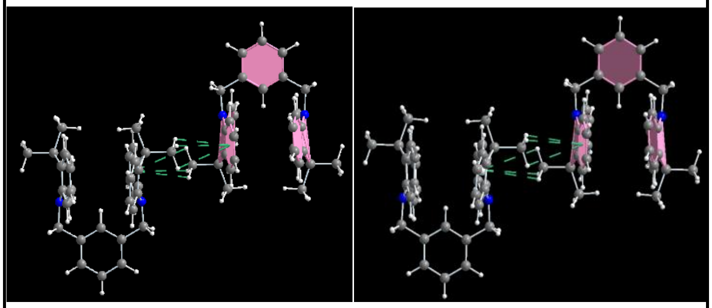 | |
| Crystal state | Solidified state |
| Distances of C-H···π (Å) | |
| 3.808 | 3.848 |
| 3.263 | 3.322 |
| 3.526 | 3.540 |
| 3.526 | 3.540 |
| 3.808 | 3.848 |
| 3.263 | 3.322 |

**Table S7.** Comparison of *m*-$CH_2$-DAc dimer 2 at crystal and solidified state.

| Dimer 2 | |
|---|---|
| 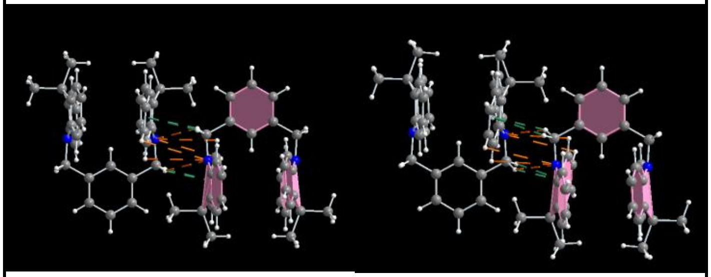 | |
| Crystal state | Solidified state |
| Distances of C-H···π (Å) | |
| 3.358 | 3.378 |
| 2.812 | 2.798 |
| 2.812 | 2.798 |
| 3.358 | 3.378 |
| Distances of C-H···N (Å) | |
| 3.979 | 3.952 |
| 3.679 | 3.694 |
| 3.699 | 3.698 |
| 3.699 | 3.698 |
| 3.679 | 3.694 |
| 3.979 | 3.952 |

**Table S8.** Comparison of *m*-$CH_2$-DAc dimer 3 at crystal and solidified state.

| Dimer 3 | |
|---|---|
| 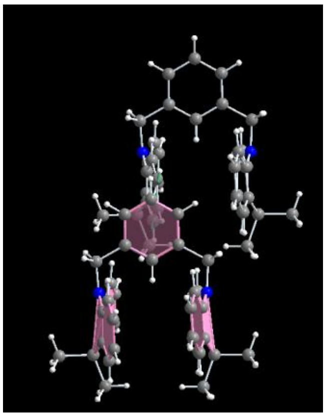 | 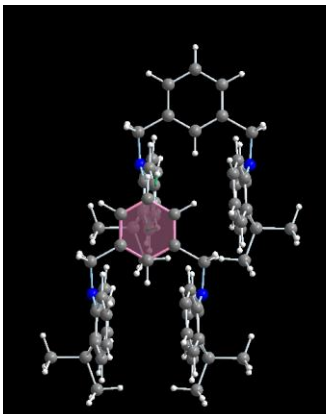 |
| Crystal state | Solidified state |
| Distances of C-H···π (Å) | |
| 3.744 | 3.730 |
| 3.012 | 2.955 |

**Table S9.** Comparison of *m*-$CH_2$-DAc dimer 4 at crystal and solidified state.

| Dimer 4 | |
|---|---|
| 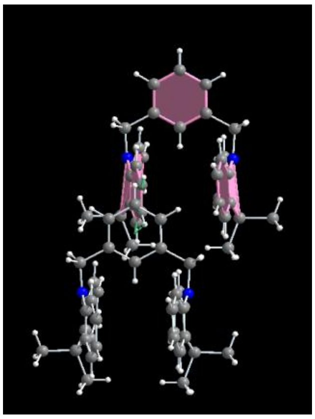 | 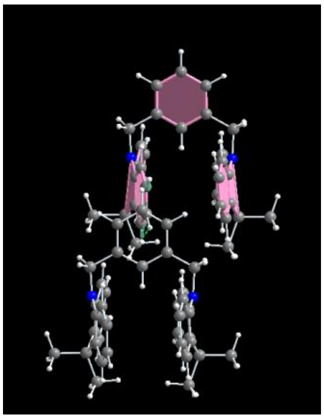 |
| Crystal state | Solidified state |
| Distances of C-H···π (Å) | |
| 3.744 | 3.730 |
| 3.012 | 2.955 |

**Table S10.** Comparison of *m*-$CH_2$-DAc dimer 5 at crystal and solidified state.

| Dimer 5 | |
|---|---|
| 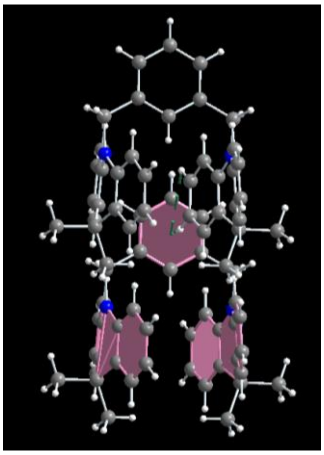 | 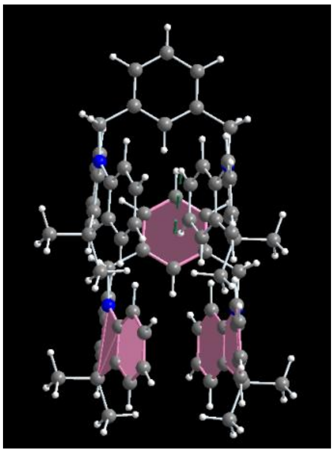 |
| Crystal state | Solidified state |
| Distances of C-H···π (Å) | |
| 3.744 | 3.730 |
| 3.012 | 2.955 |

**Table S11.** Comparison of *m*-$CH_2$-DAc dimer 6 at crystal and solidified state.

| Dimer 6 | |
|---|---|
| 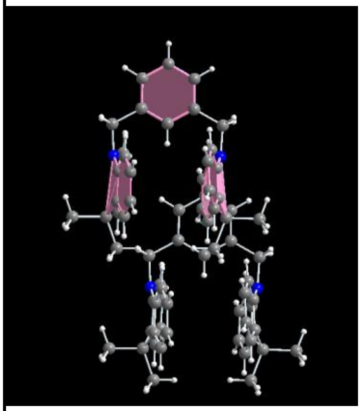 | 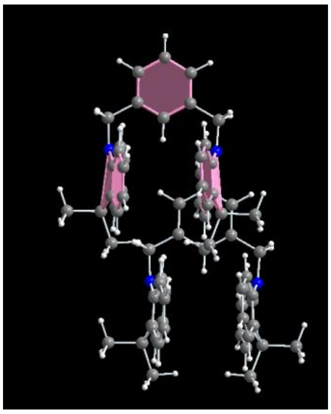 |
| Crystal state | Solidified state |
| Distances of C-H···π (Å) | |
| 3.744 | 3.730 |
| 3.012 | 2.955 |

**Table S12.** Comparison of *m*-$CH_2$-DAc dimer 7 at crystal and solidified state.

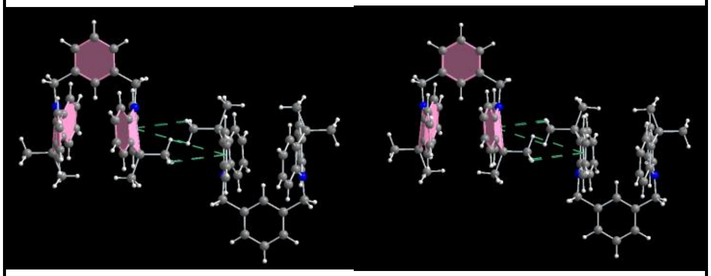

| Dimer 7 | |
|---|---|
| Crystal state | Solidified state |
| Distances of C-H···π (Å) | |
| 3.808 | 3.848 |
| 3.263 | 3.322 |
| 3.526 | 3.540 |
| 3.526 | 3.540 |
| 3.263 | 3.322 |
| 3.808 | 3.848 |

**Table S13.** Comparison of *m*-$CH_2$-DAc dimer 8 at crystal and solidified state.

| Dimer 8 | |
|---|---|
| 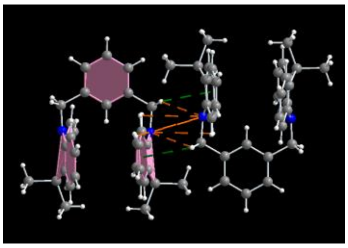 | 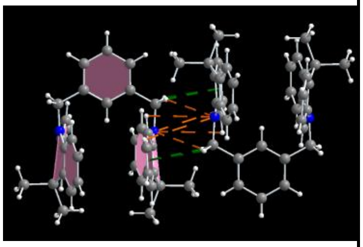 |
| Crystal state | Solidified state |
| Distances of C-H···π (Å) | |
| 3.358 | 3.378 |
| 2.812 | 2.798 |
| 2.812 | 2.798 |
| 3.358 | 3.378 |
| Distances of C-H···N (Å) | |
| 3.699 | 3.698 |
| 3.679 | 3.694 |
| 3.979 | 3.952 |
| 3.979 | 3.952 |
| 3.679 | 3.694 |
| 3.699 | 3.698 |

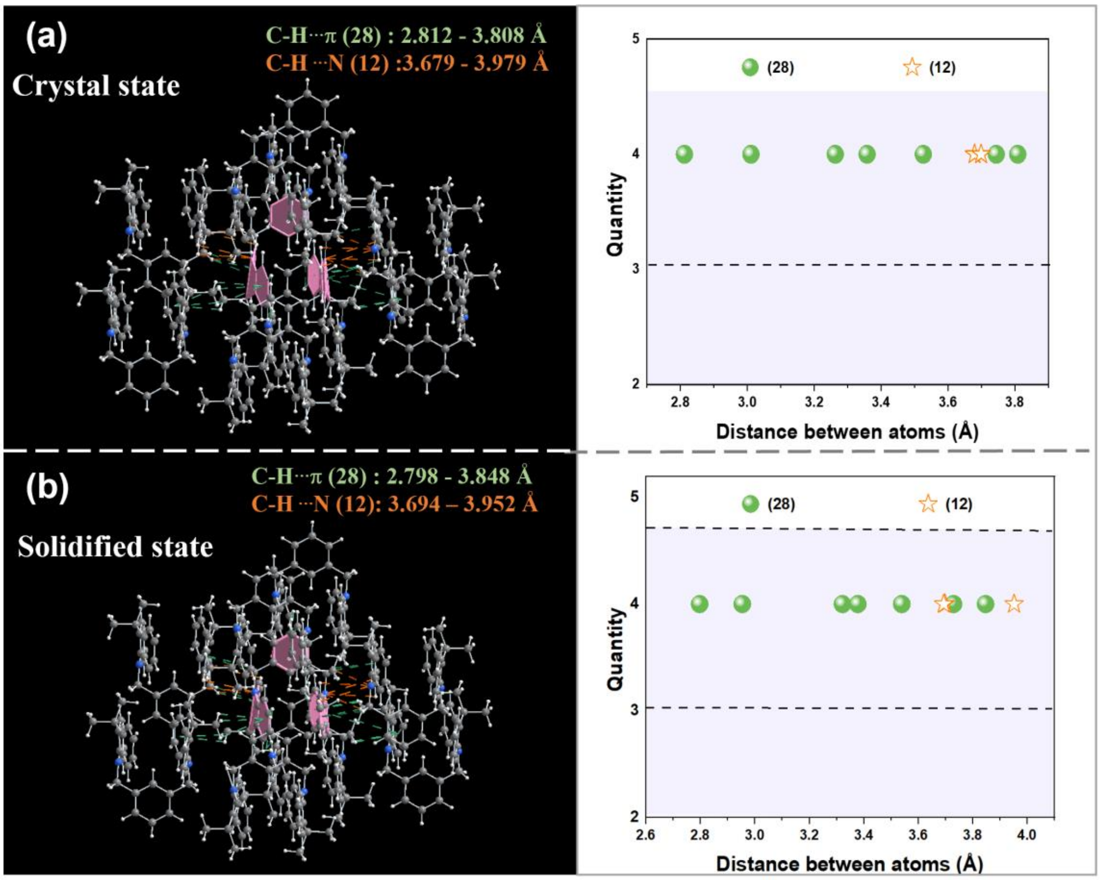


**Figure S41.** (a) The interactions between a given molecule and adjacent molecules in *m*-$CH_2$-DAc crystal. (b) The interactions between a given molecule and adjacent molecules for *m*-$CH_2$-DAc at solidified state.

**Table S14.** The interactions between a given molecule and adjacent molecules in *m*-$CH_2$-DAc crystal.

| ***m*-$CH_2$-DAc** | **C-H…π** | **Number** | **C-H…N** | **Number** |
|---|---|---|---|---|
| **Crystal state** | 2.812 Å (4); 3.011 Å (4); 3.263 Å (4); 3.358 Å (4); 3.526 Å (4); 3.744 Å (4); 3.808 Å (4) | 28 | 3.679 Å (4); 3.699 Å (4); 3.979 Å (4) | 12 |
| **Solidified state** | 2.798 Å (4); 2.955 Å (4); 3.322 Å (4); 3.378 Å (4); 3.540 Å (4); 3.730 Å (4); 3.848 Å (4) | 28 | 3.694 Å (4); 3.698 Å (4); 3.952 Å (4) | 12 |

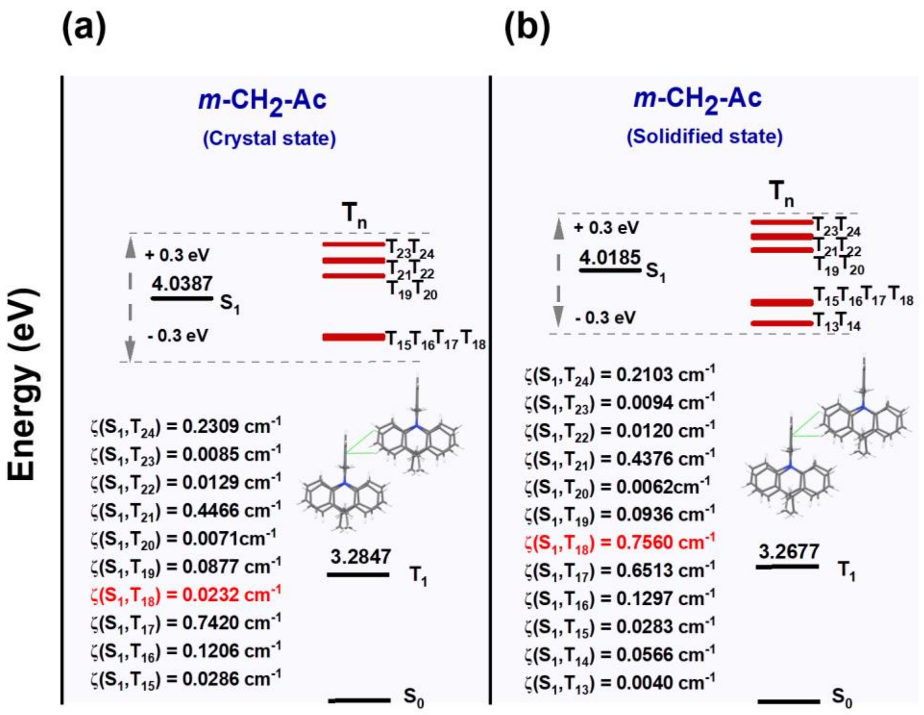


**Figure S42.** Calculated energy levels and corresponding spin-orbit coupling (SOC) constants for the *m*-$CH_2$-DAc dimer 3 at (a) crystal state and (b) solidified state.

**Table S15.** The calculated energy levels of excited singlet ($S_1$) and triplet ($T_n$) states for *m*-$CH_2$-DAc dimer 3 at crystal and solidified state by TD-DFT.

| *m*-$CH_2$-DAc (Dimer 3) | | | | | |
|---|---|---|---|---|---|
| Crystal state | | | Solidified state | | |
| **Isolated** | **n-th** | **Energy (eV)** | **Isolated** | **n-th** | **Energy (eV)** |
| **Sn** | 1 | **4.0387** | **Sn** | 1 | **4.0185** |
| **Tn** | 1 | 3.2847 | **Tn** | 1 | 3.2677 |
| | 2 | 3.2879 | | 2 | 3.2697 |
| | 3 | 3.3182 | | 3 | 3.2993 |
| | 4 | 3.3205 | | 4 | 3.3004 |
| | 5 | 3.6125 | | 5 | 3.5900 |
| | 6 | 3.6156 | | 6 | 3.5930 |
| | 7 | 3.6239 | | 7 | 3.6148 |
| | 8 | 3.6243 | | 8 | 3.6155 |
| | 9 | 3.6578 | | 9 | 3.6424 |

| | | | | | |
|---|---|---|---|---|---|
| | 10 | 3.6586 | | 10 | 3.6424 |
| | 11 | 3.6707 | | 11 | 3.6589 |
| | 12 | 3.6760 | | 12 | 3.6647 |
| | 13 | 3.7268 | | **13** | **3.7199** |
| | 14 | 3.7320 | | **14** | **3.7240** |
| | **15** | **3.8451** | | **15** | **3.8301** |
| | **16** | **3.8524** | | **16** | **3.8344** |
| | **17** | **3.8594** | | **17** | **3.8460** |
| | **18** | **3.8615** | | **18** | **3.8460** |
| | **19** | **4.1407** | | **19** | **4.1272** |
| | **20** | **4.1476** | | **20** | **4.1346** |
| | **21** | **4.2137** | | **21** | **4.2005** |
| | **22** | **4.2258** | | **22** | **4.2136** |
| | **23** | **4.2965** | | **23** | **4.2856** |
| | **24** | **4.3599** | | **24** | **4.2877** |

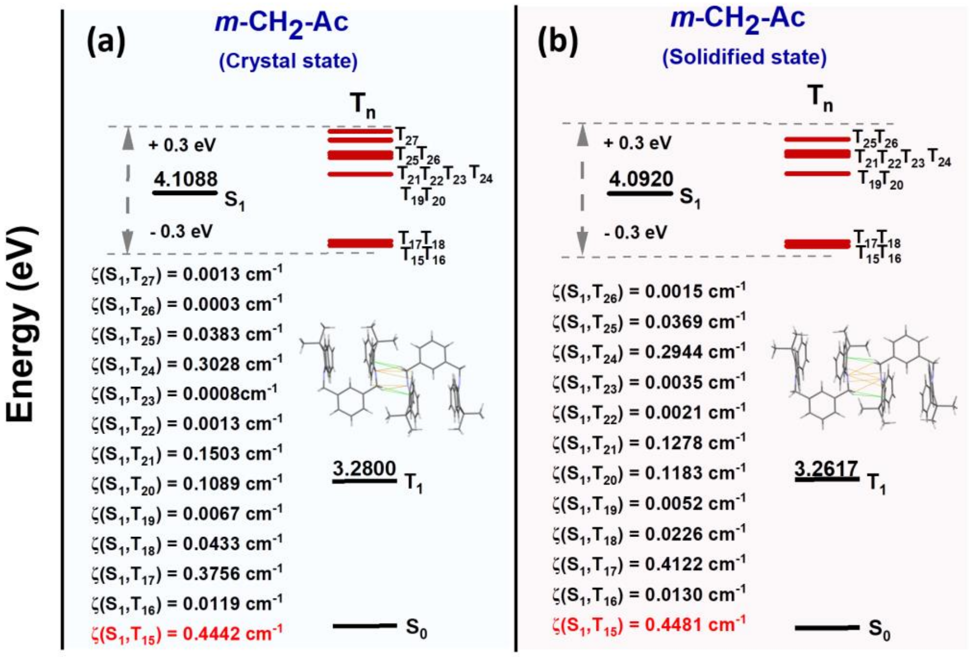


**Figure S43.** Calculated energy levels and corresponding spin-orbit coupling (SOC) constants for *m*-$CH_2$-DAc dimer 2 at (a) crystal state and (b) solidified state.

**Table S16.** The calculated excited singlet ($S_1$) and triplet ($T_n$) states for *m*-$CH_2$-DAc dimer 2 at crystal and solidified state by TD-DFT.

| *m*-$CH_2$-DAc (Dimer 2) | | | | | |
|---|---|---|---|---|---|
| Crystal state | | | Solidified state | | |
| **Isolated** | **n-th** | **Energy (eV)** | **Isolated** | **n-th** | **Energy (eV)** |
| **Sn** | 1 | **4.1088** | **Sn** | 1 | **4.0920** |
| **Tn** | 1 | 3.2800 | **Tn** | 1 | 3.2617 |
| | 2 | 3.2820 | | 2 | 3.2638 |
| | 3 | 3.3154 | | 3 | 3.2956 |
| | 4 | 3.3164 | | 4 | 3.2966 |
| | 5 | 3.6154 | | 5 | 3.5934 |
| | 6 | 3.6164 | | 6 | 3.5935 |
| | 7 | 3.6171 | | 7 | 3.6169 |
| | 8 | 3.6177 | | 8 | 3.6172 |
| | 9 | 3.6538 | | 9 | 3.6404 |
| | 10 | 3.6539 | | 10 | 3.6407 |
| | 11 | 3.6690 | | 11 | 3.6486 |
| | 12 | 3.6698 | | 12 | 3.6493 |
| | 13 | 3.7199 | | 13 | 3.7099 |
| | 14 | 3.7203 | | 14 | 3.7107 |
| | **15** | **3.8529** | | **15** | **3.8387** |
| | **16** | **3.8561** | | **16** | **3.8413** |
| | **17** | **3.8751** | | **17** | **3.8605** |
| | **18** | **3.8758** | | **18** | **3.8609** |
| | **19** | **4.1088** | | **19** | **4.1875** |
| | **20** | **4.1993** | | **20** | **4.1879** |
| | **21** | **4.2790** | | **21** | **4.2713** |
| | **22** | **4.2919** | | **22** | **4.2838** |
| | **23** | **4.3007** | | **23** | **4.2893** |
| | **24** | **4.3039** | | **24** | **4.2922** |
| | **25** | **4.3640** | | **25** | **4.3513** |
| | **26** | **4.3659** | | **26** | **4.3528** |
| | **27** | **4.4072** | | 27 | 4.3949 |

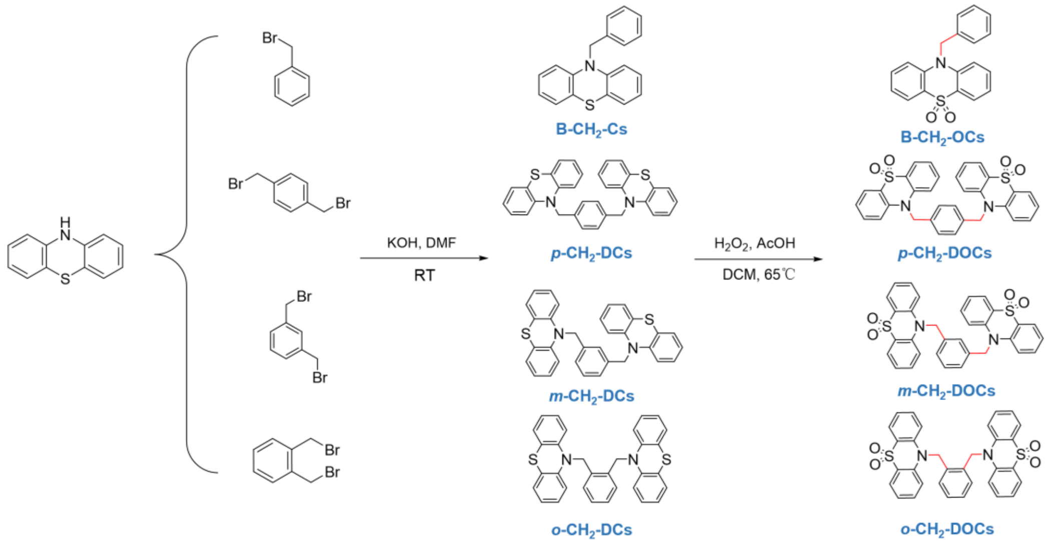


**Figure S44.** The synthetic routes of B-$CH_2$-OCs, *p*-$CH_2$-DOCs, *m*-$CH_2$-DOCs and *o*-$CH_2$-DOCs.

## Synthesis of B-$CH_2$-Cs

Phenothiazine (0.50 g, 2.50 mmol) and potassium hydroxide (1.40 g, 25.00 mol) were placed in a round bottom flask, dissolved with dimethyl sulfoxide (10 mL), and stirred at room temperature for 1 h. Benzyl bromide (0.59 mL, 5.00 mmol) was added and stirred at room temperature for 12 h. Then, a large amount of $H_2O$ was added, and the filter residue was collected after filtration. The crude product was purified by a silica gel column using petroleum ether and dichloromethane as eluent to give **B-$CH_2$-Cs** as a white solid (369 mg, 51%). $^1$H NMR (400 MHz, Chloroform-*d*) δ 7.33-7.26 (m, 4H, ArH), 7.25-7.20 (m, 1H, ArH), 7.06 (dd, $J$ = 7.5, 1.6 Hz, 2H, ArH), 6.94 (td, $J$ = 8.1, 1.6 Hz, 2H, ArH), 6.83 (m, $J$ = 7.5, 1.0 Hz, 2H, ArH), 6.64-6.58 (m, 2H, ArH), 5.06 (s, 2H, $CH_2$). $^{13}$C NMR (100 MHz, Chloroform-*d*) δ 144.51, 136.70, 128.77, 127.27, 127.05, 126.87, 126.65, 123.17, 122.55, 115.50, 52.73.

## The general synthesis route of *p*-$CH_2$-DCs, *m*-$CH_2$-DCs and *o*-$CH_2$-DCs.

Phenothiazine (1.9 eq.) and potassium hydroxide (20.0 eq.) were placed in a round bottom flask, dissolved with dimethyl sulfoxide, and stirred at room temperature for 1 h. Dibromomethyl benzene derivatives (1.0 eq.) were added in small amounts in batches and stirred at room temperature for another 12 h. Then, a large amount of $H_2O$ was added, and then the filter residue was collected after

filtration. The crude product was purified by a silica gel column using petroleum ether and dichloromethane as eluent to obtain the desired compounds.

***p*-$CH_2$-DCs:** A white solid (169 mg, 17.80%). $^1$H NMR (400 MHz, Chloroform-*d*) δ 7.52 (s, 2H, ArH), 7.02-6.96 (m, 10H, ArH), 6.83 (t, *J* = 6.9 Hz, 4H, ArH), 6.57 (d, *J* = 1.1 Hz, 2H, ArH), 6.55 (d, *J* = 1.5 Hz, 2H, ArH), 5.30 (s, 4H, $CH_2$).

***m*-$CH_2$-DCs:** A white solid (414 mg, 43.72%). $^1$H NMR (400 MHz, Chloroform-*d*) δ 7.28 (d, *J* = 5.7 Hz, 2H, ArH), 7.22 (d, *J* = 7.6 Hz, 2H, ArH), 7.07 (dd, *J* = 7.5, 1.3 Hz, 4H, ArH), 6.96-6.91 (m, 4H, ArH), 6.85 (t, *J* = 7.4 Hz, 4H, ArH), 6.57 (d, *J* = 8.0 Hz, 4H, ArH), 5.02 (s, 4H, $CH_2$). $^{13}$C NMR (100 MHz, Chloroform-*d*) δ 144.27, 129.19, 127.26, 127.19, 127.06, 123.89, 122.69, 115.71, 50.63.

***o*-$CH_2$-DCs:** A white solid (222 mg, 23.45%). $^1$H NMR (400 MHz, Chloroform-*d*) δ 7.33 (dd, *J* = 5.4, 3.5 Hz, 2H, ArH), 7.17 (dd, *J* = 5.6, 3.4 Hz, 3H, ArH), 7.08 (dd, *J* = 7.5, 1.3 Hz, 5H, ArH), 6.98 (t, *J* = 7.3 Hz, 5H, ArH), 6.92-6.82 (m, 4H, ArH), 6.64 (d, *J* = 8.0 Hz, 4H, ArH), 5.21 (s, 4H, $CH_2$).

**The general synthesis routes of B-$CH_2$-OCs, *p*-$CH_2$-DOCs, *o*-$CH_2$-DOCs and *m*-$CH_2$-DOCs.**

The phenothiazine derivative (1.0 equiv.) dissolved in DCM was placed into a round bottom flask, acetic acid (20 equiv.) and hydrogen peroxide (20 equiv.) were added, and the reaction mixtures were stirred and reflux for 12 h at 65 ℃. After cooled to room temperature, the reaction mixture was extracted with DCM and the organic phase was collected on a column using pure DCM or PE/EA (1/4) as the eluent.

**B-$CH_2$-OCs:** A white solid (320 mg, 78.42%). mp: 192 $^o$C. $^1$H NMR (400 MHz, Chloroform-*d*) δ 7.98 (dd, *J* = 7.7, 1.5 Hz, 2H, ArH), 7.52-7.46 (m, 2H, ArH), 7.33 (dd, *J* = 11.3, 7.2 Hz, 3H, ArH), 7.25 (t, *J* = 7.4 Hz, 3H, ArH), 7.21 (s, 1H, ArH), 7.18 (s, 1H, ArH), 7.15 (d, *J* = 7.0 Hz, 2H, ArH), 5.51 (s, 2H, $CH_2$). $^{13}$C NMR (100 MHz, Chloroform-*d*) δ 138.92, 135.23, 133.04, 131.52, 129.17, 127.59, 125.84, 124.34, 122.19, 116.59, 52.81. MS (EI) calcd. for $C_{19}H_{15}NO_2S$ $[M]^+$: 321.08; Found: 321.10.

***p*-$CH_2$-DOCs:** A white solid (30 mg, 15.74%). mp: 380 $^o$C. $^1$H NMR (400 MHz, DMSO-$d_6$) δ 8.03 (dd, *J* = 8.0, 1.5 Hz, 4H, ArH), 7.68-7.63 (m, 4H, ArH), 7.35 (dt, *J* = 7.2, 2.9 Hz, 8H, ArH), 7.14 (s, 4H, ArH), 5.59 (s, 4H, $CH_2$). MS (EI) calcd. for $C_{32}H_{24}N_2O_4S_2$ $[M]^+$: 564.12; Found: 564.35.

***m*-CH$_2$-DOCs:** A white solid (110 mg, 23.56%). mp: 207 °C.$^1$H NMR (400 MHz, DMSO-$d_6$) δ 7.95-7.85 (m, 4H, ArH), 7.43 (t, *J* = 7.7 Hz, 1H, ArH), 7.35 (t, *J* = 7.8 Hz, 4H, ArH), 7.24 (q, *J* = 6.9, 6.2 Hz, 6H, ArH), 7.06 (d, *J* = 8.6 Hz, 4H, ArH), 6.55 (s, 1H, ArH), 5.47 (s, 4H, CH$_2$). $^{13}$C NMR (100 MHz, DMSO-$d_6$) δ 140.54, 136.66, 133.57, 125.73, 124.15, 123.50, 122.75, 122.52, 117.23, 51.30. MS (EI) calcd. for $C_{32}H_{24}N_2O_4S_2$ [M]$^+$: 564.12; Found: 564.35.

***o*-CH$_2$-DOCs:** A white solid (228 mg, 91.07%). mp: 214 °C. $^1$H NMR (400 MHz, DMSO-$d_6$) δ 8.15-8.04 (m, 4H, ArH), 7.81-7.71 (m, 4H, ArH), 7.50-7.39 (m, 8H, ArH), 7.11 (dd, *J* = 5.6, 3.3 Hz, 2H, ArH), 6.77 (dd, *J* = 5.3, 3.5 Hz, 2H, ArH), 5.85 (s, 4H, CH$_2$). $^{13}$C NMR (100 MHz, DMSO-$d_6$) δ 140.75, 134.23, 132.73, 127.87, 126.46, 123.89, 123.07, 122.94, 118.10. MS (EI) calcd. for $C_{32}H_{24}N_2O_4S_2$ [M]$^+$: 564.12; Found: 564.20.

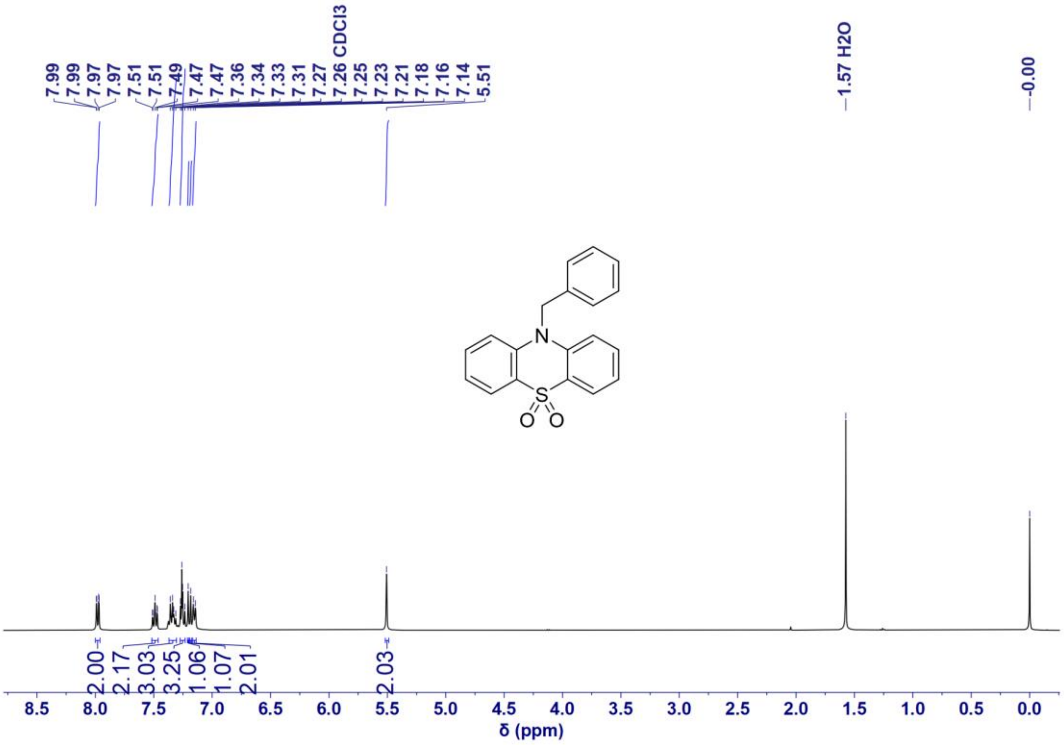


**Figure S45.** $^1$H NMR spectrum of B-CH$_2$-OCs.

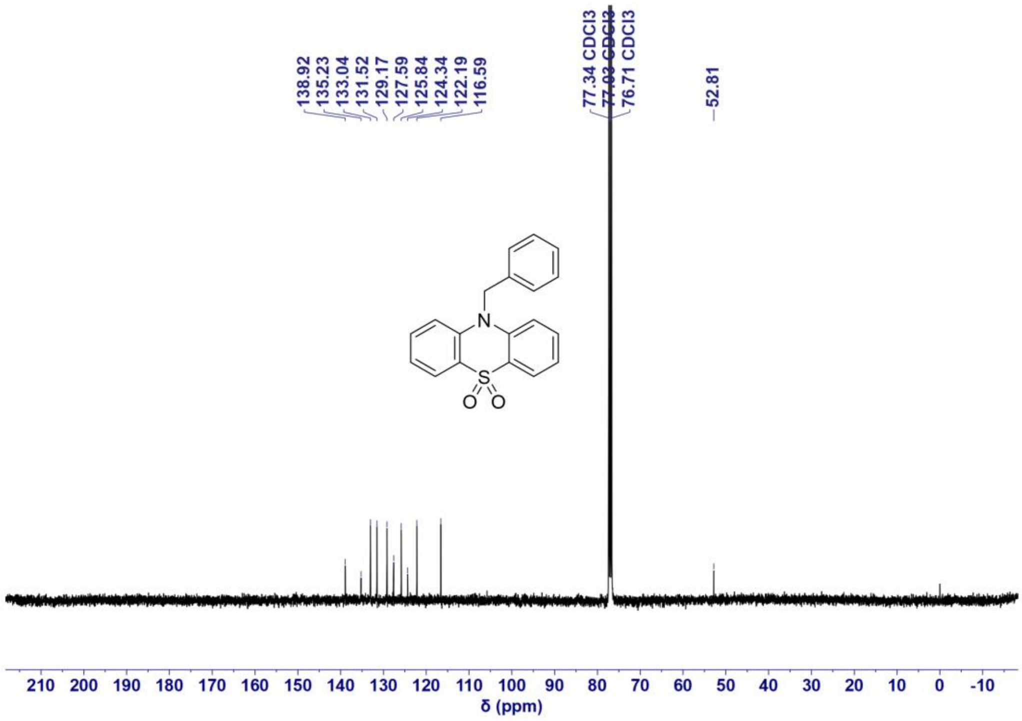


**Figure S46.** $^{13}$C NMR spectrum of B-CH$_2$-OCs.

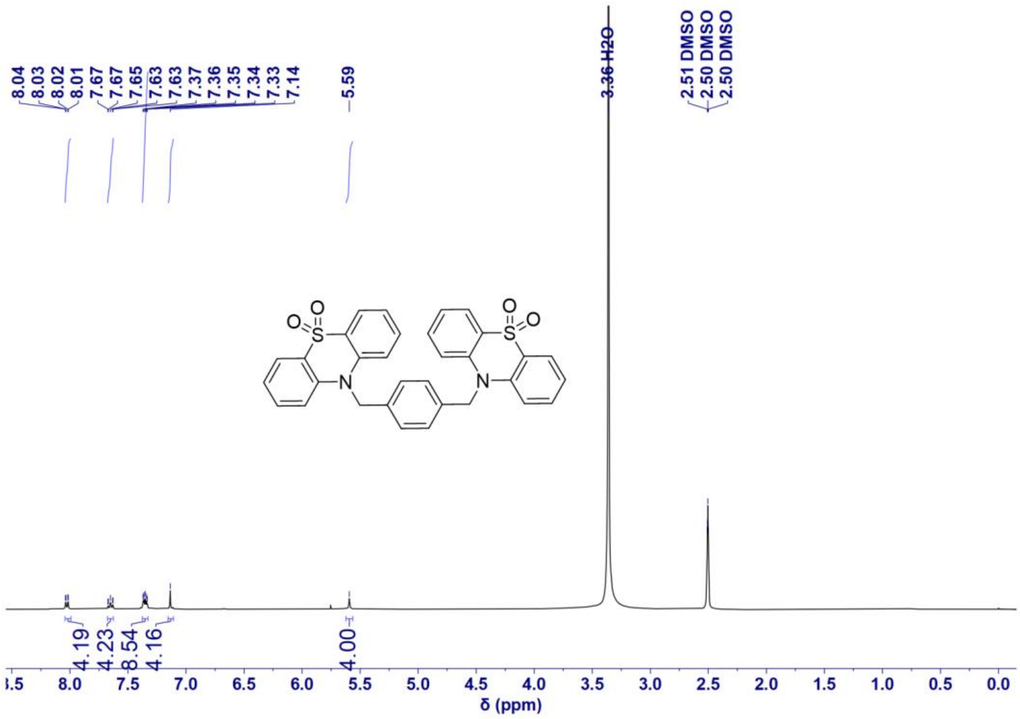


**Figure S47.** $^{1}$H NMR spectrum of *p*-CH$_2$-DOCs.

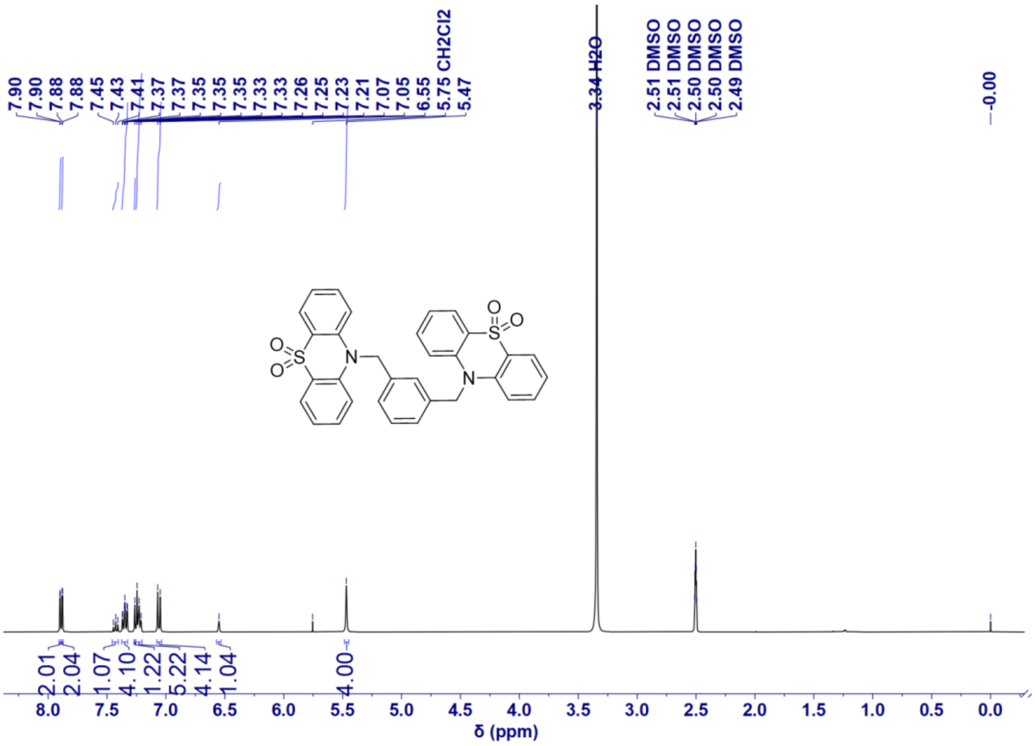


**Figure S48.** $^{1}$H NMR spectrum of *m*-CH$_2$-DOCs.

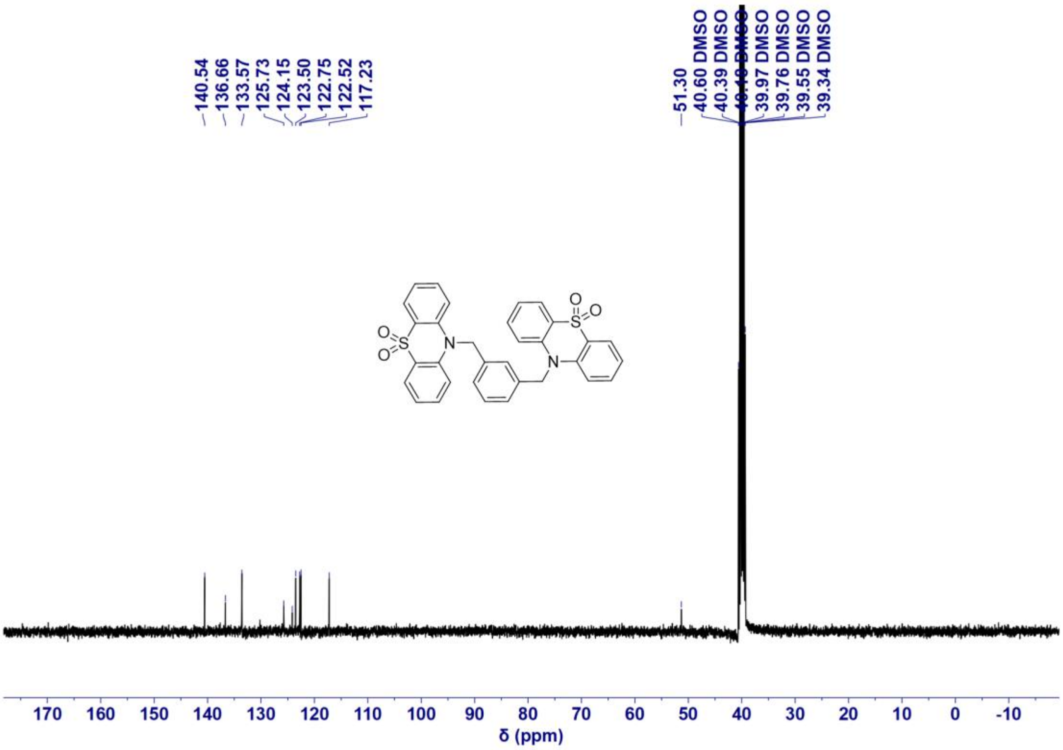


**Figure S49.** $^{13}$C NMR spectrum of *m*-CH$_2$-DOCs.

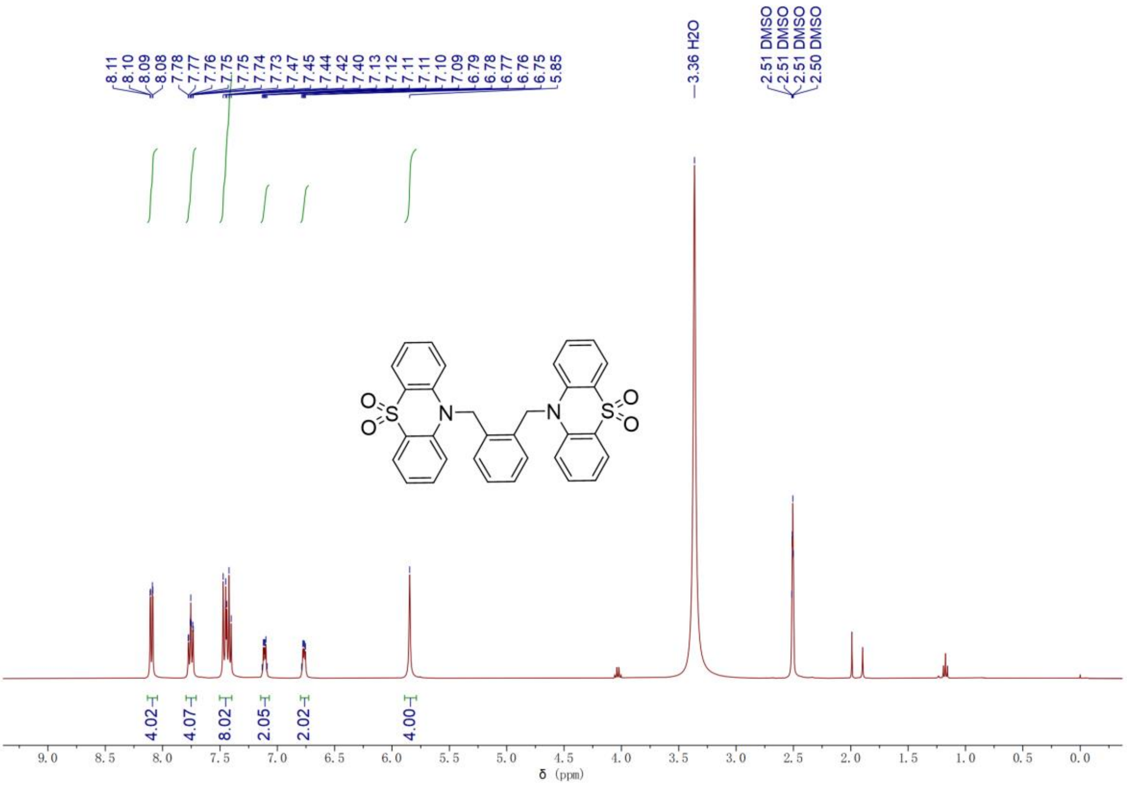

**Figure S50.** $^{1}$H NMR spectrum of *o*-$CH_2$-DOCs.

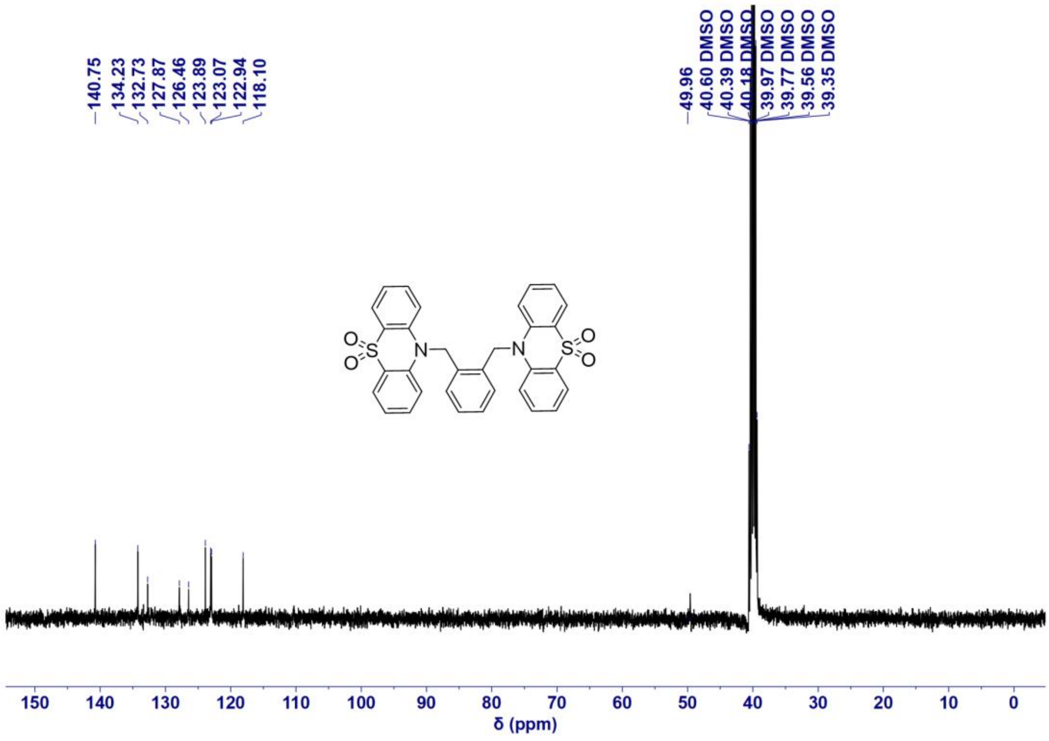

**Figure S51.** $^{13}$C NMR spectrum of *o*-$CH_2$-DOCs.

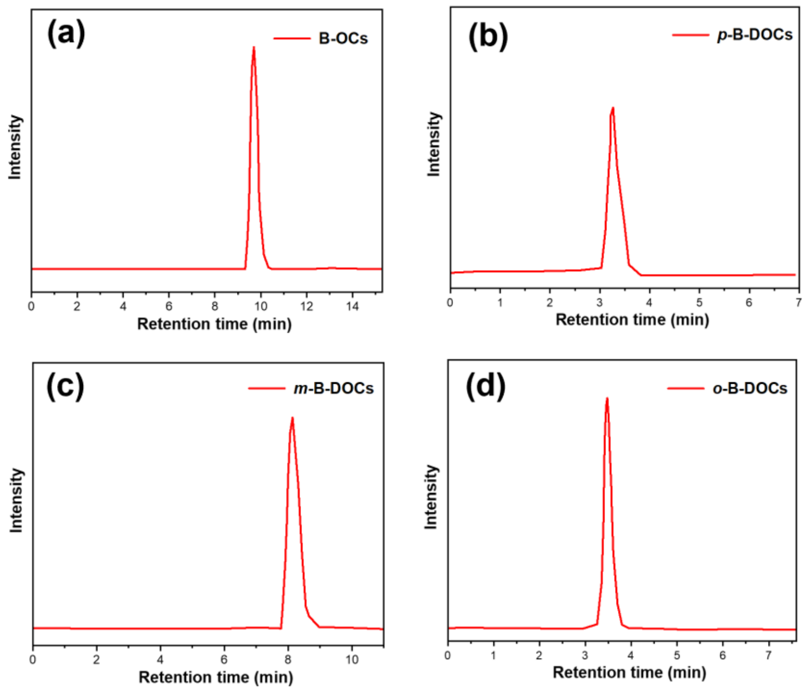


**Figure S52.** High performance liquid chromatogram (HPLC) spectra of (a) B-$CH_2$-OCs at crystal state, (b) *p*-$CH_2$-DOCs at crystal state, (c) *m*-$CH_2$-DOCs at crystal state and (d) *o*-$CH_2$-DOCs at crystal state. (Conducted on Waters 600)

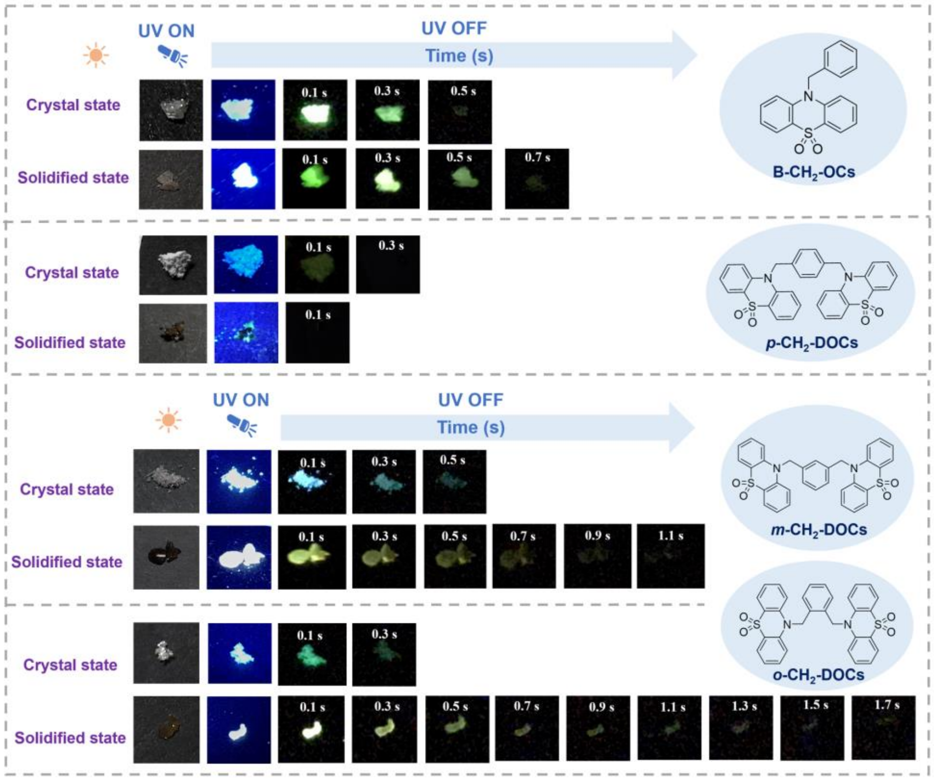


**Figure 53.** Photographs of B-$CH_2$-OCs, *p*-$CH_2$-DOCs, *m*-$CH_2$-DOCs and *o*-$CH_2$-OCs at crystal states and solidified states.

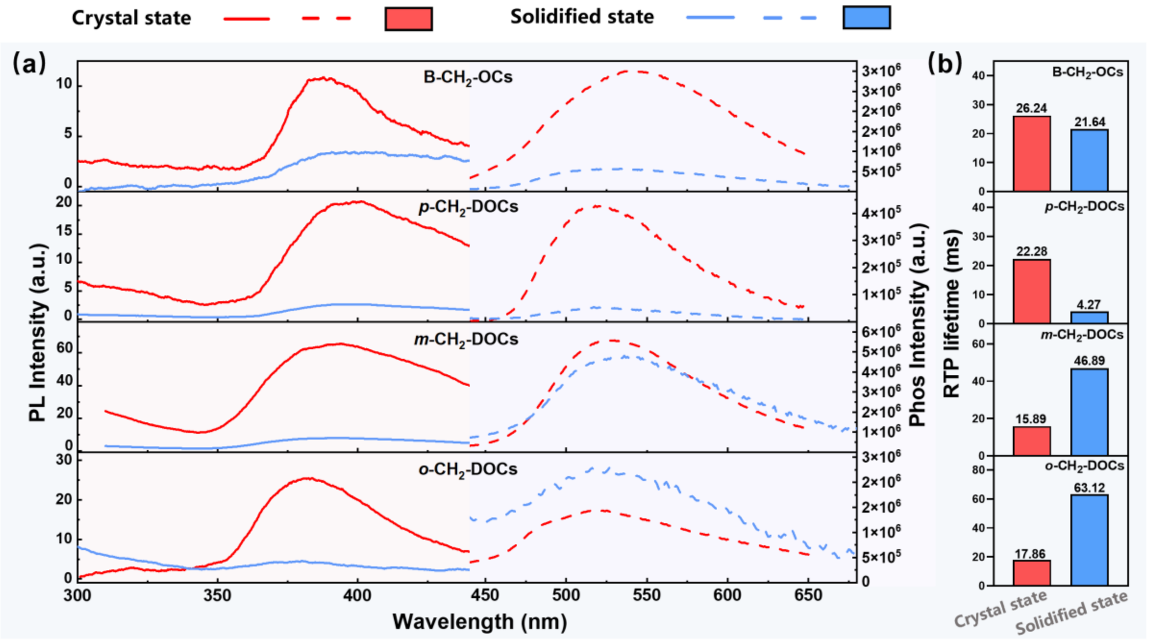


**Figure 54.** (a) Photoluminescence (solid line) and phosphorescence spectra (dotted line) of organic luminogens at crystal state (red) and solidified state (blue) at room-temperature. (b) The corresponding phosphorescence lifetimes of organic luminogens at crystal state (red) and solidified state (blue) at room-temperature.

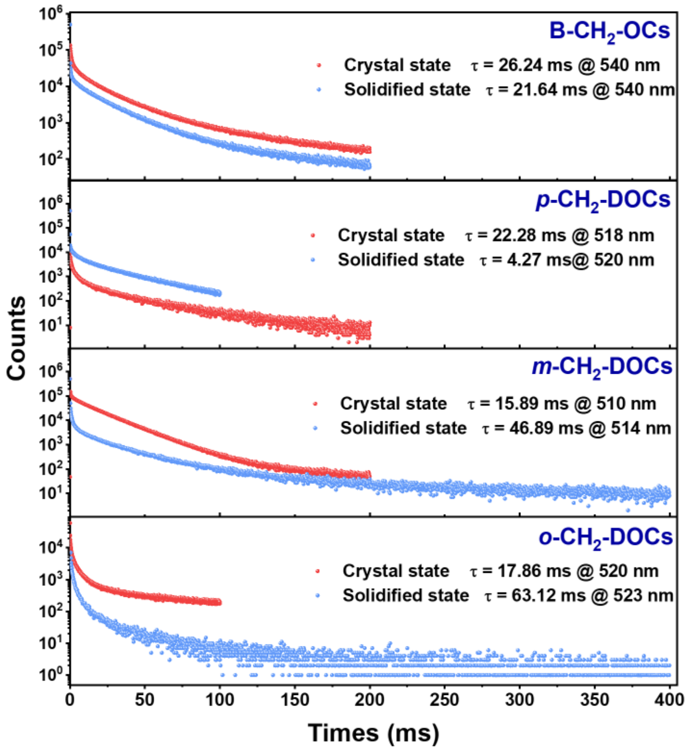


**Figure S55.** The phosphorescence lifetimes of B-$CH_2$-OCs, *p*-$CH_2$-DOCs, *m*-$CH_2$-DOCs and *o*-$CH_2$-DOCs at crystal state (red) and solidified state (blue) at room-temperature.

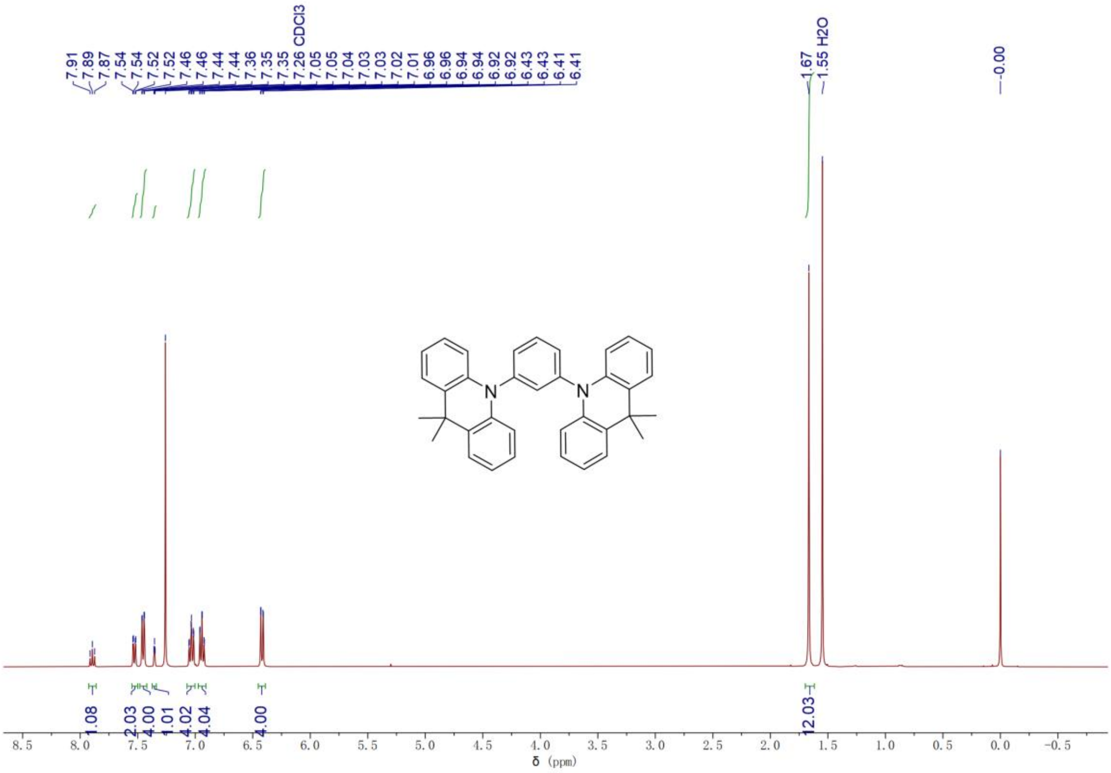


**Figure S56.** $^{1}$H NMR spectrum of *m*-DAc.

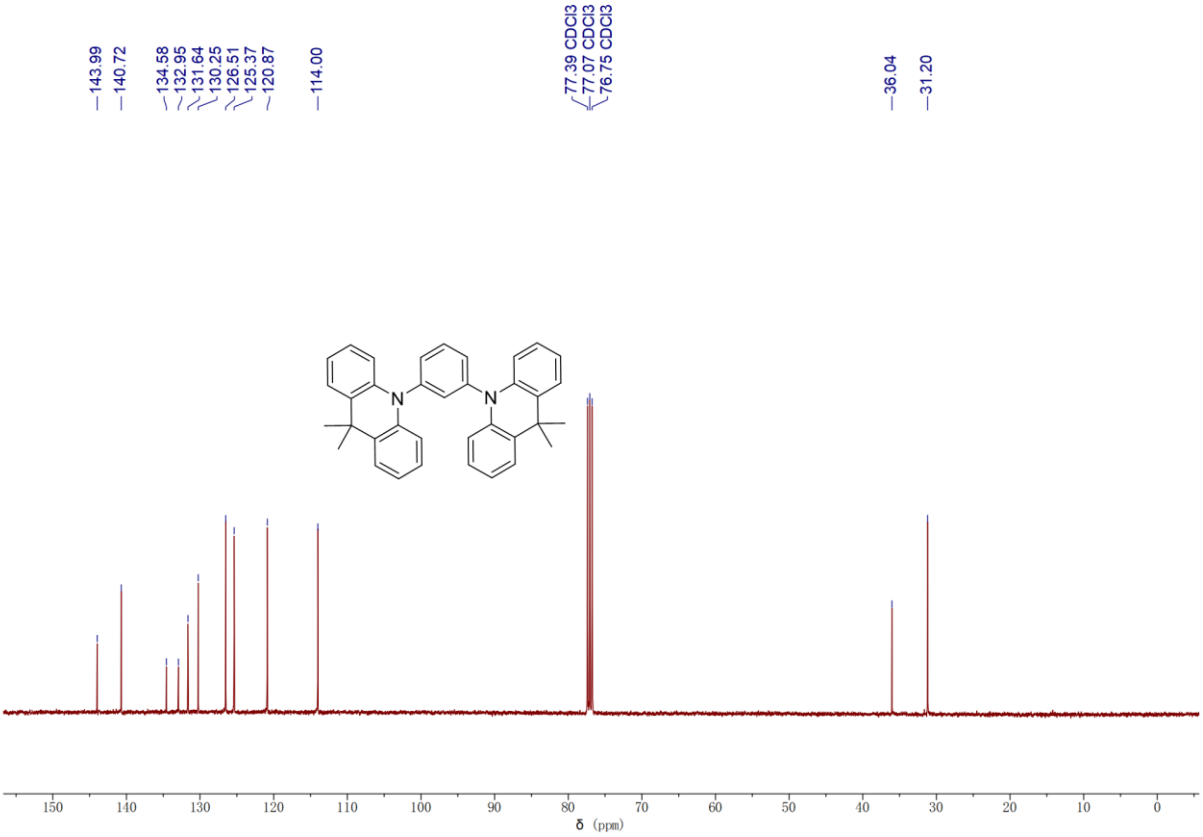


**Figure S57.** $^{13}$C NMR spectrum of *m*-DAc.

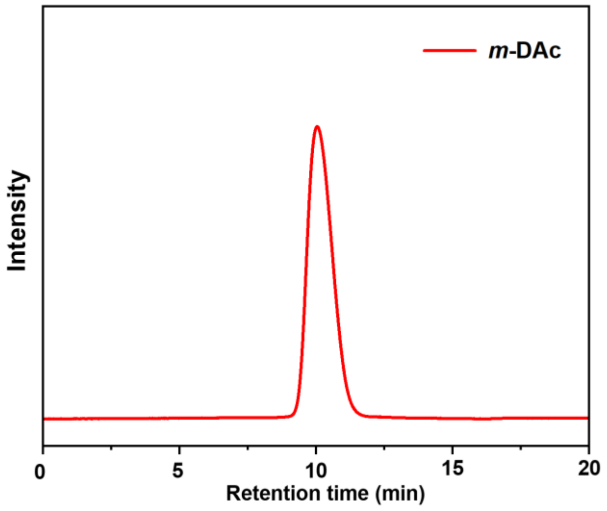


**Figure S58.** High performance liquid chromatogram (HPLC) spectra of *m*-DAc. (Conducted on LaboACE LC-500)

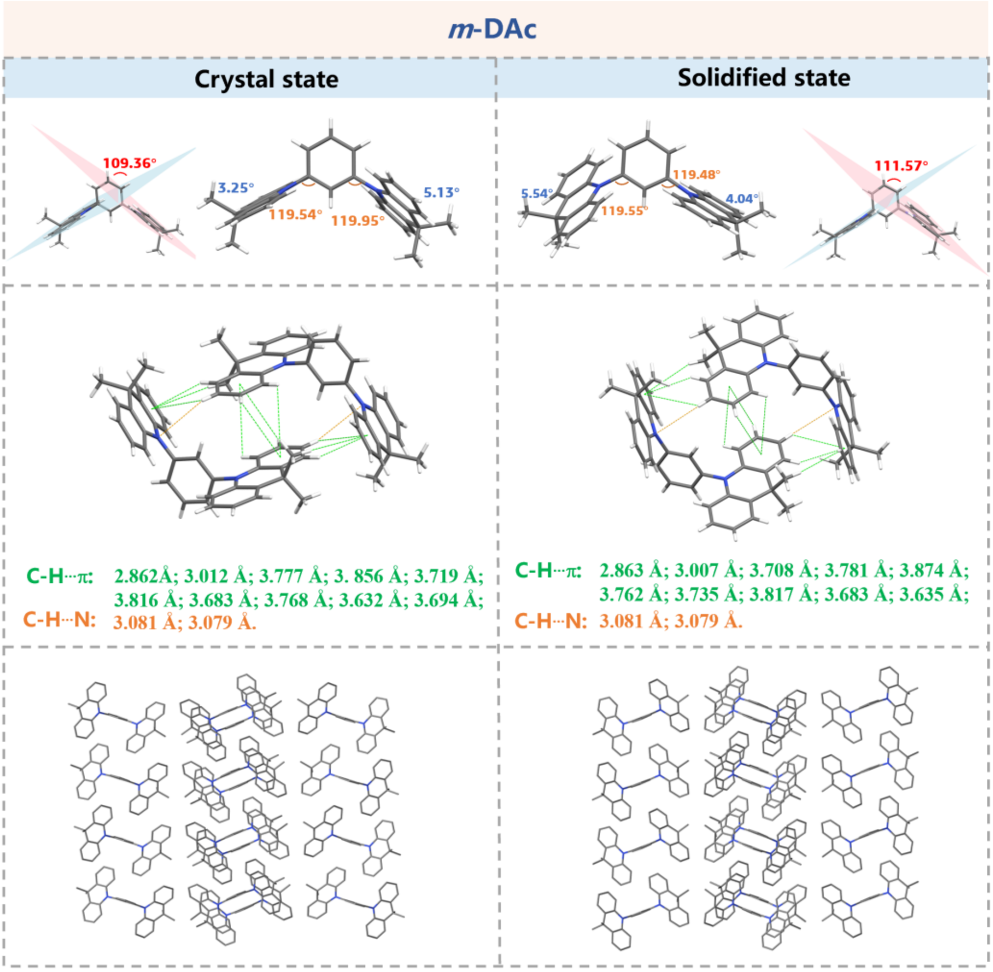


**Figure S59.** Comparison of molecular conformations, intermolecular interactions and packing of *m*-DAc at different aggregated states.

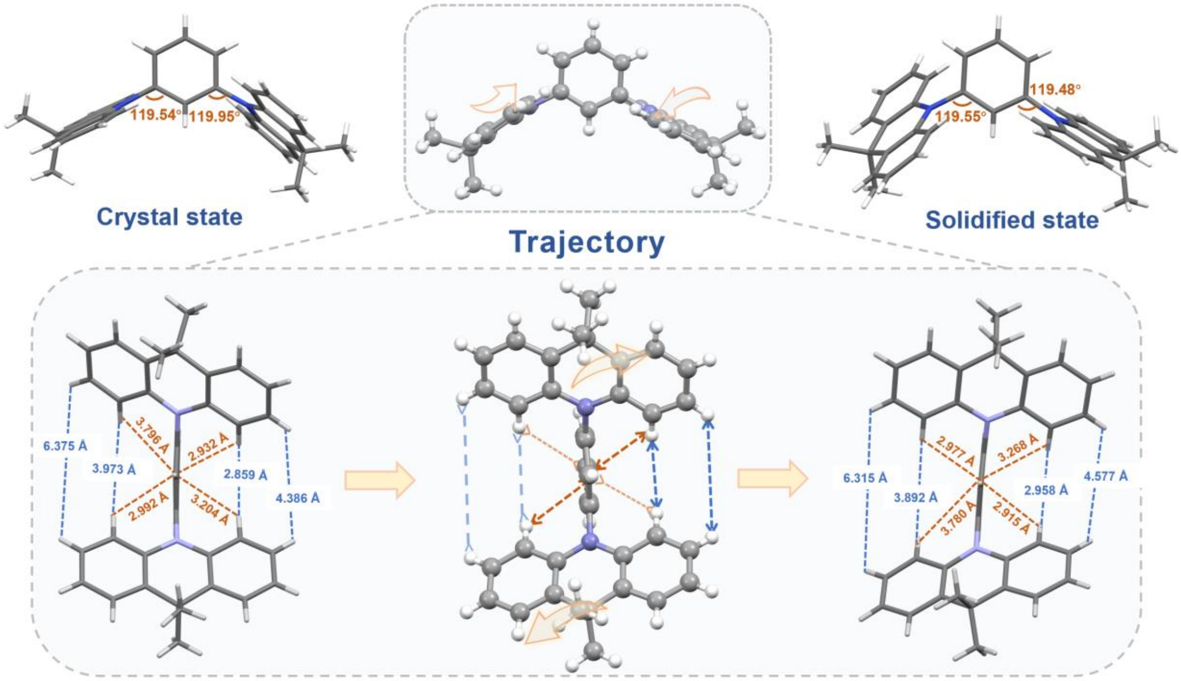


**Figure S60.** Molecular conformations of *m*-DAc in the crystal state and solidified state, together with the trajectory.

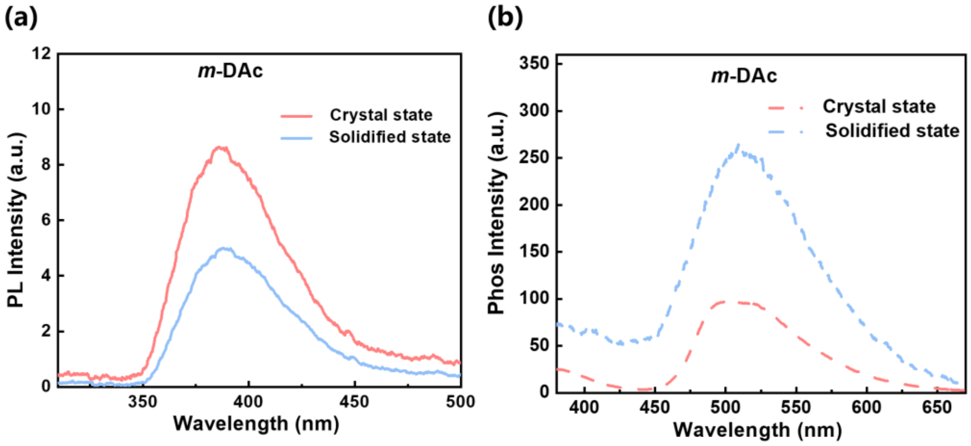


**Figure S61.** (a) PL spectra of *m*-DAc at crystal and solidified states; (b) Phosphorescent spectra of *m*-DAc at crystal and solidified states.

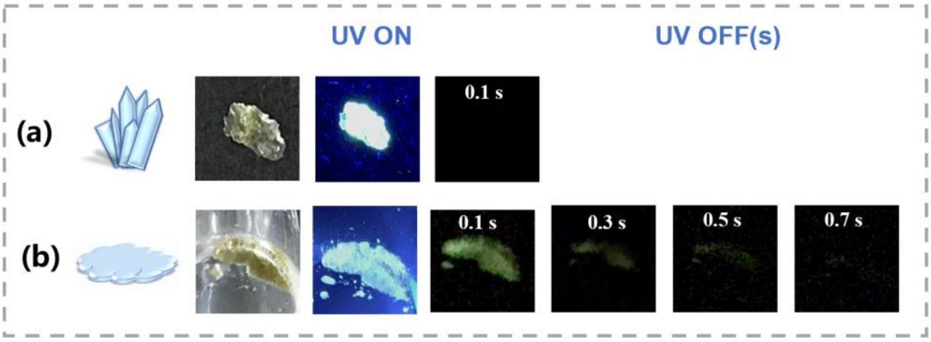


**Figure S62.** Photographs of *m*-DAc taken before and after UV irradiation (365 nm) at room temperature at (a) crystal state and (b) solidified state.

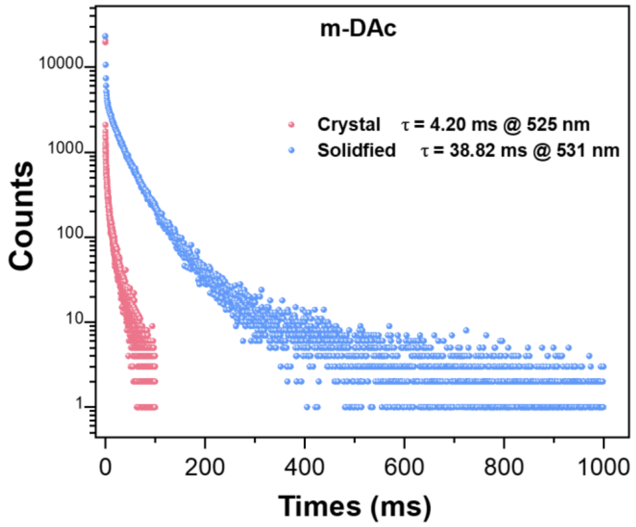


**Figure S63.** Phosphorescence decay of *m*-DAc at crystal and solidified state.

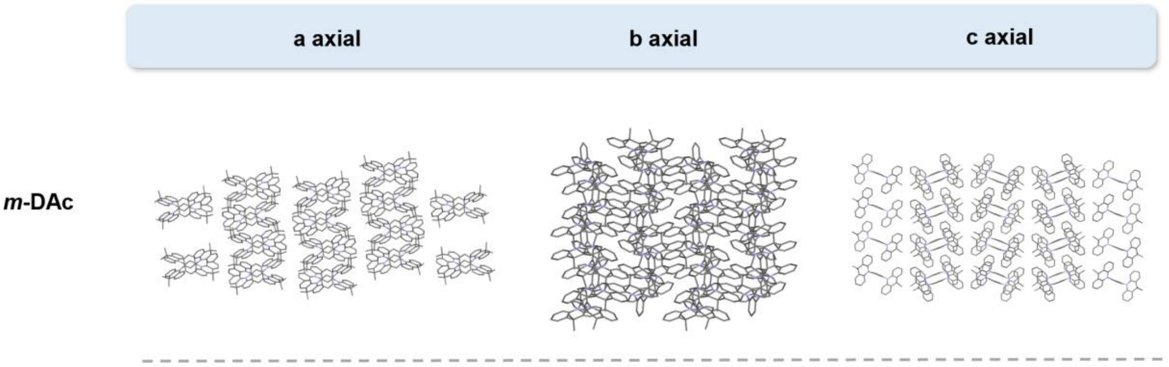


**Figure S64.** Molecular packing in *m*-DAc crystals, observed from the *a*, *b*, and *c* axial directions.

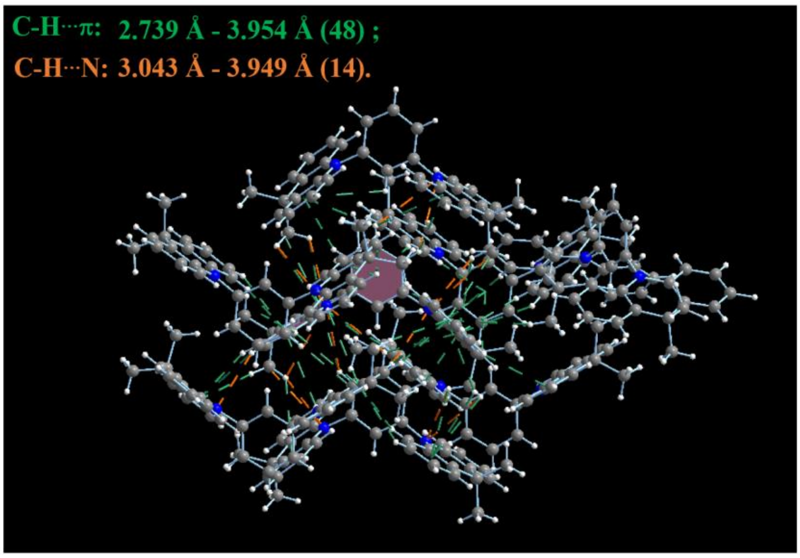


**Figure S65.** The interaction between a given molecule and adjacent molecules in *m*-DAc crystal.

**Table S17.** The interactions between a given molecule and adjacent molecules in *m*-DAc crystal.

| | |
|---|---|
| C-H···π (48) | 2.712 Å; 3.739 Å; 2.862 Å; 2972 Å; 3.012 Å; 3.022 Å; 3.080 Å; 3.092 Å; 3.126 Å (2); 3.191 Å; 3.200 Å (2); 3.269 Å; 3.291 Å; 3.365 Å; 3.399 Å; 3.410 Å; 3.501 Å (2); 3.534 Å (2); 3.540 Å; 3.561 Å; 3.587 Å; 3.605 Å; 3.617 Å; 3.632 Å; 3.633 Å; 3.643 Å; 3.684 Å; 3.694 Å; 3.720 Å; 3.745 Å (2); 3.768 Å; 3.777 Å; 3.789 Å;3.794 Å (2); 3.816 Å; 3.823 Å (2); 3.856 Å; 3.903 Å; 3.919 Å; 3.922 Å; 3.954 Å; |
| C-H···N (14) | 3.043 Å (2); 3.079 Å; 3.613 Å (2); 3.628 Å; 3.704 Å (2); 3.801 Å; 3.847 Å (2); 3.927 Å; 3.949 Å (2). |

**Table S18.** Data of *m*-DAc in the crystal state and solidified state.

| ***m*-DAc** | **Crystal state** | **Solidified state** |
|---|---|---|
| **Formula** | $C_{36}H_{32}N_2$ | $C_{36}H_{32}N_2$ |
| **Crystal system** | orthorhombic | orthorhombic |
| **Space Group** | P 21 21 21 | P 21 21 21 |
| **Cell Lengths (Å)** | 15.8905(10)<br>28.776(2)<br>11.9216(7) | 15.8745(8)<br>28.7709(14)<br>11.9408(4) |
| **Cell Angles (°)** | 90.0<br>90.0<br>90.0 | 90.0<br>90.0<br>90.0 |
| **Cell Volume (Å$^3$)** | 5451.4(6) | 5453.6(4) |
| **Z** | 8 | 8 |
| **Density (g/cm$^3$)** | 1.200 | 1.200 |
| **CCDC** | 2255249 | 2240331 |

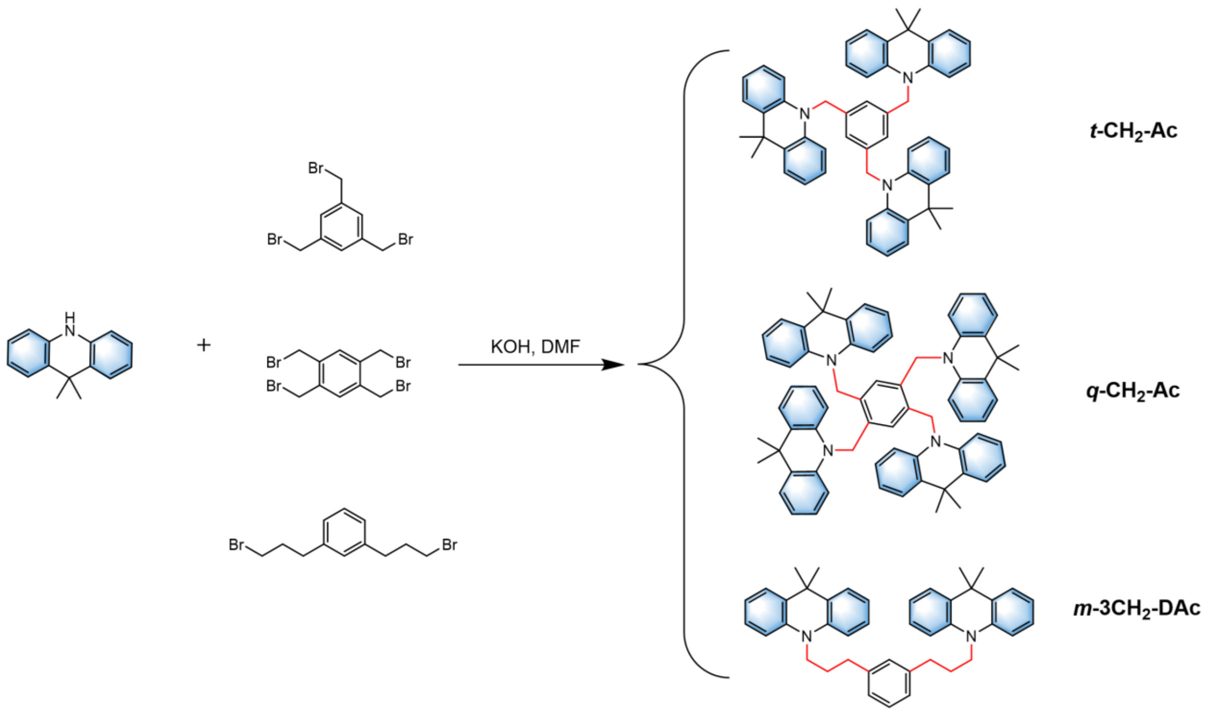


**Figure S66.** The synthetic routes of *t*-$CH_2$-Ac, *q*-$CH_2$-Ac and *m*-3$CH_2$-DAc.

**Synthesis of *t*-$CH_2$-Ac**

9,10-Dihydro-9,9-dimethylacridine (200 mg, 0.96 mmol) and potassium hydroxide (262 mg, 4.70 mol) were placed in a round bottom flask, dissolved with dimethyl sulfoxide (10 mL), and stirred at room temperature for 1 h. 1,3,5-Tris(bromomethyl)benzene (112 mg, 0.32 mmol) was added and stirred at room temperature for another 12 h. Then, a large amount of $H_2O$ was added, and the filter residue was collected after filtration. The crude product was purified by a silica gel column using petroleum ether and dichloromethane as eluent to give ***t*-$CH_2$-Ac** as a light white solid (85 mg, 36%). mp:225. $^1$H NMR (400 MHz, Chloroform-*d*) δ 7.36 (d, *J* = 1.4 Hz, 3H, ArH), 7.34 (d, *J* = 1.3 Hz, 3H, ArH), 6.98 (d, *J* = 1.3 Hz, 1H, ArH), 6.97 (s, 6H, ArH), 6.95 (d, *J* = 1.4 Hz, 2H, ArH), 6.90 (t, *J* = 7.0 Hz, 7H, ArH), 6.61 (d, *J* = 7.9 Hz, 6H, ArH), 5.02 (s, 6H, -$CH_2$), 1.47 (s, 18H, -$CH_3$). $^{13}$C NMR (100 MHz, Chloroform-*d*) δ 140.61, 138.83, 132.08, 126.50, 124.22, 123.38, 120.60, 113.18, 51.02, 36.12, 29.15.

**Synthesis of *q*-$CH_2$-Ac**

9,10-Dihydro-9,9-dimethylacridine (200 mg, 0.96 mmol) and potassium hydroxide (550 mg, 9.80 mmol) were placed in a round bottom flask, dissolved with dimethyl sulfoxide (10 mL), and stirred at room temperature for 1 h. 1,2,4,5-Tetrakis(bromomethyl)benzene (100 mg, 0.22 mmol) were added and stirred at room temperature for 12 h. Then, a large amount of $H_2O$ was added, and then the filter residue was collected after filtration. The crude product was purified by a silica gel column using petroleum ether and dichloromethane as eluent to give ***q*-$CH_2$-Ac** as a light white solid (27.4 mg, 12%). mp: 320 ℃. $^1$H NMR (400 MHz, Chloroform-*d*) δ 7.20 (d, *J* = 7.6 Hz, 8H, ArH), 6.79 (t, *J* = 7.4 Hz, 8H, ArH), 6.73-6.67 (m, 10H, ArH), 6.38 (d, *J* = 8.0 Hz, 8H, ArH), 5.01 (s, 8H-$CH_2$), 1.34 (s, 24H, -$CH_3$). $^{13}$C NMR (101 MHz, Chloroform-*d*) δ 139.84, 132.17, 131.56, 126.18, 123.84, 120.42, 112.58, 35.81.

**Synthesis of *m*-3$CH_2$-DAc**

9,10-Dihydro-9,9-dimethylacridine (53.6 mg, 0.26 mmol) and potassium hydroxide (70 mg, 1.24 mmol) were placed in a round bottom flask, dissolved with dimethyl sulfoxide (10 mL), and stirred at room temperature for 1 h. 1,3-bis(3,bromopropyl) benzene (40 mg, 0.12 mmol) was added and stirred at room temperature for another 12 h. Then, a large amount of $H_2O$ was added, and the filter residue was collected after filtration. The crude product was purified by a silica gel column using petroleum ether and dichloromethane as eluent to give ***m*-3$CH_2$-DAc** as a colorless oil (41.2 mg, 58.4%). $^1$H NMR (400 MHz, Chloroform-*d*) δ 7.39 (dd, *J* = 7.8, 1.6 Hz, 4H, ArH), 7.27 (d, *J* = 5.2 Hz, 1H, ArH), 7.16-7.08 (m, 7H, ArH), 6.94-6.83 (m, 9H, ArH), 3.99-3.90 (m, 4H, -$CH_2$), 2.77 (t, *J* = 7.5 Hz, 4H, -$CH_2$), 2.19 (q, *J* = 7.7 Hz, 4H, -$CH_2$), 1.52 (s, 12H, -$CH_3$).

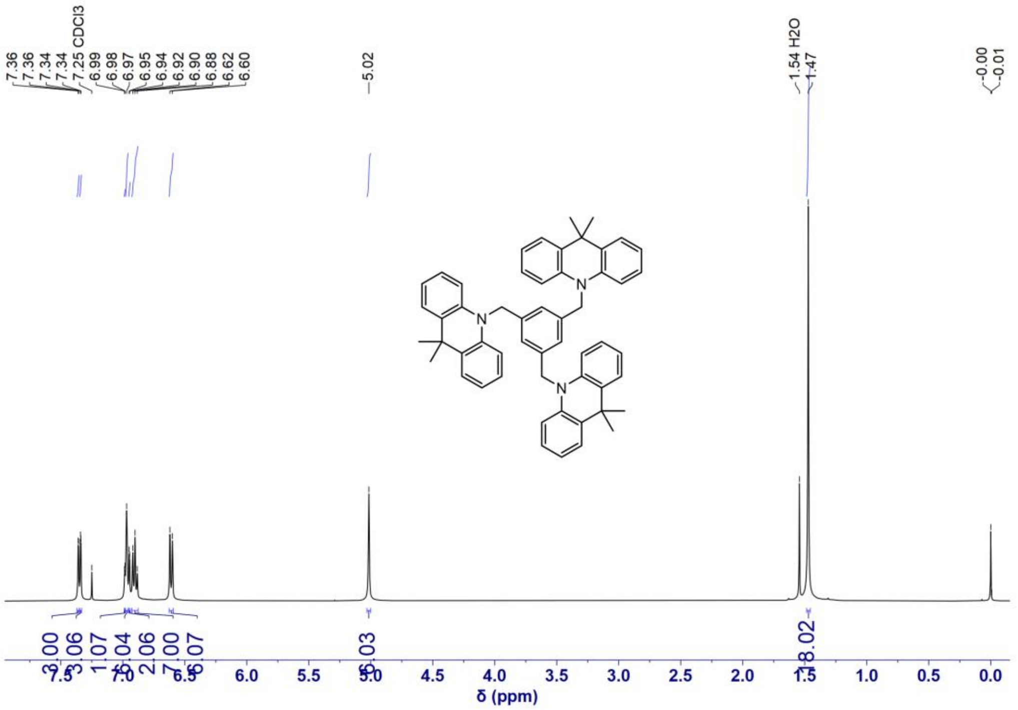

**Figure S67.** $^{1}$H NMR spectrum of *t*-$CH_2$-Ac.

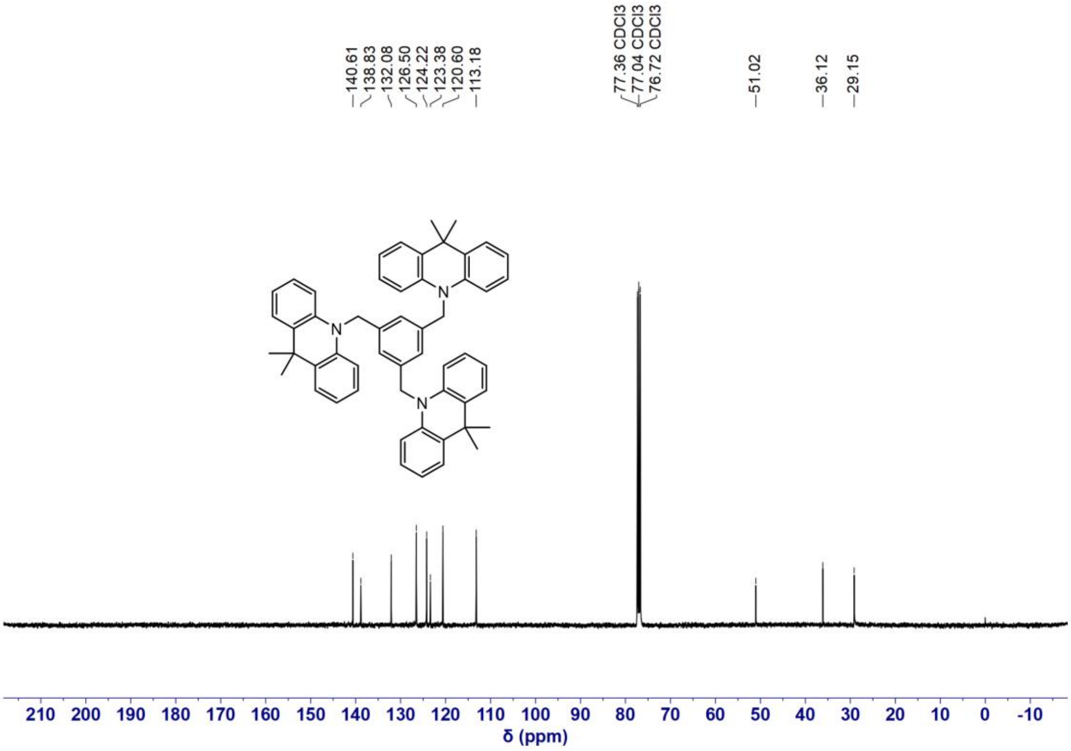

**Figure S68.** $^{13}$C NMR spectrum of *t*-$CH_2$-Ac.

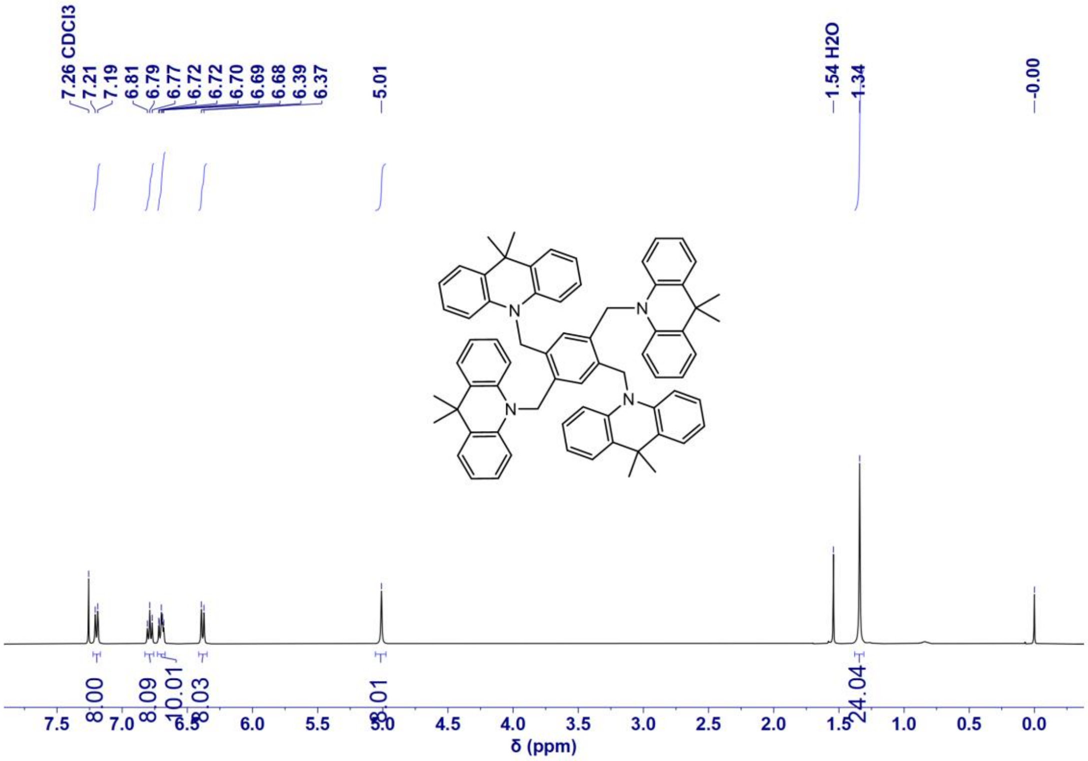


**Figure S69.** $^{1}H$ NMR spectrum of *q*-$CH_2$-Ac.

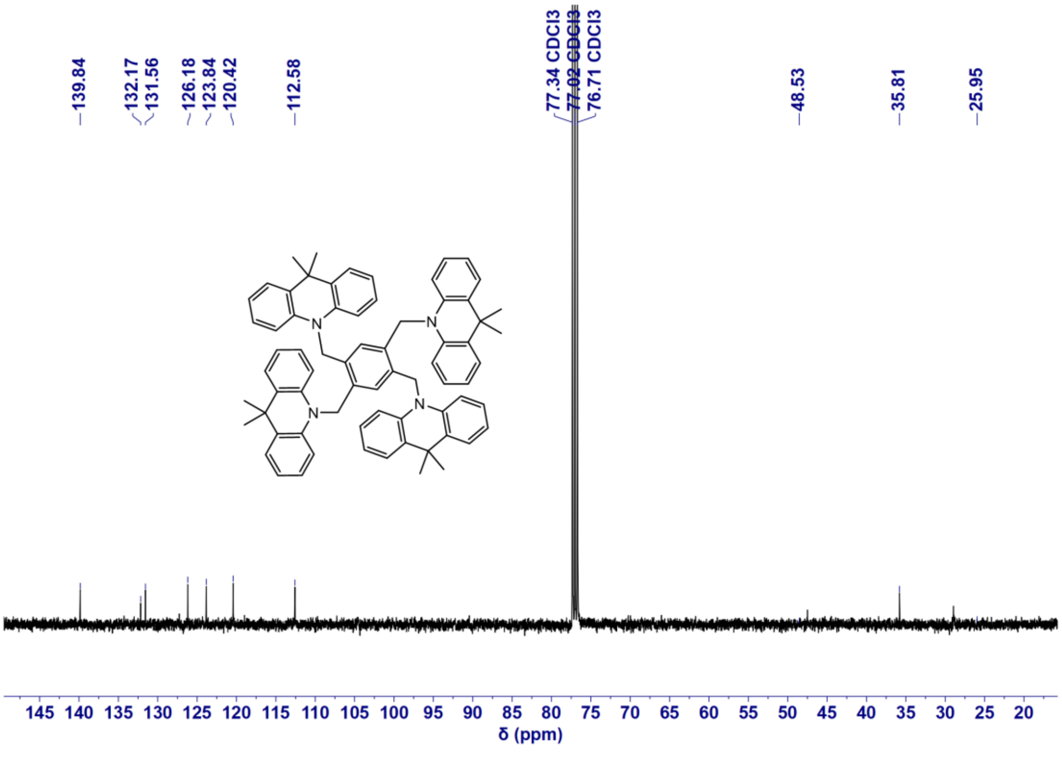


**Figure S70.** $^{13}C$ NMR spectrum of *q*-$CH_2$-Ac.

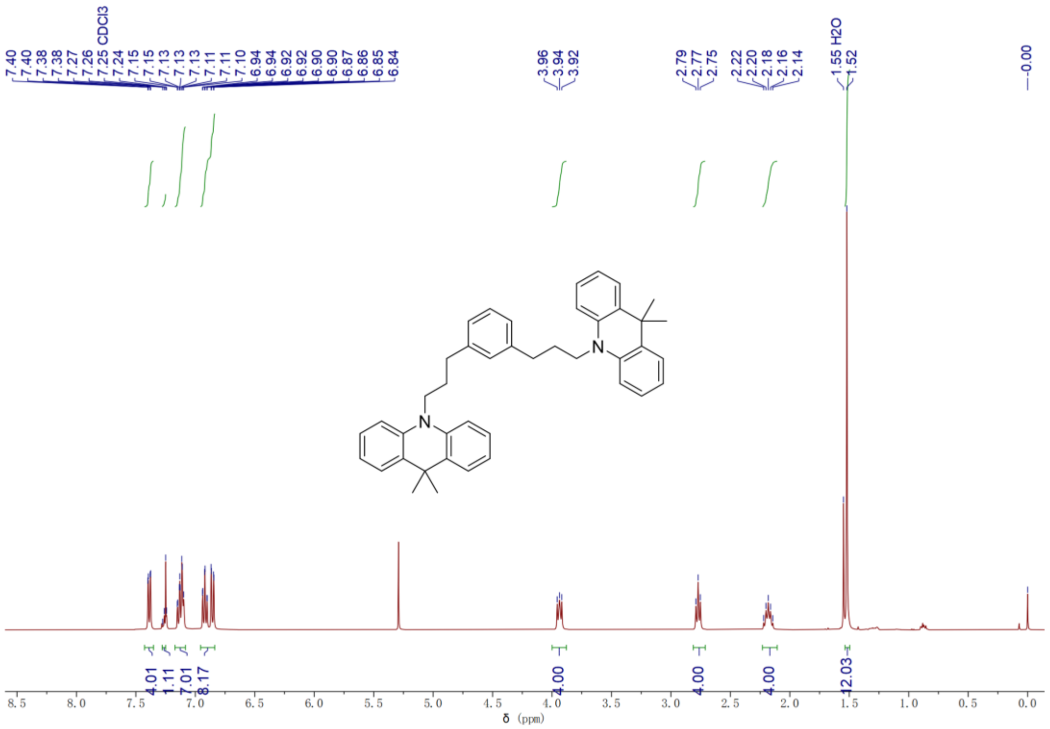

**Figure S71.** $^{1}$H NMR spectrum of *m*-3CH$_2$-DAc.

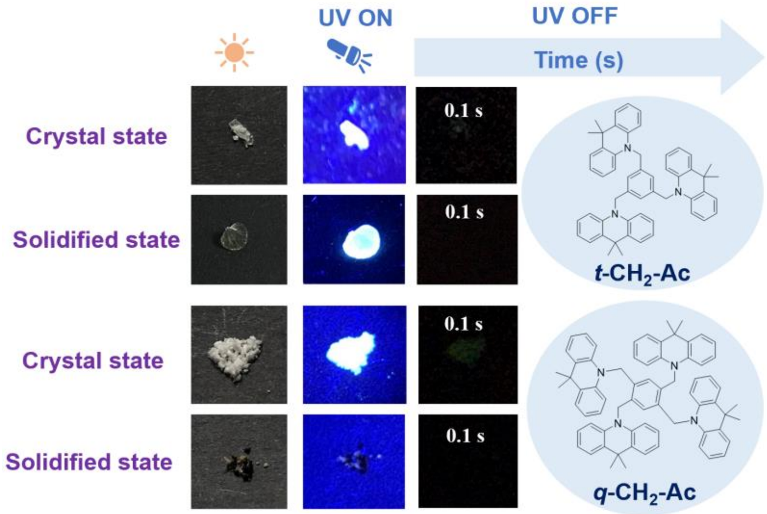


**Figure S72**. Photographs of *t*-CH$_2$-Ac and *q*-CH$_2$-Ac at crystal and solidified states taken before and after UV irradiation (365 nm) under ambient conditions.

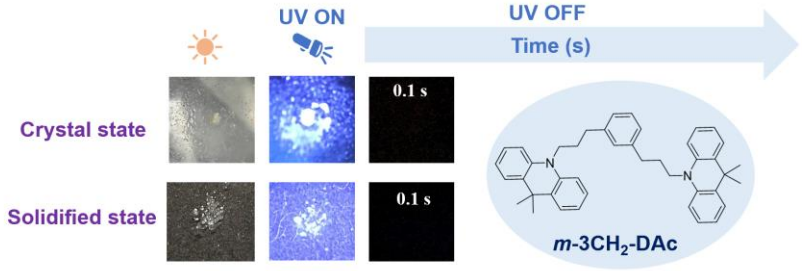


**Figure S73** Photographs of *m*-3CH$_2$-DAc at crystal and solidified states taken before and after UV irradiation (365 nm) under ambient conditions.

**Video S1.** Optimized thermal-annealing process

**Video S2.** Trajectory of molecule *o*-CH$_2$-DAc

**Video S3.** Trajectory of molecule *m*-CH$_2$-DAc

**Video S4.** Repeatability of thermal annealing-promoted RTP property
